# T cell–intrinsic prostaglandin E_2_-EP2/EP4 signaling is critical in pathogenic T_H_17 cell–driven inflammation

**DOI:** 10.1016/j.jaci.2018.05.036

**Published:** 2019-02

**Authors:** Jinju Lee, Tomohiro Aoki, Dean Thumkeo, Ratklao Siriwach, Chengcan Yao, Shuh Narumiya

**Affiliations:** a Core Research for Evolutional Science and Technology (CREST), Medical Innovation Center, Kyoto, Japan; c Center for Innovation in Immunoregulation Technology and Therapeutics, Kyoto University Graduate School of Medicine, Kyoto, Japan; b Kyoto University, Graduate School of Biostudies, Kyoto, Japan; d Medical Research Council (MRC) Centre for Inflammation Research, Queen's Medical Research Institute, University of Edinburgh, Edinburgh, United Kingdom

**Keywords:** Psoriasis, pathogenic T_H_17 cells, IL-23 receptor, prostaglandin E_2_, prostaglandin E receptor EP2, prostaglandin E receptor EP4, signal transducer and activator of transcription 3, cAMP-responsive element binding protein 1, nuclear factor κ light chain enhancer of activated B cells, cAMP, Cyclic AMP, CREB, cAMP-responsive element binding protein, db-cAMP, Dibutyryl cAMP, Epac, Exchange factor directly activated by cyclic AMP, IL-12R, IL-12 receptor, IL-23R, IL-23 receptor, JAK, Janus kinase, KO, Knockout, mPGES, Membrane-associated PGE synthase, NF-κB, Nuclear factor κ light chain enhancer of activated B cells, PG, Prostaglandin, PKA, Protein kinase A, STAT, Signal transducer and activator of transcription, WT, Wild-type

## Abstract

**Background:**

IL-23 is the key cytokine for generation of pathogenic IL-17–producing helper T (T_H_17) cells, which contribute critically to autoimmune diseases. However, how IL-23 generates pathogenic T_H_17 cells remains to be elucidated.

**Objectives:**

We sought to examine the involvement, molecular mechanisms, and clinical implications of prostaglandin (PG) E_2_–EP2/EP4 signaling in induction of IL-23–driven pathogenic T_H_17 cells.

**Methods:**

The role of PGE_2_ in induction of pathogenic T_H_17 cells was investigated in mouse T_H_17 cells in culture *in vitro* and in an IL-23–induced psoriasis mouse model *in vivo*. Clinical relevance of the findings in mice was examined by using gene expression profiling of IL-23 and PGE_2_-EP2/EP4 signaling in psoriatic skin from patients.

**Results:**

IL-23 induces *Ptgs2*, encoding COX2 in T_H_17 cells, and produces PGE_2_, which acts back on the PGE receptors EP2 and EP4 in these cells and enhances IL-23–induced expression of an IL-23 receptor subunit gene, *Il23r*, by activating signal transducer and activator of transcription (STAT) 3, cAMP-responsive element binding protein 1, and nuclear factor κ light chain enhancer of activated B cells (NF-κB) through cyclic AMP–protein kinase A signaling. This PGE_2_ signaling also induces expression of various inflammation-related genes, which possibly function in T_H_17 cell–mediated pathology. Combined deletion of EP2 and EP4 selectively in T cells suppressed accumulation of IL-17A^+^ and IL-17A^+^IFN-γ^+^ pathogenic Th17 cells and abolished skin inflammation in an IL-23–induced psoriasis mouse model. Analysis of human psoriatic skin biopsy specimens shows positive correlation between PGE_2_ signaling and the IL-23/T_H_17 pathway.

**Conclusions:**

T cell–intrinsic EP2/EP4 signaling is critical in IL-23–driven generation of pathogenic T_H_17 cells and consequent pathogenesis in the skin.

## Graphical abstract

**Figure:**
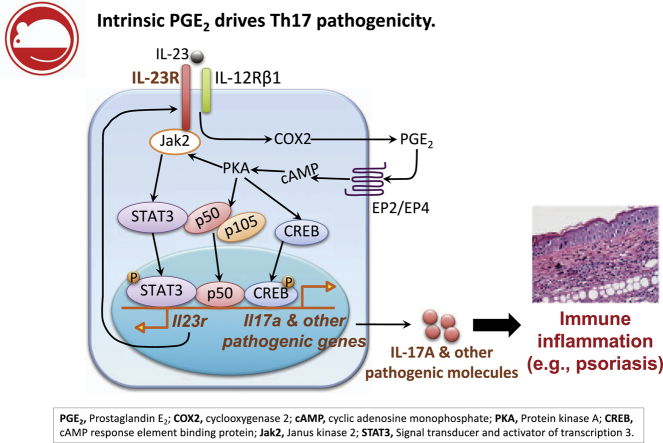

CD4^+^ T cells differentiate into T_H_1, T_H_2, and T_H_17 cells in response to the specific cytokine milieu present in the microenvironment of inflammation and mediate immune inflammatory responses in respective settings.^1^, ^2^, ^3^, ^4^ Among these T_H_ subsets, T_H_17 cells mediate inflammatory responses in patients with many autoimmune diseases, including multiple sclerosis; inflammatory bowel diseases, such as Crohn disease; psoriasis; and rheumatoid arthritis. The importance of T_H_17 cells in these processes was suggested first in animal models of these diseases, including experimental autoimmune encephalomyelitis, and an IL-23– or imiquimod-induced psoriasis model,^5^, ^6^, ^7^, ^8^, ^9^ and was validated recently by clinical effectiveness of antibodies targeting IL-23 in patients with psoriasis.^10^, ^11^, ^12^, ^13^, ^14^

Differentiation of T_H_17 cells from naive CD4^+^ T cells is driven by the combined actions of IL-6 and TGF-β1.^15^, ^16^, ^17^, ^18^, ^19^ However, differentiated T_H_17 cells have little capacity to induce autoimmune and inflammatory pathology.^20^ It should be noted that these T_H_17 cells exhibit plasticity and could transdifferentiate into other effector T-cell types or even regulatory T cells under certain contexts, such as inflammation or autoimmune disease.^21^, ^22^, ^23^

Accumulating evidence suggests that T cell–intrinsic IL-23 signaling not only increases IL-17 production of T_H_17 cells but also plays a crucial role in inducing and stabilizing their pathogenicity.^20^, ^24^, ^25^, ^26^, ^27^ It is known that IL-23 acts on IL-23 receptor (IL-23R) complex composed of IL-23R and IL-12 receptor (IL-12R) β1, activates signal transducer and activator of transcription (STAT) 3, and induces expression of *Il23r*, thus forming the self-amplification loop. The pathophysiologic importance of this IL-23–IL-23R signaling has been indicated by several genomic studies that showed a positive correlation between single nucleotide polymorphisms of genes involved in this pathway, such as *IL23R*, *IL12B (p40)*, Janus kinase 2 *(JAK2)*, and *STAT3*, and a wide range of IL-17–dependent autoimmune diseases.^28^, ^29^, ^30^

Although it was shown that IL-23 signaling induces expression of T_H_17 pathogenic signature genes through activation of STAT3,^31^, ^32^ transcription factors other than STAT3 are also implicated for induction of pathogenic T_H_17 cells because IL-6, which activates STAT3 similarly to IL-23, cannot induce IL-23R gene expression.^32^ Therefore, the identity of additional transcriptional factors and regulatory mechanisms are important issues to be defined. Moreover, how IL-23 cooperates with other inflammatory factors formed in the disease microenvironment and the importance of such cooperation for pathogenic conversion of T_H_17 cells and overall pathology remain largely obscure. Clarification of these points could provide a new opportunity to develop small-molecule drugs as therapeutic alternatives to anti–IL-23 antibodies without systemic immune suppression. Biological agents might additionally cause unpredictable adverse events^33^ and can be costly on long-term use.^34^ It should also be mentioned that JAK inhibitors that are now being evaluated for their efficacy in patients with autoimmune diseases are presumably not free from adverse effects either because of their effects on general immune suppression.^35^

Prostanoids, including prostaglandin (PG) D_2_, PGE_2_, PGF_2α_, PGI_2_, and thromboxane A_2_, are oxygenated metabolites of arachidonic acid produced by sequential actions of COX and respective synthases and act on their cognate receptors, DP for PGD_2_, EP1 to 4 for PGE_2_, IP for PGI_2_, FP for PGF_2α_, and TP for thromboxane A_2_, to exert their actions.^36^ Although prostanoids were regarded previously as immunosuppressants,^37^, ^38^ recent studies have revealed their immunostimulatory actions in processes such as cytokine production, dendritic cell maturation, macrophage activation, and differentiation and expansion of T_H_ cell subsets.^39^, ^40^, ^41^ Indeed, PGE_2_-EP2 and EP4 (EP2/EP4) signaling enhances T_H_1 differentiation by inducing expression of the IL-12R subunit *Il12rb2* and the IFN-γ receptor *Ifngr1*, thus facilitating IL-12 signaling and T_H_1 differentiation.^42^, ^43^ Notably, this PGE_2_-EP2/EP4 signaling was also reported to synergize with IL-23 to facilitate T_H_17 cell expansion both in murine and human T cells.^43^, ^44^, ^45^ However, whether and how PGE_2_-EP2/EP4 signaling is involved in induction of pathogenic T_H_17 cells remain unknown.

In this study we have examined how PGE_2_-EP2/EP4 signaling and IL-23 stimulation together regulate the generation of pathogenic T_H_17 cells. Through this analysis, we have identified the transcription mechanisms in addition to STAT3 that regulate *Il23r* expression and T_H_17 pathogenicity. We have further clarified the importance of PGE_2_ signaling in T_H_17-mediated immune inflammation *in vivo* and found a correlation between PGE_2_-EP2/EP4 signaling and IL-23–IL-23R signaling in biopsy samples from patients with psoriasis.

## Methods

### Mice

All animal experiments were approved by the Institutional Animal Care and Use Committee of Kyoto University Graduate School of Medicine and complied with the National Institutes of Health's “Guide for the care and use of laboratory animals”. C57BL/6NCrSlc mice were purchased from Shimizu laboratory, and Lck-Cre and B6.Cg-*Nfkb1*^*tm1Bal*^/J mice were purchased from the Jackson Laboratory (Bar Harbor, Me). Mice deficient in *Ptger2*^46^ and mice with floxed *Ptger2*^47^ were established in our laboratory. Mice with floxed *Ptger4* were a kind gift of Richard Breyer.^48^

### Psoriasis models

Mice were injected subcutaneously with IL-23 (500 ng; #130-096-677; Miltenyi Biotec, Bergisch Gladbach, Germany) once a day in one ear and with PBS in the contralateral ear as a control to induce psoriasis-like lesions in the ear in an IL-23–induced psoriasis mouse model. In an imiquimod-induced psoriasis mouse model, Baselna cream containing 10% imiquimod was applied onto the ears of mice once a day. Ear thickness was then measured with a digital micrometer (#KM-BMB1-25; Mitutoyo, Kawasaki, Japan) every other day. In some experiments an antagonist for EP4, AS1954813,^49^ suspended in 0.5% methylcellulose was administered orally twice a day, or indomethacin and SC-236 were administered in drinking water during the experimental period.

See the Methods section in this article's Online Repository at www.jacionline.org for further details.

## Results

### IL-23 mobilizes the endogenous COX2-PGE_2_-EP2/EP4 signaling that enhances induction of *Il23r* expression in T_H_17 cells

**Fig 1:**
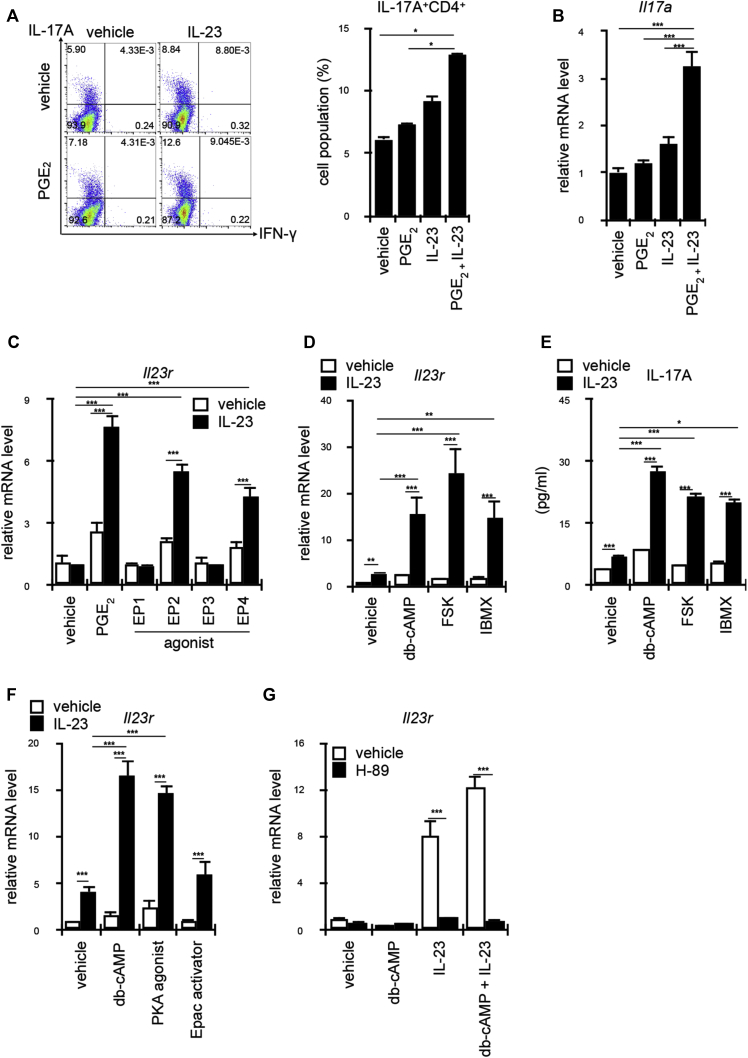
IL-23 mobilizes the endogenous PGE_2_-EP2/EP4-cAMP-PKA pathway to facilitate T_H_17 expansion through synergistic *Il23r* induction. **A** and **B,** Expansion of the T_H_17 population by PGE_2_ and IL-23. CD4^+^ T cells were differentiated with TGF-β1 and IL-6 to T_H_17 cells for 4 days and then stimulated with 100 nmol/L PGE_2_ in the absence or presence of IL-23 (10 ng/mL) for an additional 3 days. The cells were examined by using fluorescence-activated cell sorting for IL-17A and IFN-γ (Fig 1, *A*) and by using quantitative RT-PCR for *Il17a* expression (Fig 1, *B*). **C-E,** Effects of PGE_2_, 100 μM of an agonist selective to each EP subtype, and related compounds on *Il23r* expression. T_H_17 cells were incubated with 100 nmol/L PGE_2_, an agonist selective to each EP subtype, ONO-DI-004 (EP1), ONO-AE1-259 (EP2), ONO-AE-248 (EP3), or ONO-AE1-329 (EP4), 100 μmol/L db-cAMP, 10 μmol/L forskolin *(FSK)*, or 100 μmol/L 3-isobutyl-1-methylxanthine *(IBMX)* with or without IL-23. *Il23r* expression (Fig 1, *C* and *D*) or IL-17A concentrations in culture supernatants (Fig 1, *E*) were examined. **F** and **G,** Expression of *Il23r* in T_H_17 cells stimulated with 100 μmol/L db-cAMP, 300 μmol/L N6-Bnz-cAMP (a PKA agonist), 300 μmol/L 8-pCTP-2′-O-Me-cAMP (an Epac activator; Fig 1, *F*), or 10 μM H-89 (a PKA inhibitor; Fig 1, *G*) with or without IL-23. All *bars* indicate means ± SEMs (n = 3). *P < .05, **P < .01, and ***P < .001.

Given the previous findings^43^, ^44^, ^45^ that PGE_2_-EP2/EP4 signaling enhances IL-23–induced T_H_17 cell expansion, we questioned whether and how this signaling contributes to pathogenic T_H_17 cell generation by IL-23. To investigate this issue, we first cultured CD4^+^ T cells from mouse spleens under T_H_17-skewing conditions (IL-6 plus TGF-β1) for 4 days and then incubated with IL-23 for an additional 3 days. Consistent with our previous findings,^43^ addition of PGE_2_ to the latter culture significantly enhanced IL-23–induced expansion and *Il17a* expression of T_H_17 cells (Fig 1, *A* and *B*). Interestingly, we also noted that PGE_2_ markedly upregulated IL-23–induced expression of *Il23r*, which was mimicked by both EP2- and EP4-selective agonists (Fig 1, *C*). Because both EP2 and EP4 activate protein kinase A (PKA) and exchange factor directly activated by cyclic AMP (Epac) by increasing levels of intracellular cyclic AMP (cAMP),^36^ we examined effects of compounds acting on these signaling and found that the cAMP analogue dibutyryl cAMP (db-cAMP), forskolin (FSK), and the phosphodiesterase inhibitor 3-isobutyl-1-methylxanthine all synergized with IL-23 and significantly amplified IL-23–induced *Il23r* expression and IL-17A production in these cells (Fig 1, *D* and *E*). Furthermore, enhancement of *Il23r* expression was reproduced by a PKA agonist (N6-Bnz-cAMP, 300 μmol/L) but not an Epac activator (8-pCTP-2′-O-Me-cAMP, 300 μmol/L; Fig 1, F) and was ameliorated consistently by treatment with the PKA inhibitor H-89 (10 μmol/L; Fig 1, *G*).

**Fig 2:**
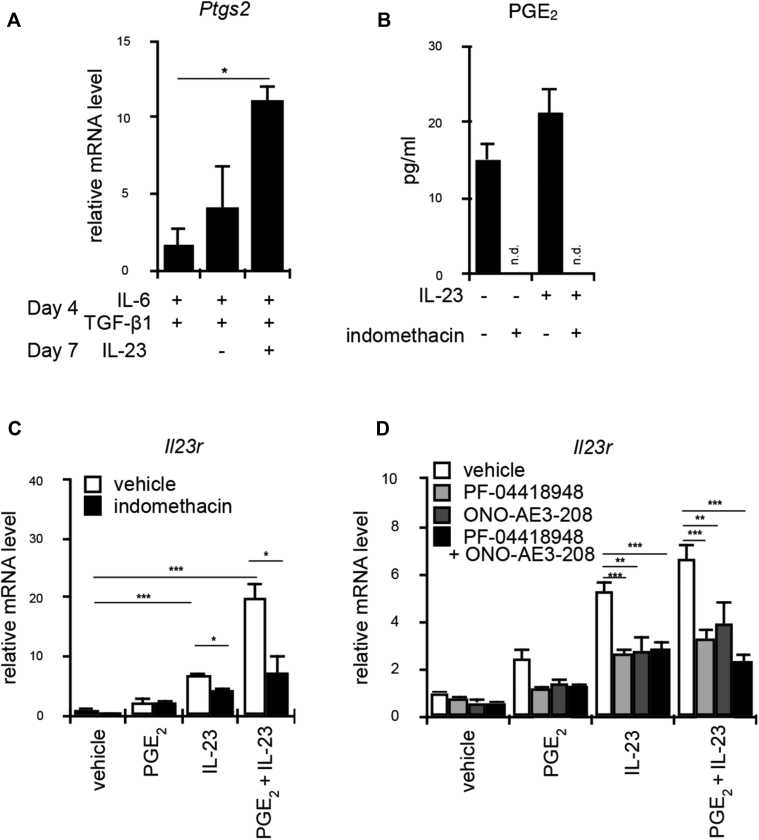
IL-23 self-amplifies its own signaling through a T cell–intrinsic positive feedback COX2–PGE_2_–cAMP–IL-23R loop. **A,** Expression of COX2 mRNA *(Ptgs2)* in T_H_17 cells or T_H_17 cells cultured further in the presence or absence of IL-23 for 3 days, as determined by using quantitative RT-PCR. **B,** Concentrations of PGE_2_ in culture supernatants of T_H_17 cells in the presence or absence of IL-23 and indomethacin determined by means of ELISA. *n.d.*, Not detected. **C,***Il23r* expression in T_H_17 cells stimulated with PGE_2_ and IL-23 in the presence or absence of 100 μM indomethacin for 3 days. **D,***Il23r* expression in T_H_17 cells stimulated with PGE_2_ and IL-23 in the presence or absence of EP2 (PF-04418948, 300 μM) and/or EP4 (ONO-AE3-208, 100 μM) antagonists for 3 days. All *bars* indicate means ± SEMs (n = 3). *P < .05, **P < .01, and ***P < .001.

Notably, IL-23 stimulation significantly increased *Ptgs2* (COX2) gene expression in T_H_17 cells (Fig 2, *A*) and produced subnanomolar concentrations of PGE_2_ in culture medium (Fig 2, *B*). Moreover, incubation with a nonselective COX inhibitor (indomethacin, 100 μmol/L) or a selective COX2 inhibitor (SC-236, 100 μmol/L) but not a selective COX-1 inhibitor (SC-560, 100 μmol/L) significantly blocked induction of *Il23r* expression in response to both IL-23 alone and IL-23 and PGE_2_ in combination (Fig 2, *C*, and see Fig E1, *A*, in this article's Online Repository at www.jacionline.org). In addition, antagonists selective to EP2 (PF-04418948) or EP4 (ONO-AE3-208) also suppressed *Il23r* expression (Fig 2, *D*). Intriguingly, indomethacin and SC-236 suppressed expression of *Il23r* induced by IL-23 and PGE_2_ to the level that these inhibitors achieved in the presence of IL-23 alone, suggesting that they canceled the effect of exogenously added PGE_2_ (Fig 2, *D*, and see Fig E1, *A*). Given that PGE_2_ added to the culture medium degrades time dependently,^50^ these results suggest that exogenously added PGE_2_ induces COX2 and produces PGE_2_ endogenously and continuously, as we reported previously,^51^ which makes more contribution to *Il23r* induction, and that indomethacin and COX2 inhibitor block this process. Indeed, the addition of stable EP2 and EP4 agonists overcame the *Il23r* suppression by indomethacin (see Fig E1, *B*). Therefore, these data together suggest that IL-23 stimulates T_H_17 cells to produce PGE_2_, which acts back to EP2 and EP4 on these cells to augment *Il23r* expression in a positive feedback manner.

### Induction of *Il23r* expression by IL-23 and PGE_2_-cAMP signaling is mediated through not only STAT3 but also cAMP-responsive element binding protein 1 and nuclear factor κ light chain enhancer of activated B cells

**Fig 3:**
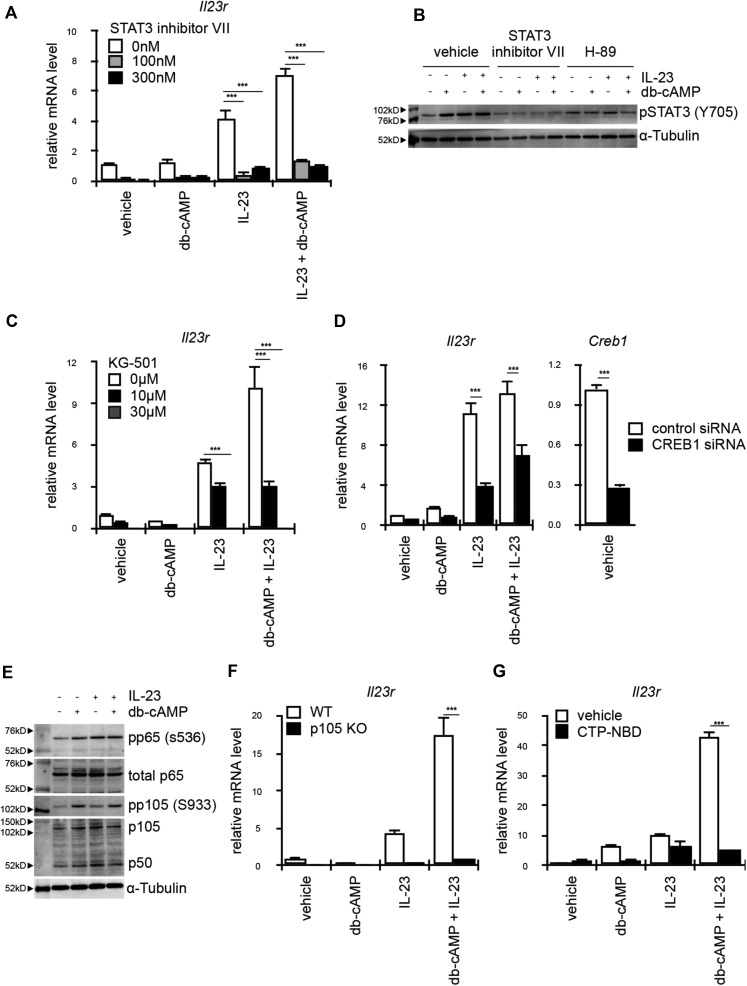
STAT3, CREB1, and NF-κB mediate cAMP- and IL-23–induced *Il23r* expression in T_H_17 cells. **A,** Effect of STAT3 inhibitor VII on *Il23r* expression in T_H_17 cells stimulated with db-cAMP, IL-23, or both for 3 days. **B,** Western blot analysis of phospho-Y705 STAT3 and α-tubulin as a loading control in T_H_17 cells cultured as described in the Methods section in this article's Online Repository. Representative images from 2 independent experiments are shown. **C,** Effect of KG-501 on *Il23r* expression in T_H_17 cells stimulated by using db-cAMP, IL-23, or both. **D,** Effects of RNA interference for CREB1 on *Il23r* expression *(left)* and *Creb1* expression to confirm CREB knockdown efficiency *(right)*. RNA interference, subsequent culture, and stimulation of T_H_17 cells were performed, as described in the Methods section in this article's Online Repository. **E,** Western blot analysis of phospho-S536 NF-κB p65 (pp65), phospho-S933 NF-κB p105 (pp105), p65, p105/p50, and α-tubulin in T_H_17 cells stimulated as described in the Methods section in this article's Online Repository. Representative images from 2 independent experiments are shown. **F** and **G,** Effects of p105 KO (Fig 3, *F*) or 50 μM CTP-NBD (Fig 3, *G*) on *Il23r* expression in T_H_17 cells stimulated with db-cAMP, IL-23, or both for 3 days. All *bars* indicate means ± SEMs (n = 3 for Fig 3, *A*, *C*, *F*, and *G*; n = 18 in Fig 3, *B*). ***P < .001.

We then investigated transcription factors responsible for induction of *Il23r* expression in T_H_17 cells by IL-23 and PGE_2_-EP2/EP4 signaling. Because IL-23 activates STAT3 to induce *Il23r* expression,^52^ we first examined the effect of a STAT3 inhibitor. Addition of STAT3 inhibitor VII suppressed *Il23r* expression not only by IL-23 but also by db-cAMP and their combination (Fig 3, *A*), indicating that db-cAMP action was also mediated by STAT3. Consistently, Y705 phosphorylation of STAT3 was increased by db-cAMP at 5 and 30 minutes (see Fig E2, *A*, in this article's Online Repository at www.jacionline.org), which was ameliorated not only by addition of STAT3 inhibitor VII but also by H-89 (Fig 3, *B*), indicating the involvement of PKA in db-cAMP–mediated Y705 phosphorylation of STAT3. Intriguingly, Y1007/1008 phosphorylation of JAK2, a kinase responsible for STAT3 Y705 phosphorylation in T_H_17 cells, was enhanced by db-cAMP, and this enhancement was suppressed by Src kinase inhibitor I (see Fig E2, *B*), indicating cAMP-PKA activates STAT3 through the c-Src–JAK2 pathway.

Although the above findings demonstrated that IL-23 and PGE_2_-cAMP signaling converge at STAT3 activation, it is well known that other STAT3 activators, such as IL-6 and IL-21, cannot substitute for IL-23 in expansion of the T_H_17 population,^32^ indicating that STAT3 is not the sole transcription factor regulating expression of *Il23r*.

Because PKA activates cAMP-responsive element binding protein (CREB) 1,^36^ we investigated the involvement of CREB1 in *Il23r* expression. Both KG-501, a CREB1 inhibitor,^53^ and RNA interference for CREB1 suppressed *Il23r* induction in response to db-cAMP, IL-23, or both, suggesting the involvement of CREB1 in *Il23r* expression in T_H_17 cells (Fig 3, *C* and *D*). Because IL-23 signaling enhances endogenous PGE_2_ production through induction of COX2 expression in T_H_17 cells (Fig 2, *A* and *B*), suppression of *Il23r* expression by inhibition or depletion of CREB1 could be due to inhibition of this endogenous PGE_2_ signaling for *Il23r* induction.

Furthermore, we detected an increase in S536 phosphorylation of NF-κB p65 (pp65) in response to db-cAMP, IL-23, or both at 24 hours (Fig 3, *E*) and an increase in S933 phosphorylation of NF-κB p105 subunit, a precursor of p50, in response to db-cAMP alone and its combination with IL-23 in T_H_17 cells (Fig 3, *E*). The latter is consistent with our previous finding in dendritic cells that PGE_2_-cAMP signaling activates the p50 subunit^54^ and a report that phosphorylation of p105 S933 is PKA dependent.^55^ Therefore we examined the involvement of NF-κB in *Il23r* induction by using *Nfkb1*-deficient mice (p105 knockout [KO]) or CTP-NBD, an NF-κB inhibitor. Interestingly, both genetic deficiency and pharmacologic inhibition of NF-κB suppressed *Il23r* induction in response to db-cAMP, IL-23, and their combination (Fig 3, *F* and *G*).

These results together suggest that PGE_2_-EP2/EP4-cAMP-PKA signaling works together with IL-23 signaling to activate STAT3, CREB1, and NF-κB for induction of *Il23r* expression in T_H_17 cells.

### Gene signature induced by PGE_2_-EP2/EP4-cAMP signaling in CD4^+^ T-cell populations primed with IL-6 and TGF-β1

**Fig 4:**
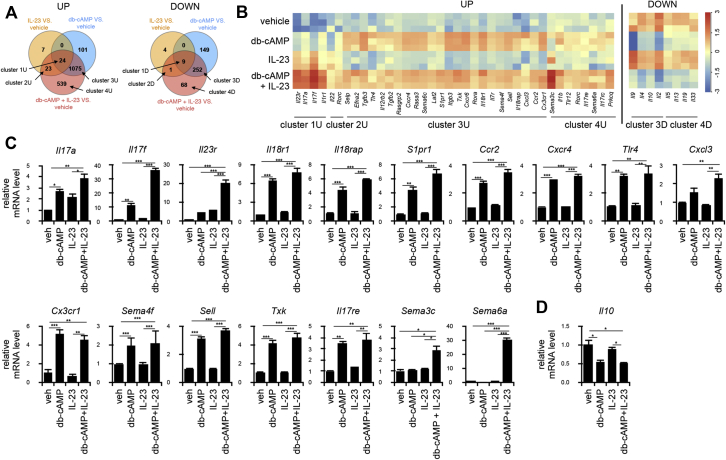
Activation of the COX2-PGE_2_-EP2/EP4-cAMP pathway confers a pathogenic T_H_17 phenotype. **A,** Microarray analysis of gene expression profiles in T_H_17 cells stimulated with db-cAMP, IL-23, or both. Venn diagram analysis of 2-fold upregulated or downregulated genes compared with the vehicle control *(Veh)* on each stimulus (*P* < .05, 1-way ANOVA; n = 3; *left* and *right*, respectively). **B,** Heat map analysis of expression of selected genes from each cluster. **C,** Quantitative RT-PCR analysis of expression of representative genes of T_H_17 signature and immune activation in response to db-cAMP, IL-23, or db-cAMP and IL-23 in combination. **D,** Quantitative RT-PCR analysis of expression of a representative inflammation suppressor gene, *Il10*, in response to db-cAMP, IL-23, or db-cAMP and IL-23 in combination. All *bars* in Fig 4, *C* and *D*, indicate means ± SEMs (n = 3). *P < .05, **P < .01, and ***P < .001.

Because pathogenic T_H_17 cells should express various molecules in addition to IL-23R to exert their pathogenicity, we next examined how PGE_2_-EP2/EP4-cAMP signaling contributes to expression of such pathogenic genes in T_H_17 cells. CD4^+^ T cells were cultured under the T_H_17-skewing conditions with IL-6 and TGF-β1 for 3 days and then incubated with db-cAMP alone, IL-23 alone, or both for 24 hours and subjected to microarray analysis. The numbers of genes upregulated/downregulated greater than 2-fold by each stimulation were examined by using Venn diagrams (Fig 4, *A*), and the genes expressed in each cluster (see Tables E1 to E8 in this article's Online Repository at www.jacionline.org) were subjected to heat map analysis (see Fig E3, *A*, in this article's Online Repository at www.jacionline.org) and gene ontology analysis (see Fig E3, *B*, and Tables E9-E11 in this article's Online Repository at www.jacionline.org). Expression of representative genes in each cluster is shown in the heat map (Fig 4, *B*). Cluster 1U included genes (eg, *Il17a*, *Il17f*, *Il1r1*, and *Il23r*) that were upregulated by db-cAMP, IL-23, or both in combination (Fig 4, *B*, left). Cluster 2U included genes (eg, *Il22*) with expression that was increased by IL-23 alone or its combination with db-cAMP (Fig 4, *B*, left). Cluster 3U encompasses various genes that were upregulated by db-cAMP alone or its combination with IL-23 but not IL-23 alone. They include genes involved in cell migration and adhesion, such as *Ccr2*, *Cxcr4*, *Cx3cr1*, *Ccr6*, *S1pr1*, *Sema4f*, *Sema6c*, *Efna2*, *Sell*, *Selp*, and *Itgb3*; those involved in induction of IFN-γ, such as *Il12rb2*, *Il18r1*, and *Il18rap*; and those involved in cell activation, such as *Tlr4*, *Tgfb3*, *Rasa*, *Rasgrp2*, *Lat2*, *Txk*, and *Rora* (Fig 4, *B*, left). Cluster 4U includes genes, such as *Il1b*, *Il17rc*, *Il17re*, *Prkcq*, *Sema3c*, *Sema6a*, and *Tlr12*, which are upregulated by the combination of IL-23 and db-cAMP only (Fig 4, *B*, left). On the other hand, genes in clusters 3D and 4D were downregulated by db-cAMP and contained *Il10*, *Il2*, *Il4*, *Il5*, *Il13*, and *Il9*, which are known as suppressive factors of inflammation (Fig 4, *B*, right).

Expression of the representative genes was then confirmed by using quantitative RT-PCR analysis. Expression of *Il17a*, *Il17f*, and *Il23r* in cluster 1U; *Il18r1*, *Il18rap*, *S1pr1*, *Ccr2*, *Cxcr4*, *Tlr4*, *Cxcl3*, *Cx3cr1*, *Sema4f*, *Sell*, and *Txk* in cluster 3U; and *Il17re*, *Sema3c*, and *Sema6a* in cluster 4U was upregulated (Fig 4, *C*), and expression of *Il10* in cluster 3D was downregulated by addition of db-cAMP compared with incubation with IL-23 alone (Fig 4, *D*). Thus, signaling through cAMP regulates expression of various genes that are not regulated by IL-23 alone and might confer pathogenic property to T_H_17 cells.

### T cell–intrinsic PGE_2_-EP2/EP4 signaling is critical in IL-23–mediated psoriatic skin inflammation *in vivo*

Accumulating evidence suggests that T_H_17 cells become pathogenic through the IL-23–IL-23R axis and play crucial roles in development of various autoimmune diseases, including psoriasis.^8^, ^56^, ^57^ However, how these T_H_17 cells acquired the pathogenicity *in vivo* and to what extent the microenvironment of diseases contributes to this process remain to be defined. In the IL-23–induced psoriasis mouse model, gene expression of enzymes involved in PGE_2_ biosynthesis, including *Ptgs2* encoding COX2, *Ptges* encoding membrane-associated PGE synthase (mPGES) 1, and *Ptges2* encoding mPGES2, were all upregulated by IL-23 administration into the skin (see Fig E4, *A*, in this article's Online Repository at www.jacionline.org), which is consistent with the clinical observation that local PGE_2_ levels are increased in blister fluids from human psoriatic skin.^58^ Therefore, we hypothesized that IL-23 possibly activates PGE_2_–EP2/EP4 signaling, which can contribute to psoriasis pathogenesis.

**Fig 5:**
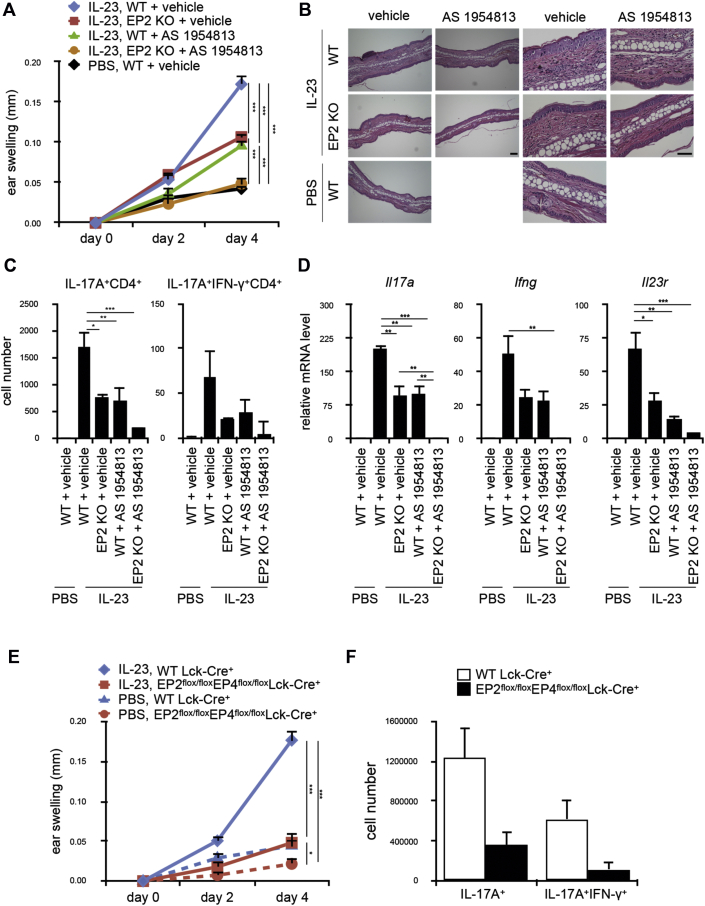
PGE_2_-EP2/EP4 signaling in T cells is required for IL-23–driven psoriatic skin inflammation. **A-D,** Ear swelling (n = 16-17; Fig 5, *A*), representative hematoxylin and eosin staining of the histologic section of the ear (n = 3-4; Fig 5, *B*), number of IL-17A^+^ and IL-17A^+^IFN-γ^+^CD4^+^ T cells of the ear (Fig 5, *C*), and gene expression of *Il17a*, *Ifng*, and *Il23r* in whole ear tissue (Fig 5, *D*) of WT or EP2 KO mice subcutaneously injected with IL-23 or PBS into the ear daily. An EP4 antagonist (AS1954813, 100 mg/kg) or vehicle was orally administered twice a day to the indicated mice. *Bars* in Fig 5, *B* = 50 μm. Representative quantification results of the cell number in each population from 4 independent fluorescence-activated cell sorting experiments are shown in Fig 5, *C* (n = 3). Gene expression was indicated as fold change compared with PBS-injected ears in Fig 5, *D* (n = 3). **E** and **F,** EP2^flox/flox^EP4^flox/flox^Lck-Cre^+^ mice and control WT Lck-Cre^+^ mice were subjected to an IL-23–induced psoriasis model, and ear swelling (n = 11 and 7, respectively; Fig 5, *E*) and numbers of IL-17A^+^ and IL-17A^+^IFN-γ^+^CD4^+^ T cells in the ear (n = 7 and 3, respectively; Fig 5, *F*) were analyzed. All *bars* indicate means ± SEMs. *P < .05, **P < .01, and ***P < .001.

To test this hypothesis, we injected IL-23 into the skin of wild-type (WT) C57BL/6N mice, as well as EP2 KO mice,^46^ with or without administration with the EP4 antagonist AS1954813^49^ and assessed skin inflammation based on ear thickness and histology. EP2 deficiency or EP4 antagonism alone reduced IL-23–induced ear swelling by half and attenuated edema and cell infiltration and, when combined, led to nearly complete suppression of IL-23–dependent skin inflammation (Fig 5, *A* and *B*). Blockade of EP2, EP4, or both caused no alteration in a PBS-injected control ear (see Fig E3, *B*).

To examine at which step of inflammation EP2 deficiency and EP4 antagonism exert their effects and whether it is related to generation of pathogenic T_H_17 cells, we digested ear tissues and analyzed CD4^+^ T-cell populations in the skin by using flow cytometry. Although there were few numbers of cells producing IL-17A or IFN-γ in PBS-injected control ears, significant accumulations of the IL-17A^+^ and IL-17A^+^IFN-γ^+^CD4^+^ T-cell populations were observed in the IL-23–injected ear, as observed in psoriatic dermis of patients with psoriasis.^59^ The IL-17A^+^IFN-γ^+^CD4^+^ T-cell population is a suggested population of pathogenic T_H_17 cells.^60^ This CD4^+^ T-cell population was shown to arise in an IL-23–dependent manner from adoptively transferred T cells in transfer colitis^26^ and might reflect the T_H_17 to T_H_1 reprogramming at inflammatory sites as shown for antigen-specific T_H_17 cells transferred to NOD mice.^22^ This accumulation was significantly reduced by blockade of either EP2 or EP4 alone and nearly completely suppressed by blockade of both EP2 and EP4 (Fig 5, *C*, and see Fig E4, *C-E*). Consistently, expression of *Il17a* and *Ifng* that was upregulated in the IL-23–injected ear was also reduced to negligible levels by combined EP2 and EP4 blockade (Fig 5, *D*, left and middle). Notably, EP2 and EP4 blockade also markedly inhibited enhanced expression of *Il23r* by IL-23 injection (Fig 5, *D*, right). These findings together indicate that EP2/EP4 signaling is indeed involved in generation of pathogenic T_H_17 cells and elicitation of inflammation in this model.

We then investigated whether T cell–intrinsic EP2/EP4 signaling is responsible for these IL-23–induced phenotypes. To this end, we used EP2^flox/flox^ mice^47^ and EP4^flox/flox^ mice^48^ and generated EP2^flox/flox^ EP4^flox/flox^Lck-Cre^+^ mice. EP2^flox/flox^EP4^flox/flox^Lck-Cre^+^ mice showed no significant differences in numbers of total cells, B cells, T cells, CD4 T cells, CD8 T cells, T_H_1 cells, T_H_17 cells, and regulatory T cells in the thymus, spleen, lymph node, and peripheral blood compared with control WT Lck-Cre^+^ mice (see Fig E5, *A*, in this article's Online Repository at www.jacionline.org). However, deficiency of both EP2 and EP4 selectively in T cells prevented accumulation of T_H_17 cells in the ear and almost completely attenuated IL-23–induced skin inflammation (Fig 5, *E* and *F*). Therefore these results together suggest that the T cell–intrinsic PGE_2_-EP2/EP4 signaling is critical for generation of pathogenic T_H_17 cells in a psoriasis model.

We also performed an imiquimod-induced psoriasis model,^8^ in which we applied imiquimod to the ears of WT or EP2 KO mice with or without EP4 antagonist for 6 days (see Fig E6, *A*, in this article's Online Repository at www.jacionline.org). We found that ear swelling was also reduced significantly by EP2 deficiency and EP4 antagonism and additively in combination, which was similar to the results in an IL-23–induced psoriasis model.

Given the above findings, we next examined the effects of COX inhibitors on skin inflammation in an IL-23–induced psoriasis model (see Fig E6, *B* and *C*). Treatment with indomethacin and SC-236 significantly suppressed the IL-23–induced ear swelling with concomitant suppression of IL-17A^+^ and IL-17A^+^IFN-γ^+^ cells in the skin (see Fig E6, *B* and *C*). These findings together suggest that COX inhibitors are as potent as EP2 and EP4 antagonists in suppressing skin inflammation, at least in this model.

### PGE_2_ signaling positively correlates with the IL-23/T_H_17 pathway in human psoriatic skin biopsy specimens

**Fig 6:**
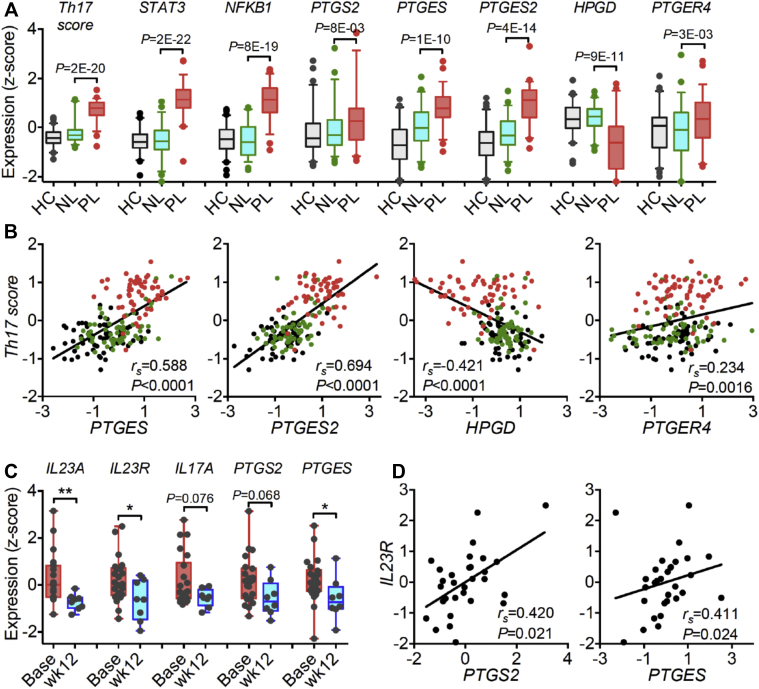
PGE_2_ signaling positively correlates with the IL-23/T_H_17 pathway in human psoriatic skin biopsy specimens. **A,** Expression profiles of genes related to PGE_2_ signaling and T_H_17 signature genes in human nonlesional *(NL)* or lesional *(PL)* skin biopsy specimens from patients with psoriasis (n = 58) and skin samples from healthy control subjects (*HC*; n = 64). The *z* score transformed values of the microarray gene expression data set GSE13355 were used. The T_H_17 score was generated based on the average expression level of *IL23A*, *IL12B*, *IL23R*, *IL17A*, *IL17F*, and *IL22* genes. **B,** Correlations of *PTGES*, *PTGES2*, *HPGD*, and *PTGER4* gene expression versus those of the T_H_17 score. *Black*, *green*, and *red dots* indicate healthy control, nonlesional, and lesional psoriatic biopsy specimens, respectively. **C,** Expression profiles of genes related to PGE_2_ synthases and T_H_17 signature genes in human lesional skin biopsy specimens from patients with moderate-to-severe psoriasis before (*Baseline*, n = 22) or 12 weeks after treatment with the IL-23–specific mAb guselkumab (n = 8). The *z* score transformed values of the microarray gene expression data set GSE51440 were used. **D,** Correlations of gene expression of *PTGS2* and *PTGES* versus that of *IL23R*. *P* values were calculated by using nonparametric Wilcoxon-Mann-Whitney tests (Fig 6, *A* and *C*) or the nonparametric Spearman correlation test (Fig 6, *B* and *D*).

Finally, to extrapolate our findings in mice to human subjects, we analyzed a public microarray data set on gene expression profiles in skin biopsy specimens from patients with psoriasis and healthy control subjects,^61^ with a particular interest in correlation of PGE_2_ signaling and the IL-23/T_H_17 pathway. As expected, psoriatic lesional skin overexpressed T_H_17 signature genes (including *IL23A*, *IL12B*, *IL23R*, *IL17A*, *IL17F*, and *IL22*), *STAT3*, and *NFKB1* (encoding NF-κB p105; Fig 6, *A*). Moreover, psoriatic lesional skin overexpressed enzymes in PGE_2_ biosynthesis (eg, *PTGS2*, *PTGES*, and *PTGES2* and the EP4 receptor *PTGER4*) but underexpressed the PGE_2_ degrading enzyme 15-PGDH (encoded by *HPGD*; Fig 6, *A*). Interestingly, expression of T_H_17 signature genes correlated positively with those involved in PGE_2_ biosynthesis (eg, *PTGES* and *PTGES2*) and receptor (eg, *PTGER4*) but correlated negatively with *HPGD* (Fig 6, *B*). In addition, the clinically effective anti–IL-23 therapy^62^ downregulated gene expression of not only the IL-23/IL-17 pathway (eg, *IL23A*, *IL23R*, and *IL17A*) but also expression of genes involved in PGE synthesis like *PTGES* (Fig 6, *C* and *D*). These findings support a potential crosstalk between the PGE_2_ and IL-23/IL-17 pathways also in human psoriatic skin inflammation.

## Discussion

IL-23–IL-23R signaling plays a critical role in generation of pathogenic T_H_17 cells in autoimmunity.^5^, ^6^, ^7^, ^8^, ^9^ However, there remain several issues to be solved on this action: How does this signaling get promoted. What transcriptional mechanisms other than STAT3 are involved? What, along with IL-23 signaling, makes T_H_17 cells pathogenic? Does such a mechanism operate *in vivo* and, if so, how much? How relevant are the findings obtained in mice to human subjects? Given the previously reported action of PGE_2_ on T_H_17 expansion,^43^, ^44^, ^45^ we focused here on PGE_2_ action in T_H_17 pathogenicity to address these issues.

We first found that PGE_2_ synergizes with IL-23 and enhances *Il23r* expression through EP2 and EP4, a finding consistent with findings in human T_H_17 cells.^44^ We then found that IL-23 stimulation induces PGE_2_ production in T_H_17 cells and that IL-23–induced *Il23r* expression was attenuated by treatment of cells with indomethacin or EP2/EP4 antagonists. Thus, these results suggest a previously unsuspected intrinsic amplification mechanism mediated by PGE_2_-EP2/EP4 signaling in T_H_17 cells that helps trigger the initial IL-23 response in premature T_H_17 cells.

We further analyzed the transcriptional mechanisms underlying the synergistic action of IL-23 and PGE_2_ and found that this action is mediated by not only STAT3 but also CREB1 and NF-κB. Involvement of CREB1 is analogous to that in the PGE_2_-EP2/EP4–mediated *Il12rb2* induction during T_H_1 cell differentiation^42^ and might be consistent with the findings by Hernandez et al^63^ showing that the CREB1/CRTC2 pathway regulates expression of IL-17A and IL-17F and that T_H_17 differentiation is defective in CRTC2 mutant mice. IL-23R and IL-12Rβ2 make a pair with the same molecule, IL-12Rβ1, to form IL-23R and IL-12R, respectively. It is interesting that the same pathway regulates expression of these 2 genes. We have also used T cells from p105 NF-κB1–deficient mice and CTP-NBD and unraveled the involvement of NF-κB in the IL-23/cAMP–induced *Il23r* expression in T_H_17 cells. Consistent with these findings, we previously found that PGE_2_, through EP2 or EP4, activates NF-κB1 containing NF-κB in various types of cells, including macrophages and dendritic cells, and induces expression of inflammation-related genes, including COX2, which then produces PGE_2_ and amplifies this process.^47^, ^54^, ^64^ Thus our present findings further extend the importance of this COX2–PGE_2_–EP2/EP4–NF-κB loop to generation of T_H_17 cell pathogenicity.

On the other hand, Boniface et al^44^ suggested that PGE_2_-induced enhancement of *Il23R* expression in human T_H_17 cells was mediated by the IL-1β–IL-1 receptor pathway. This is also a possibility in mice because upregulated expression of *Il1r1* and *Il1b* was detected in clusters 1U and 4U by using our microarray analysis (Fig 4, *B*, left). However, we assume that this mechanism is not critical in our experiment because addition of anti–IL-1β antibody to the medium did not reduce *Il23r* induction (see Fig E7 in this article's Online Repository at www.jacionline.org).

In addition to *Il23r*, our microarray analysis has revealed that stimulation of EP2/EP4 signaling together with IL-23 facilitates expression of a variety of pathogenic T_H_17 signature genes (ie, *Il17a*, *Il17f*, *Il18r1*, and *Tgfb3*). Interestingly, PGE_2_-EP2/EP4 signaling also upregulated expression of various genes related to chemotaxis and migration, such as *S1pr1*, *Ccr2*, *Cxcl3*, *Cx3cr1*, *Cxcr4*, *Sema4f*, *Sell*, *Sema3c*, and *Sema6a* (Fig 4, *B*, left). These results suggest that PGE_2_-EP2/EP4 signaling can contribute to migration, infiltration, and accumulation of T_H_17 cells into the inflammatory lesion. On the other hand, the addition of db-cAMP downregulated expression of *Il10*, *Il2*, *Il4*, and *Il9*, which are known as suppressive factors for T_H_17 cells. Although some of these results, such as IL-17A, are consistent with the previous findings in human T_H_17 cells,^44^ our study did not detect induction of IFN-γ and T-bet in cultured T_H_17 cells, which might reflect the stages of T_H_17 cells examined in each study.^20^, ^24^, ^65^ It should also be noted that our analysis was carried out on the whole CD4^+^ T-cell population pretreated with IL-6 and TGF-β1 and stimulated with each stimulus, in which IL-17A^+^ cells comprised about 10% of cells. Therefore single-cell RNA sequencing analysis is desired to establish gene expression signatures specific to T_H_17 cells matured with each stimulus.

Nonetheless, the most important point in our study was that the EP2/EP4 signaling in T_H_17 cells identified here is critical in eliciting their pathogenicity *in vivo* in immune inflammation. We tested this issue in an IL-23–induced mouse psoriasis model. Intriguingly, not only the systemic inhibition of EP2/EP4 signaling with the EP4 antagonist in EP2 KO mice but also selective loss of EP2 and EP4 in T cells almost completely suppressed inflammation induced by IL-23. This was accompanied by suppression of accumulation of IL-17A^+^ and IL-17A^+^IFN-γ^+^ T cells and suppression of expression of *Il17a*, *Ifng*, and *Il23r* genes in the lesion. These results suggest that PGE_2_-EP2/EP4 signaling functions is critical to generation of pathogenic T_H_17 cells induced by IL-23 *in situ*. Of those T_H_17 cells, antigen-specific T_H_17 cells were shown to be involved specifically in the pathogenesis of mouse models of autoimmune inflammation, including experimental autoimmune encephalomyelitis,^66^ type 1 diabetes,^22^ and psoriasis.^67^ Quite recently, it was also reported that mPGES1 is involved in generation of antigen-specific T_H_17 cells by regulating PGE_2_ production in a T-cell autocrine and paracrine manner.^68^ Our present findings combined with these findings suggest that PGE_2_ plays an important role in psoriasis through regulation of antigen-specific pathogenic T_H_17 cells.

The present study also showed that EP2 deficiency and EP4 antagonism significantly suppressed psoriatic inflammation in an imiquimod model. Notably, however, the combined EP2 deficiency and EP4 antagonism did not completely suppress ear swelling in this model, possibly because there is the IL-17–independent component in skin inflammation in this model.^8^

In this study we also tested the effect of COX inhibitors in an IL-23–induced psoriasis model and found that COX inhibitors are as potent as EP2 and EP4 antagonists in suppressing psoriasis-like skin inflammation in this model. The question is whether COX inhibitors are beneficial in T_H_17-driven human autoimmune diseases. COX inhibitors, particularly celecoxib, are used for treatment of the early stage of rheumatoid arthritis and in patients with mild psoriatic arthritis.^69^ In these cases COX inhibitors produce good symptomatic relief. Although this effect is ascribed to their analgesic and general anti-inflammatory actions, our study suggests that it might be derived at least in part from their suppressive action on T_H_17-mediated pathology, a possibility that should be tested in the future.

On the other hand, COX inhibitors have less appreciable therapeutic benefits in patients with established psoriasis and advanced rheumatoid arthritis in human patients. There are several plausible reasons. A PG-mediated process might be critical in triggering pathogenic T_H_17 cell generation but not so in advanced stage of diseases that might be regulated dominantly by established T_H_17 cells. Another reason might be the fact that PGs cause immune inflammation not by acting alone but by working with cytokines and boosting and modifying their actions. Therefore, COX inhibitors might exert therapeutic benefits more effectively when combined with anticytokine drugs and lessen the dose of the latter. Finally, COX inhibitors might divert arachidonate metabolism to leukotriene. Recent studies suggest that leukotrienes facilitate maturation and migration of T_H_17 cells.^70^, ^71^ Further studies need to be conducted to unravel these issues.

Another topic to be discussed on use of PGE_2_ in patients with psoriasis is its facilitative action in UV irradiation therapy, which at a glance contradicts our present findings on the facilitative action of PGE_2_ on T_H_17 pathogenicity. UVB irradiation is an effective therapeutic treatment of psoriasis by inducing immunosuppression.^72^ We previously showed that UVB induces PGE_2_ in the epidermis and PGE_2_-EP4 signaling mediates systemic immunosuppression through upregulation of receptor activator of NF-κB ligand in keratinocytes and inducing regulatory T cells.^73^ Thus the PGE_2_-EP4 signaling in this case facilitates immunosuppression and not immune activation. One point is that UVB does not penetrate to the dermis and the events it causes are within the epidermis, whereas IL-23–induced inflammatory events occur in the dermis. Another point is a difference in context, UVB irradiation in the UV therapy and IL-23 in psoriatic inflammation. PGE_2_ alone does not induce either effect but functions directionally dependent on the context.

Finally, we examined the relevance of our findings to human disease by analyzing biopsy samples from patients with psoriasis. Psoriatic lesional skin overexpressed not only T_H_17 signature genes, including *IL23A*, *IL12B*, *IL23R*, *IL17A*, *IL17F*, *IL22*, *STAT3*, and *NFKB1*, but also those involved in PGE_2_ biosynthesis and function, such as *PTGS2*, *PTGES*, *PTGES2*, and *PTGER4*. Expression of T_H_17 signature genes shows positive correlation with *PTGES*, *PTGES2*, and *PTGER4* and negative correlation with *HPGD* and the anti–IL-23 therapy downregulated expression of not only genes in the IL-23/IL-17 pathway (eg, *IL23A*, *IL23R*, and *IL17A*) but also those in PGE_2_ synthesis, suggesting that these 2 are functionally linked. These findings together with the finding by Kofler et al^74^ that EP2 is expressed in T_H_17 cells from patients with multiple sclerosis and that forced expression of EP2 in healthy T_H_17 cells triggers expression of pathogenic genes indicate that T cell–intrinsic EP2/EP4 signaling is critical in IL-23–driven T_H_17 cell pathogenesis also in human subjects and support a view that the combined inhibition of EP2 and EP4 is of value in therapeutic intervention of various T_H_17-mediated diseases.

**Key messages**

• IL-23 triggers T cell–intrinsic PGE_2_-EP2/EP4 signaling that is critical in T_H_17 cell–mediated immune pathogenesis.
• PGE_2_-EP2/EP4 signaling functions synergistically with IL-23 and not only amplifies *Il23r* expression but also induces a unique pathogenic gene expression signature by activating STAT3, CREB1, and NF-κB.
• This PGE_2_ signaling can be a therapeutic target of T_H_17 cell–mediated diseases because combined blockade of EP2 and EP4 suppresses IL-23–induced pathogenic T_H_17 cell generation and consequent psoriatic skin inflammation.

## Supplementary data

Online Repository text

Legends for Figures E1-E7 and Tables E1-E11

Table E1

Table E2

Table E3

Table E4

Table E5

Table E6

Table E7

Table E8

Table E9

Table E10

Table E11

Fig E1

Fig E2

Fig E3

Fig E4

Fig E5

Fig E6

Fig E7
