# Supplementary material for: T cell–intrinsic prostaglandin E2-EP2/EP4 signaling is critical in pathogenic TH17 cell–driven inflammation
Source: J Allergy Clin Immunol. 2019 Feb;143(2):631–43. doi: 10.1016/j.jaci.2018.05.036 (PMC6354914; doi:10.1016/j.jaci.2018.05.036)
Supplement: Online Repository text [file mmc1.docx]

**SUPPLEMENTARY METHODS**

**Purification of CD4^+^ T cells and differentiation into Th17 cells**

Spleen was dissected from 6-10 week-old female C57BL/6N mice and cells were dissociated and collected. CD4^+^ T cells were purified from spleen cells by magnetic activated-cell sorting (MACS) using anti-CD4 microbeads (L3T4) (#130-049-201, Miltenyi) on auto-MACS (Miltenyi). The purity of CD4^+^ T cells was ~98 % (n=3) as assessed by FACS (FACS LSR Fortessa, BD Bioscience, San Jose, CA). Purified CD4^+^ T cells were differentiated into Th17 cells by the combination of TGF-β1 (1 ng/ml, #240-B-002, R&D systems, Minneapolis, MN) and IL-6 (20 ng/ml, R&D systems) in the presence of 5 μg/ml of anti-CD3 antibody (#14-0031-86, eBioscience, San Diego, CA) and 2.5 μg/ml of anti-CD28 antibody (#14-0281-86, eBioscience, San Diego, CA) in RPMI-1640 medium containing 10 % fetal bovine serum (FBS) for 4 days. Differentiated cells were then collected, washed and again plated for experiments with TCR stimulation. Experimental condition of each experiment is shown in the **Results** or the **Figure Legends unless specified otherwise**.

**Reagents**

Agonists selective to each PGE_2_ receptor subtype, EP1, EP2, EP3 and EP4 (ONO-DI-004, ONO-AE1-259, ONO-AE-248 and ONO-AE1-329 respectively) and an EP4 antagonist, ONO-AE3-208,^E1^ were kindly provided by Ono Pharmaceutical Co., Osaka, Japan. An EP2 antagonist, PF-04418948, was synthesized according to the previous report.^E2^ An EP4 antagonist, AS1954813, was kindly provided by Astellas Pharmaceutical Co. (Tsukuba, Japan). PGE_2_, SC-560 and SC-236 were purchased from Cayman Chemical, Ann Arbor, MI. Dibutyryl cAMP (db-cAMP), forskolin, the N6-Bnz-cAMP, the 8-pCTP-2’-O-Me-cAMP, indomethacin and KG-501 were purchased from Sigma, St. Louis, MO. STAT3 inhibitor VII, Src Kinase Inhibitor-I, H-89, and CPT-NBD peptides were purchased from Calbiochem, San Diego, CA. Mouse IL-1β/IL-1F2 Antibody (AF-401-SP) was purchased from R&D systems.

**Quantitative reverse transcription polymerase chain reaction (qRT-PCR)**

RNA purification and reverse transcription were performed by the RNeasy Mini Kit (Qiagen GmbH, Hilden, Germany) and the High-capacity cDNA Reverse Transcription Kit (ABI biosystems, Grand Island, NY) according to manufacturers’ instructions. cDNA, primers and FastStart DNA MasterPLUS SYBR Green (Takara, Shiga, Japan) were then mixed in 96-well PCR plate, and quantitative PCR was performed using CFX96 Real-Time System (Biorad). The following primers were used in this study;

*Gapdh*: forward 5′-TGAACGGGAAGCTCAC-3′and reverse 5′-TCCACCACCCTGTTGC-3′

*Il17a*: forward 5′-TGTGAAGGTCAACCTCAAAGTC-3′ and reverse 5′-GAGGGATATCTATCAGGGTCTTCA-3′

*Il23r*: forward 5′-CCAAGTATATTGTGCATGTGAAGA-3′ and reverse 5′-AGCTTGAGGCAAGATATTGTTGT-3′

*Ptgs2*: forward 5′- TCGCAGGAAGGGGATGTTGT -3’ and reverse 5’- CTGAAGCCCACCCCAAACAC -3′

*Creb1*: forward 5′-CCAAACTAGCAGTGGGCAGT-3′ and reverse 5′-CCCCATCCGTACCATTGTT-3′

*Il17f*: forward 5′-GGAAGACAGCACCATGAAC-3′ and reverse 5′-TGGACAATGGGCTTGACAG-3′

*Il18r1*: forward 5′-GTTGAGATGGAGGATGAGGG-3′ and reverse 5′-GACAGAAAACACGCAGGAG-3′

*Il18rap*: forward 5′-AGCCTTTAACTCTCCCCTG-3′ and reverse 5′-ACACCACCTCTTCCTTCTTC-3′

*S1pr1*: forward 5′-CATTCTCATCTGCTGCTTCATC-3′ and reverse 5′-CCACAAACATACTCCCTTCCC-3′

*Ccr2*: forward 5′-TGAGAAGAAGAGGCACAGG-3′ and reverse 5′-CAACAAAGGCATAAATGACAGG-3′

*Cxcr4*: forward 5′-ATCTGTGACCGCCTTTACCC-3′ and reverse 5′-ATCCTTGCTTGATGACCCCC-3′

*Tlr4*: forward 5′-CTTTCACCTCTGCCTTCAC-3′ and reverse 5′-TACAATTCCACCTGCTGCC-3′

*Cxcl3*: forward 5′-GAACACCCTCAGGCTCAAGG-3′ and reverse 5′-CCACCAACCAAAGAATACACATGG-3′

*Cx3cr1*: forward 5′-ACAAAGAGAAAGGACAACGAG-3′ and reverse 5′-TGATGCGGAAGTAGCAAAAG-3′

*Sema4f*: forward 5′-AAGAAAGGCAAGAAAGAGGAC-3′ and reverse 5′-CACATCAATAACCCCGCAC-3′

*Sell*: forward 5′-TGCCAAGAGACAAACAGAAG-3′ and reverse 5′-CCAGCCAAATGAGAAATGCC-3′

*Txk*: forward 5′-CACCGAAAGACATCTCTTCC-3′ and reverse 5′-ACAACCCCAAACTGACCAC-3′

*Il17re*: forward 5′-ACAACCCCAAACTGACCAC-3′ and reverse 5′-GGGCAGCAAATCAAAGGAG-3′

*Sema3c*: forward 5′-ACAAAGACAGGAGGAAGGAG-3′ and reverse 5′- AGTGGCAATGCAGTGGTAG-3′

*Sema6a*: forward 5′-GCTCACTCTATGTTGCATTCTC-3′ and reverse 5′- ACTTTCCCTTACCCACCCAC-3′

*Il10*: forward 5′-TGGGTGAGAAGCTGAAGACC-3′ and reverse 5′-TTCATGGCCTTGTAGACACC-3′

*Ifng*: forward 5′-ATCTGGAGGAACTGGCAAAA-3’ and reverse 5’-TTCAAGACTTCAAAGAGTCTGAGGTA -3′

Expression level of each gene was normalized to that of *Gapdh* and calculated relative to the expression in vehicle-treated group.

**Measurement of IL-17 and PGE_2_ concentration in culture supernatant of Th17 cells**

IL-17 concentration in culture supernatant of differentiated Th17 cells stimulated with 100 μM db-cAMP, 10 μM FSK or 100 μM IBMX for 3 days was measured by a Mouse IL-17 Quantikine ELISA Kit (M1700, R&D systems) according to the manufacturer’s instruction.

Th17 cells were stimulated with 10 ng/ml IL-23 for 3 days in the absence or presence of 100 μM indomethacin and PGE_2_ concentration in culture supernatant was determined by a Prostaglandin E_2_ ELISA kit - monoclonal (514010, Cayman Chemical, Ann Arbor, MI) according to the manufacturer’s instruction.

**Gene expression of Th17 cells stimulated IL-23 and/or cAMP from microarray analysis**

CD4^+^ T cells were incubated in Th17-skewing condition for 4 days. Differentiated Th17 cells were then stimulated by IL-23, db-cAMP or combination for 24 h. RNA was purified with an RNeasy Mini Kit, amplified and revers transcribed by the High-capacity cDNA Reverse Transcription Kit. cDNA was fragmented and labeled by a Low Input Quick Amp Labeling Kit (Agilent, Santa Clara, CA), and then hybridized to a Gene Expression Large Volume Hybridization Kit (Agilent). Hybridized genes were scanned by Gene chip scanner 3000 system. Data were analyzed by GeneSpring software (Agilent Technology, Santa Clara, CA).

**Flow cytometry**

The medium was removed after each incubation, and cells were re-stimulated with 50 ng/ml phorbol 12-myristate 13-acetate (PMA) (Sigma) and 500 ng/ml ionomycin (Sigma) in the presence of GolgiPlug (BD bioscience) for 4 h, followed by fixation and permeabilization with a fixation/permeabilization solution (Cytofix/Cytoperm, BD Pharmingen). Cells were then stained with anti-mouse CD45.2 antibody (eBioscience), anti-mouse CD4 antibody(BioLegend), anti-mouse IFN-γ antibody (eBioscience) and anti-mouse IL-17A antibody (BioLegend) followed by FACS analysis on LSR Fortessa (BD Bioscience).

**Western blot analysis**

Differentiated Th17 cells were cultured with 10 ng/ml IL-23 for 3 days to induce IL-23R, rested to return STAT3 phosphorylation to the basal level, and then re-stimulated with 100 μM db-cAMP and/or 100 ng/ml IL-23 for 30 min with indicated compounds for indicated time. Total cell lysates were prepared with RIPA buffer (Sigma) containing a phosphatase inhibitor cocktail (PhosSTOP, Roche, Basel, Switzerland) and a proteinase inhibitor cocktail (Complete Protease Inhibitor Cocktail, Roche). Lysates were then subjected to SDS-PAGE (sodium dodecyl sulfate-poly-acrylamide gel electrophoresis) and separated proteins were transferred to a PVDF membrane (Millipore, Darmstadt, Germany). After blocking with an ECL Blocking Agent (GE Healthcare, Piscataway, NJ), membranes were incubated with primary antibodies, followed by incubation with secondary antibodies conjugated with horseradish peroxidase (GE). Signals were detected using an ECL Prime Western Blotting Detection Reagent (GE) on LAS-4000 (GE). Primary antibodies used were; mouse monoclonal anti-α-Tubulin antibody (clone DM1A, #T6199, Sigma), mouse monoclonal anti-GAPDH antibody (clone 6C5, #AM4300, Ambion, Austin, TX), rabbit monoclonal anti-STAT3 antibody (clone 79D7, #4904, Cell Signaling Technology, Danvers, MA), rabbit monoclonal anti-phosphorylated STAT3 antibody (Y705; #9145, Cell Signaling Technology), rabbit monoclonal anti-phosphorylated STAT3 antibody (S727; #9134, Cell Signaling Technology), rabbit monoclonal anti-JAK2 antibody (clone D2E12, #3230, Cell Signaling Technology), rabbit monoclonal anti-phosphorylated JAK2 antibody (Tyr1007/1008; #3771, Cell Signaling Technology), rabbit monoclonal anti-NF-κB p65 antibody (clone D14E12, #8242, Cell Signaling Technology), rabbit monoclonal anti-phosphorylated NF-κB p65 antibody (S536; clone 93H1, #3033, Cell Signaling Technology), rabbit monoclonal anti-p105/p50 antibody (#3035, Cell Signaling Technology), and rabbit monoclonal anti-phosphorylated p105 antibody (S933; #4086, Cell Signaling Technology).

**RNA interference**

siRNA for mouse *Creb1* (5′-UUGAACAACAACUUGGUUGCUGGGC-3′(sense) or 5′-GCCCAGCAACCAAGUUGUUGUUCAA-3′ (antisense) and scrambled control siRNA were obtained from Invitrogen (Stealth RNAi, Carlsbad, CA). Th17 cells differentiated with TGF-β1 (1 ng/ml) and IL-6 (20 ng/ml) were transfected with 500 pmol of each siRNA using an Amaxa P3 Primary Cell 4D-Nucleofector X Kit with the program DN100 on a 4D-Nucleofector (Lonza, Basel, Switzerland) in 100 μl. After transfection for 4 h, cells were washed and stimulated with or without 10 ng/ml IL-23 for 2 days , and then incubated with or without 100 μM db-cAMP for 1 day. Total RNA was prepared and then subjected to qRT-PCR analysis.

**Histology**

The ear tissues from psoriasis models were removed and fixed by 4 % Paraformaldehyde (PFA) for 48h at 4°C. Each ear tissues were embedded in paraffin, sectioned at 5 μm thickness, and then stained with hematoxylin-Eosin.

**Analysis of gene expression of human skin biopsies and mouse IL-23-treated ear from microarray datasets**

Microarray gene expression data of human skin biopsies were retrieved from Gene Expression Omnibus datasets (GSE51440 and GSE13355).^52,53^ Patients information and skin samples have been described previously.^52,53^ In brief, two biopsies were taken from each patient - one from lesional skin of each patient (involved sample) and the other from non-lesional skin (uninvolved sample), taken at least 10 cm away from any active plaque. One biopsy was obtained from each healthy control.^52^ Microarray gene expression data of IL-23-treated ear from mice was retrieved from GSE13335.^53^ Gene expression levels were transformed to z-score values. *P* values were calculated by nonparametric Wilcoxon-Mann-Whitney test, and correlations between expression levels of two genes were calculated by nonparametric Spearman correlation test.

**Statistical Analysis**

Data are shown in mean ± SEM. Statistical comparisons among more than two groups were conducted using One-way ANOVA with Bonferoni test. Statistical comparisons between two groups were conducted using Mann-Whitney test. P values of 0.05 or less were considered significant.

**REFERENCES**

E1. Sugimoto Y, Narumiya S. Prostaglandin E receptors. J Biol Chem 2007;282:11613-11617. <https://doi.org/10.1074/jbc.R600038200>

E2. af Forselles KJ, Root J, Clarke T, Davey D, Aughton K, Dack K, et al. In vitro and in vivo characterization of PF-04418948, a novel, potent and selective prostaglandin EP(2) receptor antagonist. Br J Pharmacol 2011;164:1847-1856. <https://doi.org/10.1111/j.1476-5381.2011.01495.x>
