# Supplementary material for: T cell–intrinsic prostaglandin E2-EP2/EP4 signaling is critical in pathogenic TH17 cell–driven inflammation
Source: J Allergy Clin Immunol. 2019 Feb;143(2):631–43. doi: 10.1016/j.jaci.2018.05.036 (PMC6354914; doi:10.1016/j.jaci.2018.05.036)
Supplement: Legends for Figures E1-E7 and Tables E1-E11 [file mmc2.docx]

**SUPPLEMENTARY FIGURES LEGENDS**

Figure E1. **Effects of COX inhibitors on *Il23r* expression.** (A) Expression of *Il23r* in differentiated Th17 cells treated with IL-23 (10 ng/ml) and/or PGE_2_ (100 nM) in the absence or presence of a COX-1 inhibitor, SC-560 (100 μM) or a COX-2 inhibitor, SC-236 (100 μM) or both for 3 days. (B) Th17 cells were cultured with vehicle, IL-23 (10 ng/ml) and PGE_2_ (100 nM) or IL-23 (10 ng/ml), EP2 agonist (100 nM) and EP4 agonist (100 nM) in the absence or presence of indomethacin (100 μM) for 3 days, and then harvested to analyze for *Il23r* expression by qRT-PCR.

Figure E2. **db-cAMP activates JAK2 and STAT3 in Th17 cells.** (A) Time-course of STAT3 Y705 phosphorylation by vehicle, db-cAMP, IL-23 or db-cAMP and IL-23 in Th17 cells. Th17 cells were treated with IL-23 (10 ng/ml) for 3 days to induce IL-23R expression. The cells were then stimulated with either 100 μM db-cAMP or 100 ng/ml IL-23 or in combination for indicated times. Phosphorylation of STAT3 at Y705 residues and S727 residues under each condition was examined at indicated times by Western blot analysis using total cell lysates and antibodies to each phosphorylation site of STAT3, total STAT3 and α-Tubulin. Representative images are shown (n=2). (B) Involvement of Src family kinase in cAMP-induced JAK2 Y1007/Y1008 phosphorylation. Th17 cells were stimulated with 100 μM db-cAMP for 10 min in the presence of a Src inhibitor, Src Kinase Inhibitor I (10 μM), and subjected to Western blot analysis. Data was from a single experiment.

Figure E3. **Heat-map and gene ontology analysis of genes in each cluster.** (A) Gene expression profiles in Th17 cells stimulated with db-cAMP and/or IL-23 followed by microarray analysis. Heat-map analysis of expression of genes 2-folds up- or down-regulated upon each stimulus compared to the vehicle control (One-way ANOVA p<0.05, n=3). (B) Gene ontology analysis of each clusters by GeneSpring.

Figure E4. **Involvement of PGE_2_ signaling in psoriasis-like model.** (A) Gene expression of PGE_2_ synthases in ear skin from naïve WT mice or psoriasis-like skin lesions from mice administrated with IL-23 by intradermal injection in the dorsum (n=5 each). Gene expression was retrieved from a public dataset GSE13335. (B and C) Genetic loss of *Ptger2* (EP2 KO) or pharmacological EP4 antagonism alone does not cause alteration in the ear. Psoriasis-like model in WT and EP2 KO mice were established as described in Figure 5A. Ear swelling was measured every 2 days (B) (n=14, 10, 8, and 10 in vehicle-treated mice, EP2 KO mice, AS1954813-treated WT mice, AS1954813-treated EP2 KO mice, respectively) and ear skins were subjected to FACS analysis on day 4 (C). (D and E) Suppression of IL-17A^+^ and IL-17A^+^IFN-γ^+^ CD4^+^ T cell accumulation by EP2 KO and EP4 antagonist. WT and EP2 KO mice were administered either vehicle or AS1954813, and subcutaneously injected IL-23. CD4^+^ T cells were purified from the ear of each group on day 4 and examined by FACS for IL-17A and IFN-γ. Representative data from 4 independent experiments are shown. All bars indicate mean ± SEM. *, p<0.05, **, p<0.01, ***, p<0.001.

Figure E5. **FACS analysis of cell populations of EP2^fl/fl^EP4^fl/fl^Lck-Cre^+^ mice or WT Lck-Cre^+^ mice.** (A) Cell population of EP2^fl/fl^EP4^fl/fl^Lck-Cre^+^ mice and control WT Lck-Cre^+^ mice. The numbers of B cell, T cell, CD4 T cell, CD8 T cell, Th1 cell, Th17 cell and Treg cell isolated from thymus, spleen, lymph node, and peripheral blood were analyzed by FACS. (n=3-4)

Figure E6. **Involvement of PGE_2_ signaling in IMQ-induced psoriasis-like model and the effect of COX inhibitors on IL-23-induced psoriasis model.** (A) Control WT mice and *Ptger2*-deficient (EP2 KO) mice were subjected to imiquimod (IMQ)-induced psoriasis model and administered either vehicle or AS1954813, 100 mg/kg, as described in Figure 5A. Ear swelling was measured every 2 days (A) (n=14, 10, 8, and 10 in vehicle-treated mice, EP2 KO mice, AS1954813-treated WT mice, AS1954813-treated EP2 KO mice, respectively). (B-D) Female WT mice were subjected to IL23-induced psoriasis model and administered either vehicle, SC-236 (10mg/kg) or indomethacin (4 mg/kg). Ear swelling was measured every 2 days (B) (n=4, respectively) and mice were sacrificed and subjected to FACS analysis at day 4 (C and D).

Figure E7. **IL-1β-IL1 receptor signaling was not involved in *Il23r* expression by Th17 cells.** Expression of *Il23r* gene in differentiated Th17 cells stimulated with db-cAMP, IL-23 or db-cAMP and IL-23 in combination in the absence or presence of various concentrations of neutralization antibody for IL-1β for 3 days was analyzed by qRT-PCR (n=3). All bars indicate mean ± SEM.

Table E 1. **List of genes in Cluster 1U.**

Table E 2. **List of genes in Cluster 2U.**

Table E 3. **List of genes in Cluster 3U.**

Table E 4. **List of genes in Cluster 4U.**

Table E 5. **List of genes in Cluster 1D.**

Table E 6. **List of genes in Cluster 2D.**

Table E 7. **List of genes in Cluster 3D.**

Table E 8. **List of genes in Cluster 4D.**

Table E 9. **List of gene ontology from Cluster 1U.**

Table E 10. **List of gene ontology from Cluster 2U.**

Table E 11. **List of gene ontology from Cluster 3U.**
