# Supplementary material for: T cell–intrinsic prostaglandin E2-EP2/EP4 signaling is critical in pathogenic TH17 cell–driven inflammation
Source: J Allergy Clin Immunol. 2019 Feb;143(2):631–43. doi: 10.1016/j.jaci.2018.05.036 (PMC6354914; doi:10.1016/j.jaci.2018.05.036)
Supplement: Table E1 [file mmc3.docx]

| ProbeName | GeneSymbol |
| --- | --- |
| A_51_P193686 | 1700012B09Rik |
| A_55_P2059010 | Rbp1 |
| A_66_P116173 | Il23r |
| A_30_P01019068 |  |
| A_55_P2424921 | 1300014J16Rik |
| A_30_P01030266 |  |
| A_30_P01021272 |  |
| A_55_P2059765 | Foxf1 |
| A_55_P2300071 | 4833412C15Rik |
| A_30_P01019394 |  |
| A_55_P2156697 | Il17a |
| A_30_P01033385 |  |
| A_30_P01020711 |  |
| A_51_P519301 | Il17f |
| A_30_P01023418 |  |
| A_52_P536494 | Mycn |
| A_55_P2139942 | Calca |
| A_51_P415306 | 4930563D23Rik |
| A_52_P374653 |  |
| A_51_P435844 | Nr2e3 |
| A_30_P01028775 |  |
| A_51_P271503 | Il1r1 |
| A_55_P1995647 | Rsph4a |
| A_52_P593465 | Arap2 |
