# Supplementary material for: T cell–intrinsic prostaglandin E2-EP2/EP4 signaling is critical in pathogenic TH17 cell–driven inflammation
Source: J Allergy Clin Immunol. 2019 Feb;143(2):631–43. doi: 10.1016/j.jaci.2018.05.036 (PMC6354914; doi:10.1016/j.jaci.2018.05.036)
Supplement: Table E2 [file mmc4.docx]

| ProbeName | GeneSymbol |
| --- | --- |
| A_55_P1962523 | Zic2 |
| A_52_P650387 | Ccnjl |
| A_51_P146970 | Dmrt2 |
| A_51_P167527 | Lum |
| A_51_P290387 | Sval1 |
| A_55_P2199202 | Il22 |
| A_55_P1985850 | Timp1 |
| A_55_P2089957 | Myo3a |
| A_30_P01025708 |  |
| A_55_P1986647 | Podxl2 |
| A_30_P01032975 |  |
| A_30_P01023119 |  |
| A_55_P1962400 | Il1rn |
| A_55_P1994942 | Rorc |
| A_55_P1980771 | Olfr536 |
| A_55_P2052016 | Crispld2 |
| A_55_P2055597 | Enpp2 |
| A_52_P415155 | Wnt6 |
| A_55_P2238965 | Cwh43 |
| A_30_P01029390 |  |
| A_55_P1998737 | 9130015A21Rik |
| A_51_P212754 | Tgfbi |
| A_55_P1991465 |  |
