# Supplementary material for: T cell–intrinsic prostaglandin E2-EP2/EP4 signaling is critical in pathogenic TH17 cell–driven inflammation
Source: J Allergy Clin Immunol. 2019 Feb;143(2):631–43. doi: 10.1016/j.jaci.2018.05.036 (PMC6354914; doi:10.1016/j.jaci.2018.05.036)
Supplement: Table E3 [file mmc5.docx]

| ProbeName | GeneSymbol |
| --- | --- |
| A_55_P2153545 |  |
| A_55_P1955078 | Igflr1 |
| ERCC-00018_67 |  |
| A_51_P483576 |  |
| A_55_P2372228 | A430104N18Rik |
| A_55_P2040490 |  |
| A_55_P2130104 | Slc52a3 |
| A_55_P2149791 |  |
| A_55_P2224830 | D230044B12Rik |
| A_30_P01021682 |  |
| A_55_P2011341 |  |
| A_55_P2091496 | Dppa3 |
| A_55_P1977558 | Dip2b |
| A_30_P01027915 |  |
| A_55_P2149500 | Kifc2 |
| A_55_P2175502 | M1ap |
| A_51_P288916 | Tmtc2 |
| A_52_P21550 | Gcnt1 |
| A_52_P221776 | Kif12 |
| A_30_P01028727 |  |
| A_30_P01032107 |  |
| A_55_P1957865 |  |
| A_51_P211854 | Selp |
| A_55_P2041514 | 4930550L24Rik |
| A_55_P2141093 | Eya2 |
| A_30_P01031172 |  |
| A_55_P2180176 | Ms4a6b |
| A_55_P2004511 | Cd300lf |
| A_55_P2049687 | Efna2 |
| A_55_P2322555 | 5930433N17Rik |
| A_30_P01026837 |  |
| A_30_P01030563 |  |
| A_66_P130030 | LOC102642739 |
| A_30_P01027181 |  |
| A_30_P01023491 |  |
| A_30_P01026677 |  |
| A_52_P16232 | Gabbr1 |
| A_30_P01022661 |  |
| A_30_P01022155 |  |
| A_30_P01021193 |  |
| A_52_P214408 | Gm5148 |
| A_51_P386670 | Dse |
| A_55_P2156598 | Gm9869 |
| A_55_P1993483 |  |
| A_66_P101393 | A530001N23Rik |
| A_51_P132013 | Cysltr2 |
| A_51_P407657 |  |
| A_66_P102202 | Gm648 |
| A_30_P01024802 |  |
| A_52_P244193 | Cd24a |
| A_51_P108459 | Gpr65 |
| A_55_P2039354 |  |
| A_55_P2404823 | 1700095A21Rik |
| A_55_P2101944 |  |
| A_30_P01028859 |  |
| A_51_P124748 | Tgfb3 |
| A_30_P01018979 |  |
| A_55_P1996434 |  |
| A_55_P2129207 | Tmem71 |
| A_66_P124806 | Tlr4 |
| A_30_P01019442 |  |
| A_55_P2148873 | Cat |
| A_30_P01031480 |  |
| A_55_P2344598 | E330037I15Rik |
| A_30_P01019859 |  |
| A_51_P505823 | Endod1 |
| A_30_P01025027 |  |
| A_55_P2397599 | B930025B16Rik |
| A_30_P01024408 |  |
| A_30_P01019299 |  |
| A_30_P01025650 |  |
| A_55_P2409088 | BB163080 |
| A_55_P2006808 | Ntrk3 |
| A_30_P01021307 |  |
| A_30_P01019924 |  |
| A_55_P2051270 | Tctex1d1 |
| A_30_P01027278 |  |
| A_55_P2069550 |  |
| A_55_P2113310 |  |
| A_55_P2213348 | 5330421C15Rik |
| A_30_P01019195 |  |
| A_55_P2106358 |  |
| A_30_P01026021 |  |
| A_55_P2027392 | Gpr146 |
| A_55_P2100290 | Adra1b |
| A_55_P1976200 | Lmtk3 |
| A_51_P111612 | Arrdc4 |
| A_30_P01029744 |  |
| A_30_P01031674 |  |
| A_30_P01024836 |  |
| A_55_P2132646 |  |
| A_51_P423976 | Crem |
| A_65_P01319 | Pde4b |
| A_55_P2001262 |  |
| A_51_P484998 | Hgf |
| A_55_P2178653 |  |
| A_55_P2416087 | C530005A16Rik |
| A_55_P2040600 | Exd1 |
| A_51_P117581 | Cables1 |
| A_55_P2157033 | Bace2 |
| A_55_P2144095 |  |
| A_55_P2127303 | Spsb2 |
| A_55_P2022629 | Oxct2b |
| A_55_P2108248 | Art4 |
| A_30_P01032657 |  |
| A_55_P1954835 | Ramp3 |
| A_55_P1973427 | Tex14 |
| A_55_P2053838 | Tnfaip3 |
| A_51_P491350 | Col4a2 |
| A_51_P438990 | Olfr911-ps1 |
| A_55_P2015887 | Wwox |
| A_55_P2224431 | 6330412A17Rik |
| A_51_P200544 | Tnip3 |
| A_55_P2153391 | Palm2 |
| A_55_P2090374 | Vmn1r68 |
| A_66_P106388 | Ms4a4c |
| A_55_P1958038 | Klra16 |
| A_55_P2003813 | Scn3b |
| A_55_P2270412 | C230096K16Rik |
| A_30_P01033617 |  |
| A_52_P799815 | Tmem171 |
| A_55_P2195172 | D930021N14 |
| A_51_P297069 | Tmod1 |
| A_30_P01019014 |  |
| A_30_P01018802 |  |
| A_55_P2148809 | Zan |
| A_55_P2042615 |  |
| A_51_P505868 | Lhfp |
| A_55_P2013184 | Atp2b3 |
| A_51_P186798 |  |
| A_55_P1969962 | Catsper3 |
| A_30_P01020171 |  |
| A_66_P109183 | Apold1 |
| A_55_P1996911 | Rasa3 |
| A_55_P1964896 | Gm3014 |
| A_55_P2256586 | Gm11783 |
| A_55_P2259896 | A130009E19Rik |
| A_52_P541833 | Vps37b |
| A_30_P01018712 |  |
| A_51_P121915 | BC089597 |
| A_30_P01028426 |  |
| A_55_P2126259 | Slc35e2 |
| A_55_P2174323 | Pgpep1l |
| A_30_P01018806 |  |
| A_55_P1955183 | Crxos |
| A_30_P01017739 |  |
| A_55_P1963046 | Prl3c1 |
| A_55_P2020361 | Lzts3 |
| A_51_P422685 | Zmat4 |
| A_66_P139805 | Crispld1 |
| A_55_P2181191 | Btg1 |
| A_55_P2168223 | Aicda |
| A_55_P2381821 | 6430706H07Rik |
| A_30_P01023753 |  |
| A_55_P2120064 | Gm2984 |
| A_30_P01028595 |  |
| A_55_P2269254 | 5730419F03Rik |
| A_30_P01027938 |  |
| A_55_P2128734 | Pbxip1 |
| A_51_P106249 | Cmtm2b |
| A_55_P2143516 |  |
| A_55_P1978416 | Il12rb2 |
| A_55_P1968763 | Shc4 |
| A_55_P2113673 | Eml1 |
| A_51_P112223 | Gsta4 |
| A_55_P2020035 |  |
| A_55_P2016064 |  |
| A_55_P2419021 | 9330188P03Rik |
| A_55_P2321453 | D11Ertd726e |
| A_52_P1093529 | Pik3r5 |
| A_55_P1981829 | Rhox8 |
| A_51_P170959 | Proz |
| A_30_P01031286 |  |
| A_51_P123676 | Synpo |
| A_30_P01032395 |  |
| A_30_P01020338 |  |
| A_52_P577388 | Epdr1 |
| A_65_P10913 | Tgfb2 |
| A_30_P01031663 |  |
| A_55_P2069802 | D5Ertd577e |
| A_52_P57317 | Fam19a3 |
| A_55_P1977628 | Pappa |
| A_55_P2146254 | Ifitm1 |
| A_30_P01026001 |  |
| A_52_P460957 | Crem |
| A_55_P2234084 |  |
| A_30_P01030451 |  |
| A_55_P2017826 | Myb |
| A_30_P01030387 |  |
| A_55_P1962284 | Klhl24 |
| A_30_P01023132 |  |
| A_30_P01020686 |  |
| A_51_P234253 | Sdcbp2 |
| A_51_P229664 | Cd27 |
| A_55_P1966568 |  |
| A_30_P01020841 |  |
| A_52_P22763 | Map2 |
| A_30_P01022829 |  |
| A_30_P01025657 |  |
| A_55_P2131168 | Sv2c |
| A_66_P117730 | Hapln1 |
| A_55_P2156638 | Gpr114 |
| A_51_P264825 | Lag3 |
| A_30_P01032069 |  |
| A_55_P2162543 |  |
| A_30_P01017626 |  |
| A_52_P190973 | Vcl |
| A_52_P491872 | D830046C22Rik |
| A_51_P253883 | Fam49a |
| A_51_P480190 | Spaca1 |
| A_52_P665675 | Abca1 |
| A_51_P206405 | Ptprz1 |
| A_30_P01023097 |  |
| A_30_P01022080 |  |
| A_55_P2087963 |  |
| A_55_P1976471 |  |
| A_51_P405397 | Ecm1 |
| A_55_P1987725 | Gria4 |
| A_51_P394847 | Gm11346 |
| A_30_P01022578 |  |
| A_30_P01018048 |  |
| A_55_P2133632 | Sipa1l2 |
| A_52_P163924 | Trpd52l3 |
| A_30_P01022282 |  |
| A_55_P2058270 | LOC101056056 |
| A_55_P2149931 | Arap2 |
| A_55_P1978696 | Abcb4 |
| A_55_P1991381 | Adam34 |
| A_66_P124179 | Atp6v0d2 |
| A_55_P2134877 | Eml1 |
| A_55_P2257076 |  |
| A_55_P2069485 | Ptpn13 |
| A_66_P105801 | Igf1r |
| A_55_P1975690 | Bicc1 |
| A_55_P2148935 |  |
| A_55_P2007646 | Cryaa |
| A_51_P340170 | Il20ra |
| A_55_P2134246 | Serpinb9 |
| A_30_P01024596 |  |
| A_55_P2090330 | Kcnmb4 |
| A_30_P01019779 |  |
| A_55_P2004551 | Klra1 |
| A_30_P01018759 |  |
| A_55_P2096127 |  |
| A_55_P2370250 | Syn3 |
| A_30_P01031842 |  |
| A_55_P2118866 | Cmah |
| A_55_P1962004 |  |
| A_55_P2074144 | Tmprss11e |
| A_30_P01019251 |  |
| A_55_P1989772 | Sqrdl |
| A_55_P1986596 | Cacna1h |
| A_66_P101261 | Gm3367 |
| A_55_P2143219 | Rasgrp2 |
| A_55_P1968024 |  |
| A_55_P2021398 |  |
| A_55_P1975045 | Sgcg |
| A_30_P01031226 |  |
| A_30_P01027578 |  |
| A_55_P2087265 | Ifitm1 |
| A_55_P2030672 | Gm1587 |
| A_30_P01020408 |  |
| A_30_P01021308 |  |
| A_55_P2249379 | Gm16982 |
| A_55_P2115567 | Slc26a1 |
| A_30_P01030530 |  |
| A_30_P01029489 |  |
| A_51_P185292 | 4930581F22Rik |
| A_55_P2174847 | Olfr566 |
| A_52_P56682 | Sla2 |
| A_55_P2056533 | Ntrk3 |
| A_55_P2129407 | 4932431P20Rik |
| A_55_P1989765 |  |
| A_51_P470079 | Il1r2 |
| A_55_P2113703 | Spib |
| A_51_P352452 | Dbx2 |
| A_55_P1960846 | 4933408B17Rik |
| A_55_P2144526 | Fam65b |
| A_55_P2112693 | Sipa1l1 |
| A_55_P2110497 | Ddc |
| A_30_P01033126 |  |
| A_55_P2117164 | Tmem106b |
| A_55_P2157134 | Rmnd5a |
| A_55_P2367255 | 4833421G17Rik |
| A_55_P2132781 | Slc16a2 |
| A_52_P68702 | Frmd4b |
| A_30_P01022421 |  |
| A_30_P01032372 |  |
| A_51_P254646 | Jdp2 |
| A_55_P2035843 |  |
| A_55_P2165199 | Cxcr6 |
| A_55_P2172999 | Ptpn13 |
| A_55_P2105239 | Defb47 |
| A_51_P460279 | Fam154a |
| A_55_P1989813 | Gcm1 |
| A_51_P429335 | Prss16 |
| A_55_P1964594 |  |
| A_55_P1961608 | Ypel4 |
| A_55_P1985831 |  |
| A_30_P01029085 |  |
| A_55_P2029902 | Gab3 |
| A_55_P2129469 |  |
| A_51_P414126 | Rab19 |
| A_55_P2185890 | Cfh |
| A_55_P2011390 | Tead1 |
| A_55_P2062058 | Dbndd2 |
| A_30_P01028164 |  |
| A_66_P109986 | Cd33 |
| A_30_P01029324 |  |
| A_55_P2150476 |  |
| A_55_P2077522 |  |
| A_51_P483280 | Prnp |
| A_55_P2277620 | Gm16523 |
| A_30_P01022763 |  |
| A_55_P2268790 | 4930445G23Rik |
| A_55_P2258567 | D930043N17Rik |
| A_55_P2019949 |  |
| A_52_P585124 | Cxcr4 |
| A_55_P1968789 | Rasa3 |
| A_55_P2030030 | Adssl1 |
| A_55_P2013128 |  |
| A_55_P2070494 | Vmn2r60 |
| A_55_P2038347 | Acot3 |
| A_66_P111534 | 5430431A17Rik |
| A_55_P2137049 | AA467197 |
| A_30_P01022950 |  |
| A_30_P01027369 |  |
| A_55_P2058601 | LOC102636451 |
| A_55_P2152771 | Lhfpl2 |
| A_55_P2027979 | Impg2 |
| A_55_P2156126 | Sema6c |
| A_30_P01027097 |  |
| A_55_P2066553 | Oacyl |
| A_52_P236398 |  |
| A_52_P203560 | Fzd10 |
| A_55_P2028837 | Tspan2 |
| A_51_P451458 | Mamdc2 |
| A_55_P2058671 | Zfyve28 |
| A_30_P01025472 |  |
| A_55_P2055189 | Kcng1 |
| A_55_P1991874 | Dcpp3 |
| A_55_P2361932 | Far2os2 |
| A_55_P2107247 | Tssk5 |
| A_30_P01031426 |  |
| A_55_P1962699 |  |
| A_55_P2050747 | 4930557B15Rik |
| A_51_P242930 | Lat2 |
| A_51_P463187 | Tbc1d2b |
| A_30_P01021805 |  |
| A_55_P2058195 | Fsip2 |
| A_55_P2299275 | Olfr22-ps1 |
| A_52_P108850 | St8sia1 |
| A_30_P01032338 |  |
| A_52_P173442 | Wscd2 |
| A_55_P2438722 | Ifnar1 |
| A_30_P01026506 |  |
| A_30_P01021489 |  |
| A_51_P262721 | 0610009L18Rik |
| A_55_P2068515 | Gm5083 |
| A_55_P2064351 | Vipr1 |
| A_30_P01028109 |  |
| A_55_P2147487 | Cyth3 |
| A_51_P103541 | Cacna1s |
| A_30_P01033260 |  |
| A_55_P1971009 | Gzme |
| A_30_P01024586 |  |
| A_55_P2016114 | Fasl |
| A_65_P18181 | Runx2 |
| A_52_P14526 | Zyg11b |
| A_55_P2167930 | Dmd |
| A_55_P2121595 | Scml4 |
| A_55_P2410325 | 2610028D06Rik |
| A_55_P2059657 |  |
| A_30_P01023293 |  |
| A_55_P2199118 | Bend4 |
| A_30_P01032207 |  |
| A_30_P01031205 |  |
| A_55_P1967002 |  |
| A_30_P01032896 |  |
| A_55_P2133165 | Wwc1 |
| A_55_P2118609 | St6galnac1 |
| A_52_P663526 | Nmrk1 |
| A_55_P1981719 | Rreb1 |
| A_30_P01033663 |  |
| A_52_P585907 | Pla2g4f |
| A_55_P2180854 | Mrgprg |
| A_30_P01027293 |  |
| A_30_P01029470 |  |
| A_55_P2107383 | Vmn2r96 |
| A_55_P2401971 | AU015680 |
| A_55_P2068248 |  |
| A_55_P2057577 | Ugt1a6a |
| A_55_P2098175 | Olfr701 |
| A_52_P489778 | Ablim1 |
| A_55_P2278531 | C920008N22Rik |
| A_55_P2146683 | Gm4657 |
| A_55_P2014388 | Olfr54 |
| A_55_P1960479 |  |
| A_55_P1968028 | Tdgf1 |
| A_30_P01019129 |  |
| A_51_P155323 | Hc |
| A_55_P2192397 | A330023F24Rik |
| A_55_P1974178 | Pyhin1 |
| A_55_P2091359 | Padi2 |
| A_51_P461067 |  |
| A_55_P2062250 | Gm5524 |
| A_30_P01022466 |  |
| A_30_P01018533 |  |
| A_52_P156452 | Cmah |
| A_30_P01029190 |  |
| A_51_P363749 | Irf6 |
| A_51_P368823 | Grb7 |
| A_55_P1957413 | Lsp1 |
| A_52_P574668 | Nt5e |
| A_55_P1989514 | 2010016I18Rik |
| A_30_P01027566 |  |
| A_30_P01024360 |  |
| A_30_P01029607 |  |
| A_55_P2183208 | Prl2c1 |
| A_52_P212686 | Lrrk1 |
| A_52_P162500 | Dph3 |
| A_30_P01027605 |  |
| A_51_P382152 | Procr |
| A_65_P12530 | Pde4d |
| A_55_P1981200 | Grm1 |
| A_55_P1988975 | Ms4a4b |
| A_55_P2184945 | Mageb16 |
| A_30_P01031942 |  |
| A_66_P108059 | Ttc39c |
| A_51_P484111 | Matn2 |
| A_55_P2165249 | Papln |
| A_55_P2228953 | 4633402D09Rik |
| A_30_P01018318 |  |
| A_55_P2130660 | Slitrk4 |
| A_52_P532227 | S1pr1 |
| A_30_P01030847 |  |
| A_30_P01028877 |  |
| A_30_P01018107 |  |
| A_52_P140005 | Nipal1 |
| A_52_P622850 | Hes5 |
| A_51_P176972 | Amigo2 |
| A_30_P01029731 |  |
| A_55_P2040497 | Gm11517 |
| A_55_P2123566 | Sipa1l2 |
| A_55_P2161045 |  |
| A_52_P390127 | Klrc1 |
| A_52_P118706 | 9630013D21Rik |
| A_55_P1961761 | Dcc |
| A_51_P416858 | Myl1 |
| A_66_P114641 | Cdh18 |
| A_30_P01030359 |  |
| A_51_P215475 | Ptprb |
| A_51_P246773 | Sesn3 |
| A_55_P2106763 | Pxdc1 |
| A_30_P01019330 |  |
| A_51_P493987 | Moxd1 |
| A_30_P01023895 |  |
| A_30_P01019088 |  |
| A_30_P01033069 |  |
| A_55_P1994289 | Gm10791 |
| A_52_P553890 | Itgb3 |
| A_30_P01028896 |  |
| A_55_P2054409 | Pira2 |
| A_30_P01032087 |  |
| A_55_P2353079 | LOC102631735 |
| A_55_P2008093 | Vmn2r91 |
| A_55_P2023161 | Gm13084 |
| A_30_P01029619 |  |
| A_30_P01023506 |  |
| A_66_P111562 | Ccnd1 |
| A_30_P01017761 |  |
| A_30_P01031709 |  |
| A_52_P23225 | Gpc3 |
| A_30_P01027076 |  |
| A_30_P01023050 |  |
| A_55_P2117465 | Gm6559 |
| A_55_P2243828 | LOC552901 |
| A_30_P01021882 |  |
| A_52_P602669 | Serpinb6d |
| A_55_P2106583 |  |
| A_55_P1974932 | Olfr668 |
| A_55_P1954331 | Murc |
| A_30_P01019065 |  |
| A_55_P2113384 |  |
| A_30_P01019463 |  |
| A_55_P2135986 | Ms4a4c |
| A_55_P2103948 |  |
| A_55_P2186605 | Cacnb2 |
| A_66_P126293 | Itgb3 |
| A_55_P2105416 | Gm10319 |
| A_51_P466731 | Olfr959 |
| A_51_P250058 | Epas1 |
| A_55_P2047356 | Gm4868 |
| A_55_P2145224 |  |
| A_55_P2272748 |  |
| A_30_P01020519 |  |
| A_55_P1973833 | Astl |
| A_52_P836852 | Txk |
| A_52_P71686 | Atp6v0d2 |
| A_55_P2069052 | Sacs |
| A_30_P01025429 |  |
| A_30_P01032232 |  |
| A_55_P2283116 | 4930478P22Rik |
| A_30_P01022191 |  |
| A_55_P2099620 | Hmx2 |
| A_55_P2044922 | Gpr82 |
| A_30_P01030786 |  |
| A_51_P142896 | Cd59a |
| A_55_P2287260 | Snap23 |
| A_55_P2048224 |  |
| A_30_P01018395 |  |
| A_52_P58359 | Tlx1 |
| A_55_P2020338 | Scml4 |
| A_30_P01031489 |  |
| A_30_P01024761 |  |
| A_55_P2131143 | Dcaf17 |
| A_66_P116314 |  |
| A_55_P1997450 |  |
| A_51_P487487 | Speer4d |
| A_51_P149267 | LOC101056131 |
| A_55_P2108012 | Fam78b |
| A_30_P01023920 |  |
| A_55_P2086954 |  |
| A_30_P01017444 |  |
| A_30_P01020712 |  |
| A_55_P2112005 | Tff1 |
| A_55_P1972659 | Spaca1 |
| A_55_P2032009 | Cc2d2b |
| A_55_P2104835 | P2rx4 |
| A_55_P2330173 | 4930474G06Rik |
| A_30_P01022638 |  |
| A_55_P2248150 | 4930405A10Rik |
| A_30_P01030603 |  |
| A_51_P391445 | Ifngr1 |
| A_55_P2061273 | Tbx6 |
| A_55_P2026894 |  |
| A_55_P2155146 | Klrc3 |
| A_30_P01030998 |  |
| A_55_P2162935 | Ntn1 |
| A_30_P01032852 |  |
| A_55_P2017677 | Cap2 |
| A_30_P01018939 |  |
| A_30_P01028549 |  |
| A_55_P2020726 | Gm16505 |
| A_55_P2030903 | Rsph4a |
| A_55_P2076744 |  |
| A_30_P01018535 |  |
| A_55_P2022504 | 1700019B21Rik |
| A_30_P01027098 |  |
| A_55_P2037962 | Trpm6 |
| A_55_P1983508 | Nr4a2 |
| A_55_P2094060 | Gzma |
| A_55_P2032489 |  |
| A_30_P01025834 |  |
| A_52_P614207 |  |
| A_30_P01027590 |  |
| A_30_P01017808 |  |
| A_30_P01030286 |  |
| A_55_P2082319 | BC094916 |
| A_55_P2391674 | 4931407E12Rik |
| A_52_P172014 | Ramp1 |
| A_30_P01018833 |  |
| A_51_P471088 | Vmn1r24 |
| A_55_P2092492 | Il18r1 |
| A_55_P2129771 | Prrt1 |
| A_30_P01031851 |  |
| A_55_P2112772 | Gabra3 |
| A_55_P2150377 |  |
| A_30_P01019182 |  |
| A_30_P01021265 |  |
| A_55_P2101340 | Ramp3 |
| A_51_P312121 | Xdh |
| A_55_P2024046 | Slc16a5 |
| A_55_P1990398 | Vmn2r48 |
| A_52_P500274 | Ntrk3 |
| A_30_P01024455 |  |
| A_55_P2054261 | C2cd4b |
| A_30_P01020856 |  |
| A_55_P2056403 | Speer4f |
| A_52_P483336 | Ms4a1 |
| A_55_P2257765 | Gm7111 |
| A_30_P01022071 |  |
| A_30_P01032178 |  |
| A_30_P01024457 |  |
| A_55_P2031403 | Smim24 |
| A_30_P01019202 |  |
| A_55_P2045928 | Btnl10 |
| A_30_P01025230 |  |
| A_30_P01022726 |  |
| A_55_P2014516 |  |
| A_55_P2164253 | 2010015L04Rik |
| A_30_P01029367 |  |
| A_51_P381618 | Pla1a |
| A_30_P01024417 |  |
| A_30_P01022468 |  |
| A_55_P2149288 | Cmya5 |
| A_30_P01029943 |  |
| A_30_P01020320 |  |
| A_30_P01026503 |  |
| A_55_P1961567 | Ypel3 |
| A_30_P01019556 |  |
| A_30_P01029037 |  |
| A_55_P2162815 | Fbxo31 |
| A_55_P2035509 | Pyhin1 |
| A_30_P01028513 |  |
| A_52_P514407 | Klra15 |
| A_30_P01030158 |  |
| A_30_P01030095 |  |
| A_55_P2064741 | Nmb |
| A_30_P01018793 |  |
| A_55_P2105436 | Foxp1 |
| A_51_P419246 | 5830416P10Rik |
| A_55_P1988368 | Upp1 |
| A_55_P2017169 |  |
| A_30_P01022733 |  |
| A_55_P1979950 |  |
| A_52_P161495 | Bcl6 |
| A_52_P570820 | Slc22a22 |
| A_30_P01024947 |  |
| A_51_P298066 | Clnk |
| A_55_P2237440 | Gm13999 |
| A_30_P01028422 |  |
| A_30_P01021760 |  |
| A_30_P01023427 |  |
| A_55_P2103060 |  |
| A_55_P2121484 | Smo |
| A_55_P2089342 | Gm13547 |
| A_52_P37091 | Magi1 |
| A_30_P01031833 |  |
| A_55_P1981830 |  |
| A_52_P648715 | Triml1 |
| A_55_P2035986 | E330020D12Rik |
| A_55_P2081590 | Olfr1320 |
| A_55_P2108933 | Dao |
| A_55_P1959525 | Wbscr25 |
| A_30_P01020636 |  |
| A_55_P2184434 | Eomes |
| A_30_P01024775 |  |
| A_30_P01024742 |  |
| A_55_P2070296 | D330025C20Rik |
| A_55_P2008016 | Armc3 |
| A_55_P2149921 |  |
| A_30_P01028539 |  |
| A_55_P2208682 | B230334C09Rik |
| A_30_P01032937 |  |
| A_55_P1973501 | Ceacam16 |
| A_52_P197402 | Tbc1d30 |
| A_51_P247168 | Wdr96 |
| A_55_P2160416 | Acoxl |
| A_51_P202801 | Abcb9 |
| A_52_P554650 | Gzmd |
| A_55_P2011445 | Capn13 |
| A_55_P1962209 | Cxcr6 |
| A_66_P135800 |  |
| A_51_P110341 | Scgb3a1 |
| A_52_P500979 | Pcsk1 |
| A_55_P1967591 |  |
| A_55_P2023290 | 1110032A03Rik |
| A_30_P01028087 |  |
| A_55_P2058127 | Pde4dip |
| A_55_P1984401 | Shank2 |
| A_30_P01032347 |  |
| A_51_P456870 | Foxj1 |
| A_55_P1998892 | Smox |
| A_51_P463765 | Timp3 |
| A_55_P2197638 | 1110046J04Rik |
| A_66_P140688 |  |
| A_66_P103231 |  |
| A_30_P01023911 |  |
| A_55_P2010396 | Pde4d |
| A_30_P01023370 |  |
| A_51_P317640 | Tgfb2 |
| A_55_P2133943 |  |
| A_55_P2227154 | A530041M06Rik |
| A_30_P01031611 |  |
| A_51_P419637 | Dclk3 |
| A_52_P370935 | Glcci1 |
| A_55_P2063237 | Dusp5 |
| A_30_P01026901 |  |
| A_30_P01028225 |  |
| A_55_P2206269 | 9130403I23Rik |
| A_55_P1957867 | Gm3161 |
| A_30_P01018083 |  |
| A_52_P355084 | Metrnl |
| A_51_P136294 | Ms4a4b |
| A_55_P2035029 | Mup-ps12 |
| A_51_P285047 | Cd160 |
| A_55_P1984322 | BC042761 |
| A_66_P118299 | Gm6846 |
| A_55_P1962084 |  |
| A_55_P2084308 | Nid1 |
| A_55_P2060269 | 1700091H14Rik |
| A_55_P2004652 | Klrc1 |
| A_55_P2093614 | Dennd4a |
| A_51_P438967 | Gpnmb |
| A_55_P1954998 | Phf1 |
| A_55_P2122020 | Klf4 |
| A_30_P01030639 |  |
| A_51_P483013 | Arel1 |
| A_30_P01031710 |  |
| A_55_P2000439 | Ptprz1 |
| A_55_P2078123 | Rora |
| A_55_P2066299 | Gpr137b |
| A_66_P119376 | Kctd12 |
| A_30_P01023079 |  |
| A_66_P139530 | Gm5107 |
| A_51_P212420 | Lama4 |
| A_30_P01018590 |  |
| A_66_P136844 | Dppa2 |
| A_30_P01033579 |  |
| A_55_P2041457 |  |
| A_55_P2024150 | Slco6b1 |
| A_55_P2417434 | E130215H24Rik |
| A_30_P01022989 |  |
| A_55_P2054362 | Cx3cr1 |
| A_55_P2290914 | Trav3n-3 |
| A_55_P1957209 | 4932443I19Rik |
| A_55_P2361652 | C230085N15Rik |
| A_51_P372743 | Frmpd3 |
| A_55_P2141754 | Tcl1b2 |
| A_55_P2060991 | BC005764 |
| A_55_P2007339 | Gm16489 |
| A_55_P2288670 | E130118H10Rik |
| A_55_P2033105 | Cdcp1 |
| A_30_P01032241 |  |
| A_55_P2402258 | 4930432J09Rik |
| A_55_P2081488 | Pglyrp1 |
| A_55_P2332731 |  |
| A_55_P2064928 | Nebl |
| A_51_P497317 | Tcp11l2 |
| A_30_P01025765 |  |
| A_55_P2202524 | 9330162012Rik |
| A_66_P122719 |  |
| A_55_P2079269 |  |
| A_52_P566396 | Rnf122 |
| A_51_P120830 | Mmp10 |
| A_55_P2144556 | Flrt3 |
| A_30_P01022541 |  |
| A_30_P01017796 |  |
| A_30_P01020946 |  |
| A_55_P1961429 | Vmn1r131 |
| A_66_P117484 | Gm14317 |
| A_51_P142153 | Filip1l |
| A_51_P281778 | Igsf23 |
| A_51_P505617 | Il18r1 |
| A_55_P1966432 | Gstm1 |
| A_55_P2076196 | Mup17 |
| A_30_P01018690 |  |
| A_30_P01029325 |  |
| A_55_P2006852 | Dgkb |
| A_51_P420128 | Fam219aos |
| A_52_P456134 | Dgat1 |
| A_52_P660477 | Spo11 |
| A_55_P2036086 |  |
| A_51_P132170 | Ccdc141 |
| A_51_P448127 | Khdc3 |
| A_30_P01025555 |  |
| A_55_P2175925 |  |
| A_55_P2026779 | 1700091H14Rik |
| A_51_P295967 | Proc |
| A_30_P01019196 |  |
| A_30_P01022138 |  |
| A_55_P2223851 |  |
| A_51_P469688 | Syt17 |
| A_30_P01024849 |  |
| A_30_P01023056 |  |
| A_30_P01019238 |  |
| A_51_P259975 | Aspa |
| A_55_P1987291 | 4833424O15Rik |
| A_30_P01018325 |  |
| A_55_P2138739 |  |
| A_30_P01018311 |  |
| A_55_P2161219 | Thsd7a |
| A_55_P2022519 | Tmem108 |
| A_30_P01024167 |  |
| A_51_P196695 | Il7r |
| A_55_P2084739 | Gcnt1 |
| A_30_P01029056 |  |
| A_55_P1971010 | Gzme |
| A_52_P423247 | Pde4b |
| A_30_P01032466 |  |
| A_55_P1960999 | Pigr |
| A_30_P01027371 |  |
| A_55_P2057528 | Arl4d |
| A_52_P243391 | Sema4f |
| A_55_P2212006 | 2410087M07Rik |
| A_51_P290921 | Sytl2 |
| A_51_P499838 | Bst1 |
| A_55_P2062449 | Pde6a |
| A_55_P2058297 | Sgip1 |
| A_55_P2156140 | Tcerg1l |
| A_55_P2307496 |  |
| A_51_P448147 | Gimap7 |
| A_55_P2214487 | E330013P08Rik |
| A_51_P205385 | Uox |
| A_51_P149714 | Ms4a6d |
| A_55_P2279498 | 4933438K21Rik |
| A_55_P2070331 | 5830416P10Rik |
| A_51_P490795 | Mxd1 |
| A_52_P244702 | Tcf7 |
| A_52_P117408 | Tg |
| A_55_P2113498 | Klrd1 |
| A_55_P2419299 | 1110006E14Rik |
| A_55_P2080476 |  |
| A_51_P330452 | Olfr1414 |
| A_52_P42194 | Svil |
| A_51_P493117 | Slc16a9 |
| A_30_P01027848 |  |
| A_52_P605517 | Phactr1 |
| A_55_P2007831 | Ccdc172 |
| A_55_P2272979 | Tdrd5 |
| A_55_P2138291 | Arl14epl |
| A_55_P1970915 | Kdm4d |
| A_51_P290556 | Nyap2 |
| A_55_P2361437 | Pitpnc1 |
| A_55_P2067362 | Dpep2 |
| A_55_P2083297 | E230025N22Rik |
| A_30_P01032949 |  |
| A_55_P2003266 | Fam107a |
| A_55_P1965015 | 6530402F18Rik |
| A_52_P629895 | Adh1 |
| A_55_P2068947 | Vmn1r203 |
| A_55_P1953630 | E330016L19Rik |
| A_55_P2200319 | A630014C17Rik |
| A_30_P01028171 |  |
| A_55_P2063126 | Inadl |
| A_55_P2221647 | AI605517 |
| A_55_P2167788 | Gm6093 |
| A_52_P279425 | Cd96 |
| A_55_P2004906 | 4930407I19Rik |
| A_55_P2079552 | 4930524N10Rik |
| A_55_P2237360 | 1700008O03Rik |
| A_30_P01031384 |  |
| A_55_P1968433 | Agpat9 |
| A_55_P2006265 | Olfr1446 |
| A_30_P01023045 |  |
| A_55_P2075469 | Baalc |
| A_55_P2156806 | Pom121l2 |
| A_55_P2180744 | Clstn3 |
| A_30_P01031856 |  |
| A_55_P2004536 | Klra4 |
| A_30_P01028043 |  |
| A_52_P412506 | Mup5 |
| A_55_P2012171 | Spata6 |
| A_55_P2019699 | Samhd1 |
| A_30_P01032986 |  |
| A_51_P156158 | Phlpp1 |
| A_55_P2119377 | Klri2 |
| A_55_P2154107 | Gcm1 |
| A_55_P2004442 | Nr2e1 |
| A_30_P01028500 |  |
| A_55_P2228297 |  |
| A_66_P125770 | Gm3942 |
| A_55_P2129261 | Arhgap36 |
| A_51_P457196 | Sfrp4 |
| A_52_P649210 |  |
| A_55_P2010152 | Sell |
| A_55_P2206461 | A930006K02Rik |
| A_55_P1960197 | P2ry14 |
| A_55_P2057777 | Fgfr1 |
| A_30_P01023824 |  |
| A_55_P2169923 | Cacna1s |
| A_55_P2062802 |  |
| A_55_P2223282 | B130019D13Rik |
| A_30_P01021320 |  |
| A_30_P01027981 |  |
| A_52_P517984 | Usp50 |
| A_66_P128761 | Pydc3 |
| A_30_P01025525 |  |
| A_55_P2102838 |  |
| A_55_P2119548 | Gm13298 |
| A_55_P2136752 | Ermn |
| A_55_P2008926 | Slc17a3 |
| A_51_P185763 | Slc46a2 |
| A_55_P2170836 | Gm14149 |
| A_55_P2046149 | Kremen1 |
| A_55_P1957871 |  |
| A_55_P2013356 | Renbp |
| A_55_P2058028 |  |
| A_55_P2051622 | Gm8479 |
| A_55_P1971938 | Atp2b2 |
| A_66_P105596 |  |
| A_51_P473498 | Gpr171 |
| A_55_P2011491 |  |
| A_30_P01023578 |  |
| A_30_P01032461 |  |
| A_55_P2060376 | Gzmg |
| A_51_P464900 | Gabbr1 |
| A_30_P01021842 |  |
| A_51_P446131 | Gipc2 |
| A_55_P2072000 | Pcmtd1 |
| A_55_P2109877 | Gm4718 |
| A_55_P2174743 | Akap7 |
| A_51_P357914 | Pdc |
| A_55_P2022773 | Glcci1 |
| A_55_P2056674 | Lrrk1 |
| A_55_P2283551 | 2810455B08Rik |
| A_55_P2025483 | Rfesd |
| A_55_P2246344 | 5830407E08Rik |
| A_55_P1977855 | Sall3 |
| A_55_P2100197 |  |
| A_30_P01021190 |  |
| A_55_P2077501 |  |
| A_30_P01032661 |  |
| A_55_P2019838 | LOC102635555 |
| A_55_P2025937 |  |
| A_30_P01020571 |  |
| A_52_P88722 | Senp7 |
| A_55_P1964138 | Pign |
| A_55_P2368680 | Gm10741 |
| A_51_P303180 | Fam114a1 |
| A_55_P2130627 | Smim24 |
| A_51_P187602 | Serpinb5 |
| A_52_P517098 | Il18rap |
| A_55_P2106280 | Gm10684 |
| A_51_P456465 | Cldn10 |
| A_52_P72965 | Unc80 |
| A_65_P14951 | Cblb |
| A_55_P2077515 |  |
| A_52_P730743 |  |
| A_55_P1989738 | 4930426L09Rik |
| A_52_P478444 |  |
| A_55_P2074656 | Padi2 |
| A_55_P2425761 | C530043K16Rik |
| A_55_P2181963 | Gm8369 |
| A_51_P276479 | 4930486L24Rik |
| A_55_P1978316 | Adamts14 |
| A_52_P232813 | Cxcl3 |
| A_30_P01029179 |  |
| A_55_P1988658 | 4930558N11Rik |
| A_30_P01030381 |  |
| A_30_P01031149 |  |
| A_51_P413740 | Ftcd |
| A_55_P2269289 | Dgkg |
| A_30_P01032026 |  |
| A_55_P2378486 | Kcnma1 |
| A_30_P01027555 |  |
| A_55_P2050988 |  |
| A_51_P326229 | Ddx25 |
| A_55_P2393734 | 4933421H12Rik |
| A_55_P2008835 |  |
| A_55_P2111380 | Ctnnd2 |
| A_30_P01021778 |  |
| A_30_P01026400 |  |
| A_55_P2048448 | Klra23 |
| A_30_P01024983 |  |
| A_55_P2005470 | Mfap4 |
| A_30_P01031285 |  |
| A_30_P01024637 |  |
| A_30_P01030229 |  |
| A_55_P2062573 | C1qtnf3 |
| A_55_P2013043 | Serpinb6b |
| A_55_P2009449 | Pnma2 |
| A_55_P2132651 | Wisp1 |
| A_55_P2021585 | Tff1 |
| A_30_P01032380 |  |
| A_30_P01023510 |  |
| A_30_P01031079 |  |
| A_30_P01020553 |  |
| A_55_P1969698 | 5430402E10Rik |
| A_55_P2335768 | 4831407H17Rik |
| A_30_P01024729 |  |
| A_51_P422540 | Paqr8 |
| A_51_P440460 | Hip1r |
| A_30_P01017646 |  |
| A_30_P01022515 |  |
| A_30_P01021571 |  |
| A_30_P01022305 |  |
| A_55_P2109633 | Tcf7 |
| A_55_P2083629 | Tle2 |
| A_30_P01032048 |  |
| A_30_P01029579 |  |
| A_55_P2216822 | 4930551O13Rik |
| A_55_P2386256 | D130062J10Rik |
| A_55_P2454784 | Ifnar1 |
| A_51_P144264 | Klf2 |
| A_55_P2135526 | Gzmc |
| A_55_P2315921 | 5330431K02Rik |
| A_55_P2021981 | Ctsw |
| A_30_P01018708 |  |
| A_52_P123655 | A630023P12Rik |
| A_55_P2364516 | AA060545 |
| A_30_P01029394 |  |
| A_55_P2178800 | Ugt1a10 |
| A_30_P01027083 |  |
| A_55_P1982747 | Slc8a3 |
| A_55_P2088375 | Tnnt3 |
| A_55_P1954555 | LOC102637894 |
| A_52_P64687 | Camk2n1 |
| A_55_P2036007 | Rai2 |
| A_51_P163953 | Nsg2 |
| A_30_P01023919 |  |
| A_30_P01018847 |  |
| A_30_P01019879 |  |
| A_30_P01032204 |  |
| A_30_P01018751 |  |
| A_52_P667477 | Fyco1 |
| A_55_P2397400 |  |
| A_55_P2373852 | 2310058N22Rik |
| A_52_P363216 | Gcnt2 |
| A_55_P2027077 | Shc2 |
| A_30_P01027939 |  |
| A_55_P2087205 | 9530077C14Rik |
| A_55_P2316682 | A830011I04 |
| A_55_P2281818 | LOC433347 |
| A_30_P01019369 |  |
| A_51_P116906 | Rapgef3 |
| A_51_P489138 | Sptb |
| A_30_P01023736 |  |
| A_52_P381430 | Tbc1d4 |
| A_55_P2374337 | A130071D04Rik |
| A_55_P2126269 | Nmb |
| A_52_P681310 | Plaur |
| A_51_P304397 | Cpm |
| A_55_P2079560 | Lilra6 |
| A_55_P2096310 |  |
| A_51_P420577 | Olfr983 |
| A_55_P1997126 | Ctse |
| A_55_P2000628 | Dusp7 |
| A_55_P2259456 | 4933425B07Rik |
| A_55_P2079579 | Pira7 |
| A_30_P01031257 |  |
| A_55_P2185068 | Gm3002 |
| A_30_P01031240 |  |
| A_51_P394394 | Tspan2 |
| A_55_P2255944 | 9130002K18Rik |
| A_55_P2040245 | Piezo2 |
| A_52_P612137 | Runx1t1 |
| A_55_P2025248 | Mxd1 |
| A_30_P01018930 |  |
| A_55_P2232057 | AU022793 |
| A_51_P220343 | Wisp1 |
| A_55_P1982227 |  |
| A_30_P01024650 |  |
| A_30_P01025307 |  |
| A_55_P2028847 |  |
| A_55_P2173039 |  |
| A_30_P01028959 |  |
| A_66_P102878 | Olfr726 |
| A_55_P1965564 | Gm15085 |
| A_55_P2156515 |  |
| A_55_P2161347 | Acmsd |
| A_55_P2039646 |  |
| A_30_P01023052 |  |
| A_55_P2132888 | Sdcbp |
| A_30_P01024653 |  |
| A_30_P01018360 |  |
| A_30_P01021347 |  |
| A_51_P183051 | Upb1 |
| A_55_P2150737 |  |
| A_55_P2107542 | Pde4b |
| A_51_P245989 | Ccr2 |
| A_55_P2193424 | 6720420G18Rik |
| A_55_P2134616 | Med12l |
| A_55_P2150697 | Cpne4 |
| A_55_P2373987 | A730009E18Rik |
| A_30_P01019473 |  |
| A_52_P494622 | Nr4a2 |
| A_55_P2320263 | Caln1 |
| A_55_P2145465 | D5Ertd577e |
| A_51_P158545 | Ankk1 |
| A_66_P104815 | Ecm1 |
| A_52_P476731 | Fam110c |
| A_30_P01031678 |  |
| A_51_P240693 | Tecpr1 |
| A_30_P01025404 |  |
| A_30_P01031292 |  |
| A_30_P01030435 |  |
| A_51_P104418 | Dusp10 |
| A_55_P2329660 | Catsperd |
| A_55_P2007964 | Cx3cr1 |
| A_55_P2006677 | Gm7969 |
| A_55_P2218334 | 9430011C21Rik |
| A_52_P621588 | Ifnlr1 |
| A_55_P2109382 | Adora2a |
| A_30_P01030963 |  |
