# Supplementary material for: T cell–intrinsic prostaglandin E2-EP2/EP4 signaling is critical in pathogenic TH17 cell–driven inflammation
Source: J Allergy Clin Immunol. 2019 Feb;143(2):631–43. doi: 10.1016/j.jaci.2018.05.036 (PMC6354914; doi:10.1016/j.jaci.2018.05.036)
Supplement: Table E4 [file mmc6.docx]

| ProbeName | GeneSymbol |
| --- | --- |
| A_51_P124254 | Col4a1 |
| A_30_P01028640 |  |
| A_55_P2179074 | Ciita |
| A_55_P2143025 | Sema3c |
| A_30_P01033650 |  |
| A_55_P1983523 | Cd300ld |
| A_30_P01031906 |  |
| A_30_P01029988 |  |
| ERCC-00138_246 |  |
| A_30_P01021841 |  |
| A_55_P2126072 |  |
| A_55_P2350553 | 4933405D12Rik |
| A_55_P2079064 | Ppnr |
| A_30_P01033285 |  |
| A_51_P474459 | Socs3 |
| A_30_P01023629 |  |
| A_30_P01026288 |  |
| A_30_P01020935 |  |
| A_30_P01019221 |  |
| A_30_P01029761 |  |
| A_55_P2172058 | Trim55 |
| A_30_P01030183 |  |
| A_30_P01025039 |  |
| A_51_P212782 | Il1b |
| A_30_P01027263 |  |
| A_30_P01025011 |  |
| A_30_P01023923 |  |
| A_30_P01020331 |  |
| A_30_P01023521 |  |
| A_30_P01023091 |  |
| A_30_P01021631 |  |
| A_55_P2171303 | Bin3 |
| A_30_P01029928 |  |
| A_30_P01017753 |  |
| A_30_P01027585 |  |
| A_52_P430304 | Fam186b |
| A_30_P01026777 |  |
| A_55_P2325568 | 1700060J05Rik |
| A_30_P01033068 |  |
| A_30_P01022182 |  |
| A_55_P2052913 |  |
| A_55_P1960411 | Lrrc23 |
| A_30_P01019696 |  |
| A_30_P01020060 |  |
| A_30_P01020888 |  |
| A_55_P2091330 | Olfr871 |
| A_55_P2272830 |  |
| A_55_P2376363 | 4930432F04Rik |
| A_55_P2346859 | C630007K24Rik |
| A_55_P2014034 | Gm7285 |
| A_52_P137765 | Lmna |
| A_55_P2164265 | Prl7c1 |
| A_30_P01030364 |  |
| A_30_P01027780 |  |
| A_51_P127738 | Scn2a1 |
| A_55_P2383523 | 1700123I01Rik |
| A_30_P01021373 |  |
| A_51_P140803 | Slco1b2 |
| A_51_P160544 | Efemp2 |
| A_55_P1970876 | Olfr389 |
| A_30_P01021518 |  |
| A_30_P01020765 |  |
| A_66_P114333 | Tlr12 |
| A_30_P01028455 |  |
| A_30_P01032259 |  |
| A_30_P01021422 |  |
| A_55_P2085727 | Stk38 |
| A_30_P01021468 |  |
| A_55_P2419483 | 4732460I02Rik |
| A_55_P1958039 | Klra16 |
| A_30_P01018127 |  |
| A_55_P2397504 | D130046C19Rik |
| A_55_P2198648 | 5830420C07Rik |
| A_55_P2018106 | Gm14085 |
| A_55_P1957866 | Gm3161 |
| A_55_P2199737 | Dlgap2 |
| A_52_P410685 | Krt7 |
| A_30_P01018893 |  |
| A_55_P2088178 | Cbln2 |
| A_30_P01026963 |  |
| A_55_P2026420 | Pou6f1 |
| A_55_P2066878 | Kcnj13 |
| A_55_P2128582 |  |
| A_55_P2092717 | Trim43b |
| A_30_P01023033 |  |
| A_55_P2056876 | Akap13 |
| A_51_P475342 | Chrnb1 |
| A_55_P2249556 | A630081D01Rik |
| A_30_P01025016 |  |
| A_30_P01031844 |  |
| A_55_P1997421 | Gm5416 |
| A_30_P01022699 |  |
| A_30_P01025043 |  |
| A_30_P01028130 |  |
| A_55_P1957277 | Obfc1 |
| A_30_P01020539 |  |
| A_55_P2403874 | D230014I24Rik |
| A_55_P1980868 | Gm6313 |
| A_30_P01023317 |  |
| A_30_P01022249 |  |
| A_30_P01026161 |  |
| A_55_P1998827 | Prim2 |
| A_55_P2019533 | Zscan4c |
| A_30_P01025818 |  |
| A_30_P01020406 |  |
| A_55_P2181029 |  |
| A_55_P2025038 | Cpe |
| A_30_P01031340 |  |
| A_30_P01022117 |  |
| A_55_P2162364 | Cacfd1 |
| A_55_P2046328 | Gm6225 |
| A_66_P110091 | Gm12371 |
| A_55_P2112982 | Tcl1b4 |
| A_30_P01028297 |  |
| A_55_P2043932 | Tmem8b |
| A_55_P2151638 | Klra15 |
| A_55_P2123123 | Zic5 |
| A_52_P401484 | Inha |
| A_52_P434055 | Birc3 |
| A_55_P1979575 | Shroom2 |
| A_55_P2200029 | Dleu2 |
| A_30_P01032387 |  |
| A_55_P2119633 | Gnal |
| A_66_P131931 | Aurkc |
| A_30_P01024656 |  |
| A_52_P497021 | Spred3 |
| A_55_P2165224 | 4930467J12Rik |
| A_30_P01026625 |  |
| A_55_P2171196 |  |
| A_30_P01027893 |  |
| A_51_P260265 | Hoxd4 |
| A_52_P616332 | Atp10d |
| A_55_P2143081 |  |
| A_30_P01027753 |  |
| A_66_P137660 | Fam166b |
| A_55_P2148418 | Vmn2r82 |
| A_52_P51078 | Ctsh |
| A_55_P1994290 | Gm10791 |
| A_55_P2061064 | Ggt5 |
| A_55_P2115260 |  |
| A_66_P136569 |  |
| A_30_P01028376 |  |
| A_52_P964651 | Fam65c |
| A_30_P01026211 |  |
| A_55_P2110171 |  |
| A_55_P2108086 |  |
| A_55_P2064386 | Gm4098 |
| A_30_P01026654 |  |
| A_30_P01030450 |  |
| A_30_P01027602 |  |
| A_55_P1999756 |  |
| A_55_P2248320 | C430003N24Rik |
| A_55_P1962289 |  |
| A_30_P01031102 |  |
| A_52_P464228 | 1700009N14Rik |
| A_66_P119841 | Ddx17 |
| A_55_P2329313 | C530014P21Rik |
| A_30_P01019408 |  |
| A_30_P01030630 |  |
| A_65_P03728 | St3gal1 |
| A_55_P2212498 | C030005K06Rik |
| A_55_P2162910 | Rtn1 |
| A_30_P01028299 |  |
| A_30_P01028569 |  |
| A_55_P1968895 | Prph |
| A_55_P2409336 | 4932429P19Rik |
| A_55_P2049262 | Gm16525 |
| A_30_P01028919 |  |
| A_30_P01032993 |  |
| A_55_P1955427 | Cux1 |
| A_55_P1958329 |  |
| A_30_P01018695 |  |
| A_30_P01018313 |  |
| A_55_P1988388 | Gm2347 |
| A_55_P1957245 |  |
| A_55_P2317341 |  |
| A_51_P477682 | Prss12 |
| A_55_P2090484 | Pde10a |
| A_55_P2250424 | 9530029O12Rik |
| A_30_P01028796 |  |
| A_30_P01030240 |  |
| A_55_P2212458 | LOC548102 |
| A_55_P2071716 | Klre1 |
| A_51_P141926 | Fxyd4 |
| A_55_P2234361 | Rnf150 |
| A_55_P2011700 | Gm3364 |
| A_55_P2086323 | BC051408 |
| A_55_P2052076 | Obox3 |
| A_51_P260740 | Pcdh7 |
| A_52_P146403 | Arhgef38 |
| A_55_P2190152 | 4921509J17Rik |
| A_51_P454873 | Npy |
| A_55_P2048912 | Themis2 |
| A_66_P134109 | Gm3331 |
| A_30_P01027969 |  |
| A_51_P195958 | Phlda1 |
| A_65_P10029 | Prdm2 |
| A_55_P2065140 | A930003A15Rik |
| A_52_P356093 | B3galt2 |
| A_55_P2022347 |  |
| A_51_P444447 | Cebpd |
| A_55_P2260052 | Gm17753 |
| A_55_P2050513 | Pgbd1 |
| A_55_P2007470 | Pdgfa |
| A_55_P2076064 | Dnah8 |
| A_55_P2008297 | Cd300a |
| A_51_P320357 | Grin2b |
| A_55_P2003199 | Setd1b |
| A_55_P1964672 | Krt28 |
| A_55_P2119927 |  |
| A_55_P2127977 |  |
| A_66_P137556 | Tle1 |
| A_55_P2293351 | Slc2a4rg-ps |
| A_30_P01017519 |  |
| A_30_P01023472 |  |
| A_30_P01033074 |  |
| A_55_P2070105 |  |
| A_30_P01019307 |  |
| A_30_P01029310 |  |
| A_30_P01022226 |  |
| A_30_P01029091 |  |
| A_66_P116860 | 5031434O11Rik |
| A_30_P01020677 |  |
| A_55_P1966155 | Wfdc6b |
| A_30_P01023532 |  |
| A_30_P01019630 |  |
| A_30_P01028922 |  |
| A_30_P01032113 |  |
| A_51_P497741 | Wdr95 |
| A_55_P1991500 | Obfc1 |
| A_55_P2346736 | A430105D02Rik |
| A_52_P594302 | Lrba |
| A_30_P01019783 |  |
| A_55_P2130695 | Armc4 |
| A_30_P01024134 |  |
| A_30_P01018888 |  |
| A_30_P01028942 |  |
| A_52_P458647 |  |
| A_55_P2025765 | Adam8 |
| A_52_P266132 | Fgl2 |
| A_30_P01020996 |  |
| A_55_P1977938 | Fcgbp |
| A_55_P2082519 | Olfr883 |
| A_30_P01032602 |  |
| A_55_P1958951 |  |
| A_30_P01021398 |  |
| A_30_P01024624 |  |
| A_55_P2075065 | Gm10471 |
| A_30_P01032240 |  |
| A_55_P2311208 | C130045F17Rik |
| A_30_P01018774 |  |
| A_52_P667287 | Cers6 |
| A_55_P2180481 | 1810020O05Rik |
| A_30_P01022128 |  |
| A_55_P2213968 | 4933416M07Rik |
| A_30_P01019634 |  |
| A_52_P508317 | Erlec1 |
| A_55_P1977473 | Dab2 |
| A_30_P01030677 |  |
| A_55_P2051094 | Rorc |
| A_55_P2112270 | Gm6556 |
| A_55_P2065506 |  |
| A_55_P2105403 | Nrxn3 |
| A_55_P2040873 | Gm867 |
| A_30_P01026907 |  |
| A_55_P2006625 |  |
| A_52_P468068 | Tchh |
| A_55_P1968103 | Pla2g2c |
| A_55_P2073905 |  |
| A_52_P655136 | Nlrc4 |
| A_66_P136801 | Peg13 |
| A_55_P2330560 | 1700120E14Rik |
| A_30_P01030273 |  |
| A_55_P2123045 | Olfr453 |
| A_55_P2066116 | Bcl3 |
| A_55_P2019362 | Deptor |
| A_30_P01022204 |  |
| A_30_P01022054 |  |
| A_52_P463235 | Ankrd33b |
| A_30_P01019135 |  |
| A_30_P01021718 |  |
| A_55_P2259889 | LOC102632493 |
| A_30_P01024278 |  |
| A_55_P2057941 | 1700049G17Rik |
| A_55_P2220342 | C230094B09Rik |
| A_55_P2307578 | E530011L22Rik |
| A_55_P1957459 | Lilrb4 |
| A_30_P01030411 |  |
| A_30_P01017989 |  |
| A_55_P2014427 | Il17re |
| A_55_P1974622 |  |
| A_55_P2077218 | Spef1 |
| A_30_P01028994 |  |
| A_30_P01030141 |  |
| A_30_P01025370 |  |
| A_55_P1964902 | Gm3014 |
| A_55_P2249849 | Sema6a |
| A_55_P1966470 |  |
| A_51_P184484 | Mmp13 |
| A_66_P127412 |  |
| A_55_P2126870 | Nsf |
| A_55_P2077884 | Kat6b |
| A_55_P2014987 | Gatsl2 |
| A_30_P01019902 |  |
| A_30_P01033039 |  |
| A_55_P2153496 | Ppp2r3d |
| A_55_P2005984 | Wfdc15b |
| A_30_P01031894 |  |
| A_55_P2218483 | A730093L10Rik |
| A_30_P01033525 |  |
| A_30_P01027071 |  |
| A_51_P150745 | Olfr1044 |
| A_30_P01032097 |  |
| A_55_P2004179 | Col2a1 |
| A_55_P2053236 |  |
| A_55_P2420983 | 6330575P09Rik |
| A_30_P01026306 |  |
| A_55_P2054410 |  |
| A_55_P2110615 | Slc37a2 |
| A_55_P1958160 | Sgce |
| A_55_P1952274 | C030016D13Rik |
| A_30_P01018042 |  |
| A_55_P1995195 | Fosl2 |
| A_55_P2158181 | Olfr39 |
| A_55_P1967158 | Gm5486 |
| A_55_P2006479 | 3300002I08Rik |
| A_30_P01029853 |  |
| A_30_P01025615 |  |
| A_55_P2023607 | Ikzf1 |
| A_51_P486188 | Pabpc2 |
| A_55_P2099466 |  |
| A_55_P2186634 | Pdik1l |
| A_52_P396312 | Cdh17 |
| A_55_P1979904 | Mup1 |
| A_55_P2020217 | Smok4a |
| A_30_P01032219 |  |
| A_55_P2093874 | Pbxip1 |
| A_55_P2185332 | Il17rc |
| A_55_P1999102 | Pi16 |
| A_30_P01025737 |  |
| A_30_P01019416 |  |
| A_30_P01022612 |  |
| A_52_P259508 | Prkcq |
| A_30_P01022038 |  |
| A_55_P2242089 | Cog5 |
| A_55_P2014352 |  |
| A_51_P305061 | 0610039K10Rik |
| A_55_P2041240 | Nav2 |
| A_55_P2040170 | Pmp22 |
| A_30_P01030554 |  |
| A_55_P2019620 | Vmn2r94 |
| A_30_P01022174 |  |
| A_55_P2408848 | Lgr4 |
| A_30_P01020577 |  |
| A_30_P01017726 |  |
| A_55_P2102464 |  |
| A_55_P1962781 |  |
| A_30_P01017817 |  |
| A_66_P114229 | Zic5 |
| A_30_P01024104 |  |
| A_30_P01021723 |  |
| A_30_P01027051 |  |
| A_55_P2082684 | Krt12 |
| A_55_P1966608 |  |
| A_55_P2266679 | Mettl21c |
| A_55_P2065429 |  |
| A_55_P2364315 | D030002E05Rik |
| A_30_P01021199 |  |
| A_30_P01030589 |  |
| A_30_P01019520 |  |
| A_51_P424641 | Sirt4 |
| A_30_P01032902 |  |
| A_30_P01024889 |  |
| A_55_P2137867 | Mpp3 |
| A_55_P2177712 | LOC102638515 |
| A_55_P2105180 | Bhmt |
| A_30_P01029327 |  |
| A_30_P01032254 |  |
| A_51_P284426 | Cstad |
| A_55_P2043782 | Trpm1 |
| A_30_P01024577 |  |
| A_55_P2325758 | A430054B03 |
| A_30_P01031901 |  |
| A_30_P01020426 |  |
| A_30_P01023251 |  |
| A_55_P2333126 | Mgea5 |
| A_55_P1977451 |  |
| A_55_P2065726 | Snx29 |
| A_30_P01021610 |  |
| A_55_P1966770 |  |
| A_55_P2125588 | Pdgfa |
| A_55_P2025775 |  |
| A_55_P2381921 | 4930406D14Rik |
| A_30_P01022472 |  |
| A_55_P2045158 | Mlxipl |
| A_30_P01032514 |  |
| A_55_P1959753 | Top3b |
| A_30_P01018341 |  |
| A_55_P2294109 | 1700047E10Rik |
| A_30_P01025798 |  |
| A_30_P01023172 |  |
| A_55_P1989812 |  |
| A_30_P01023118 |  |
| A_30_P01023385 |  |
| A_30_P01030643 |  |
| A_55_P2006008 | Serpinb1a |
| A_52_P398989 | Cytip |
| A_30_P01028292 |  |
| A_30_P01018167 |  |
| A_30_P01026636 |  |
| A_30_P01021342 |  |
| A_30_P01023902 |  |
| A_30_P01025624 |  |
| A_55_P2259500 | D130012P04Rik |
| A_30_P01031220 |  |
| A_55_P2246014 | C130051F05Rik |
| A_51_P121607 | 4930546C10Rik |
| A_30_P01026456 |  |
| A_30_P01018358 |  |
| A_55_P2086455 | Wscd2 |
| A_30_P01028737 |  |
| A_55_P2085015 | Vsx1 |
| A_51_P382849 | Emb |
| A_55_P2139464 |  |
| A_55_P1998781 | Zcchc16 |
| A_55_P1997544 |  |
| A_55_P2075569 | Smim23 |
| A_30_P01026305 |  |
| A_55_P2122633 | Airn |
| A_55_P1966721 | 9930105H17Rik |
| A_30_P01025549 |  |
| A_55_P2004016 | Crispld2 |
| A_55_P1958971 | Gm5538 |
| A_55_P2115062 | Cdc20b |
| A_30_P01032903 |  |
| A_52_P38964 | Sap25 |
| A_30_P01020315 |  |
| A_66_P125948 | Cass4 |
| A_55_P2302290 | E230012P03 |
| A_55_P2276224 | 9330175E14Rik |
| A_55_P2132014 | Tekt2 |
| A_30_P01019457 |  |
| A_55_P2098941 | 1700110I01Rik |
| A_30_P01026925 |  |
| A_30_P01023697 |  |
| A_51_P238722 | Cd93 |
| A_55_P2145626 | Krt82 |
| A_51_P234692 | Neat1 |
| A_30_P01018776 |  |
| A_55_P2127854 |  |
| A_30_P01031732 |  |
| A_30_P01033290 |  |
| A_51_P411917 | Gata6 |
| A_30_P01020135 |  |
| A_55_P2141729 | Snrnp27 |
| A_55_P2261772 | Lzts1 |
| A_55_P1965313 | Mctp2 |
| A_55_P1992849 | Adrb3 |
| A_51_P303089 | Ttc28 |
| A_52_P127925 | Tfec |
| A_30_P01025362 |  |
| A_55_P2144090 |  |
| A_30_P01026215 |  |
| A_55_P2049261 |  |
| A_30_P01033532 |  |
| A_51_P262111 | Foxf1 |
| A_52_P424231 | Adamts16 |
| A_55_P2057806 | Clrn2 |
| A_55_P2077618 | Csgalnact1 |
| A_55_P2146655 | LOC102638893 |
| A_30_P01019695 |  |
| A_55_P1972517 | Gm11544 |
| A_52_P166694 | Vamp1 |
| A_55_P2413964 | 1700048M11Rik |
| A_55_P2175976 | Vmn2r35 |
| A_55_P2147712 | Ctla4 |
| A_51_P188981 |  |
| A_55_P2168254 | LOC102635322 |
| A_55_P2171785 | Dnmt3aos |
| A_55_P2007467 |  |
| A_55_P1958172 | Ms4a5 |
| A_55_P2211164 | 5330406M23Rik |
| A_55_P1978258 |  |
| A_55_P2040838 | Gm14548 |
| A_55_P1968355 | Tle1 |
| A_55_P1971579 | Acox3 |
| A_55_P2041584 | F420015M19Rik |
| A_55_P2408415 | Kcnq1ot1 |
| A_30_P01029720 |  |
| A_55_P2021572 | C87414 |
| A_55_P2367415 | A630026N12Rik |
| A_51_P477736 | 4932415M13Rik |
| A_55_P2119882 |  |
| A_30_P01028837 |  |
| A_55_P1958758 | Olfr965 |
| A_55_P2058831 | Fancc |
| A_30_P01024460 |  |
| A_55_P2360501 |  |
| A_55_P1981366 | Lamc2 |
| A_66_P136132 |  |
| A_55_P2200618 | LOC552902 |
| A_30_P01032009 |  |
| A_55_P2012779 | Rnf167 |
| A_55_P1959348 | Olfr598 |
| A_55_P2151685 | Pira11 |
| A_55_P2180249 | Mtch2 |
| A_55_P2059035 | Gm13403 |
| A_30_P01026880 |  |
| A_51_P158814 | Marveld1 |
| A_66_P109731 |  |
| A_55_P2007981 | Fmn2 |
| A_55_P2096515 | Gm10706 |
| A_30_P01018987 |  |
| A_55_P2208260 | D230019N24Rik |
| A_55_P1971287 | Olfr1436 |
| A_30_P01021544 |  |
| A_55_P2275402 | 9330177L23Rik |
| A_55_P2063736 | Gp49a |
| A_30_P01022217 |  |
| A_66_P107703 | Smox |
| A_51_P173692 | Lingo4 |
| A_55_P2172852 | Ptplad2 |
| A_30_P01023847 |  |
| A_30_P01021174 |  |
| A_55_P1954985 |  |
| A_30_P01027176 |  |
| A_55_P2156800 | Ptx4 |
| A_55_P1991994 | Ptprq |
| A_55_P2084686 | Pdik1l |
| A_30_P01027527 |  |
| A_30_P01021556 |  |
| A_55_P1970144 | Slc16a14 |
| A_30_P01028537 |  |
| A_30_P01019085 |  |
| A_51_P245090 | Aqp3 |
| A_55_P1988108 | Mrc1 |
| A_30_P01024483 |  |
| A_30_P01033047 |  |
| A_30_P01022552 |  |
