# Supplementary material for: T cell–intrinsic prostaglandin E2-EP2/EP4 signaling is critical in pathogenic TH17 cell–driven inflammation
Source: J Allergy Clin Immunol. 2019 Feb;143(2):631–43. doi: 10.1016/j.jaci.2018.05.036 (PMC6354914; doi:10.1016/j.jaci.2018.05.036)
Supplement: Table E5 [file mmc7.docx]

| ProbeName | GeneSymbol |
| --- | --- |
| A_66_P121012 | Gm6602 |
| A_55_P2030354 |  |
| A_55_P2094040 |  |
| A_55_P2106043 | Bsx |
| A_30_P01030933 |  |
| A_51_P111962 | Bean1 |
| A_30_P01032018 |  |
| A_55_P2130249 | Sh3gl2 |
| A_30_P01023897 |  |
