# Supplementary material for: T cell–intrinsic prostaglandin E2-EP2/EP4 signaling is critical in pathogenic TH17 cell–driven inflammation
Source: J Allergy Clin Immunol. 2019 Feb;143(2):631–43. doi: 10.1016/j.jaci.2018.05.036 (PMC6354914; doi:10.1016/j.jaci.2018.05.036)
Supplement: Table E7 [file mmc9.docx]

| ProbeName | GeneSymbol |
| --- | --- |
| A_51_P414243 | Pomgnt2 |
| A_55_P1985764 |  |
| A_51_P436201 | Gart |
| A_51_P165934 | Phb |
| A_55_P1964648 | Btla |
| A_55_P2117146 | Pa2g4 |
| A_55_P2092310 | Phgdh |
| A_51_P313761 | Shmt2 |
| A_51_P367070 | Il9 |
| A_55_P1992838 | Socs2 |
| A_55_P1986306 | Ltv1 |
| A_55_P1971774 | Fsip1 |
| A_55_P2046877 | Foxq1 |
| A_30_P01024631 |  |
| A_51_P171075 | Csf2 |
| A_52_P518997 | Epha2 |
| A_52_P63343 | Ciart |
| A_51_P510891 | Afp |
| A_51_P514085 | Mx2 |
| A_52_P547662 | P2ry1 |
| A_51_P208922 | Stc2 |
| A_51_P351015 | Lta |
| A_51_P254234 | Chchd4 |
| A_51_P237865 | Il4 |
| A_55_P2067518 | Slc13a3 |
| A_55_P2171158 | Hmgn1 |
| A_51_P384318 | C1ra |
| A_55_P2026818 | Slc4a7 |
| A_55_P2046262 | Phgdh |
| A_51_P502152 | Slc19a1 |
| A_55_P1960157 | Bcat1 |
| A_55_P2115127 | Mphosph10 |
| A_51_P110301 | C3 |
| A_55_P2158498 | Btk |
| A_51_P377376 | Gnl3 |
| A_55_P2175767 | Rangrf |
| A_51_P430766 | Il10 |
| A_55_P1967443 |  |
| A_51_P107362 | Socs2 |
| A_52_P105537 | Nov |
| A_66_P106098 | Kif3a |
| A_66_P138319 | Acox2 |
| A_55_P2128229 |  |
| A_55_P2039027 | Speer6-ps1 |
| A_51_P115005 | Edn1 |
| A_30_P01023338 |  |
| A_55_P1973838 | Slc6a9 |
| A_55_P2100968 | Dnah7b |
| A_55_P2053459 | Timd2 |
| A_55_P1985554 | B4galt4 |
| A_55_P2038358 | Acot1 |
| A_55_P2143042 | Gm8096 |
| A_51_P207988 | Ptger4 |
| A_52_P627816 | Tgm1 |
| A_55_P2085425 | Ophn1 |
| A_55_P1959923 | Cth |
| A_55_P2127587 | Smcr8 |
| A_55_P2287611 | 4930519N06Rik |
| A_55_P1959521 | Etv4 |
| A_55_P2009708 |  |
| A_55_P1984655 | Smtnl2 |
| A_66_P132249 | Akr1c13 |
| A_55_P2186928 |  |
| A_51_P451957 | Cpne6 |
| A_55_P2009673 | Ppp1r14d |
| A_55_P2008599 | Pcx |
| A_51_P106527 | Fam195a |
| A_55_P2096867 | Gap43 |
| A_51_P317214 | Hpdl |
| A_55_P2085142 | Spp1 |
| A_55_P1960916 | Egln3 |
| A_51_P156434 | Slc25a33 |
| A_55_P2074736 | Prkar1b |
| A_65_P19933 | Zdhhc23 |
| A_55_P1964302 | Timm8a1 |
| A_55_P2110037 | Akap7 |
| A_51_P317443 | Cd3eap |
| A_51_P518163 | Rrp9 |
| A_51_P242859 | Akr1c12 |
| A_55_P2002968 | Coro2a |
| A_52_P406828 | Dkc1 |
| A_51_P291749 | Pecr |
| A_52_P131548 | Ajuba |
| A_55_P2066463 | Enah |
| A_55_P1987186 | Ttll9 |
| A_55_P2021149 | Cltb |
| A_66_P127160 | Eif2b3 |
| A_55_P2090254 | Sntg2 |
| A_55_P1953819 | Btk |
| A_52_P20906 | Twist1 |
| A_51_P187901 | Nop56 |
| A_52_P416327 | Cd226 |
| A_52_P246703 | Ak7 |
| A_30_P01021097 |  |
| A_55_P1960023 | Trmt5 |
| A_55_P2006118 | Rbp4 |
| A_51_P183894 | Fbxo15 |
| A_55_P2019312 | Car12 |
| A_55_P1988384 | Slc7a3 |
| A_65_P08971 | F3 |
| A_51_P172251 | Ifrd2 |
| A_55_P2044917 | Gpr83 |
| A_55_P1972575 | Tmeff1 |
| A_52_P651948 | Fam229b |
| A_51_P390538 | Mpeg1 |
| A_66_P116678 | Rps8 |
| A_51_P230507 | Ell2 |
| A_51_P300709 | Srm |
| A_55_P1973941 | Slc7a5 |
| A_55_P2062627 | Tmem238 |
| A_51_P170758 | Grwd1 |
| A_51_P358722 | Lancl3 |
| A_55_P2158873 | Ppid |
| A_51_P401527 | Rnmtl1 |
| A_55_P2000182 | Slc5a6 |
| A_52_P196979 | Trim66 |
| A_55_P1981994 | Krt17 |
| A_55_P1966573 | Gemin4 |
| A_51_P186053 | Rrp15 |
| A_55_P2021505 | Adra2a |
| A_66_P129111 | Nasp |
| A_51_P466162 | C1qbp |
| A_51_P468249 | Phex |
| A_55_P1996354 | Wdr31 |
| A_51_P431737 | Cth |
| A_55_P2143311 | Pdxk |
| A_51_P238383 | B4galt4 |
| A_55_P2052563 | Id1 |
| A_55_P2023912 |  |
| A_52_P622434 | Nop16 |
| A_55_P2235931 | Cacna1c |
| A_55_P2011220 | Armcx1 |
| A_55_P2128929 | Cc2d2a |
| A_55_P2061084 | Wfikkn2 |
| A_66_P136228 | Rai14 |
| A_55_P2033725 | Ascl2 |
| A_52_P359965 | Cpd |
| A_51_P114462 | Ccl17 |
| A_66_P109192 | Gm2464 |
| A_52_P400677 | AW209491 |
| A_51_P485458 | Txlna |
| A_55_P2057035 | Slmo1 |
| A_55_P2029319 | Cd70 |
| A_55_P1969481 | Hivep3 |
| A_55_P2017636 | Thbs1 |
| A_51_P268069 | Six1 |
| A_55_P1971889 | F3 |
| A_55_P2030486 | Srsf2 |
| A_55_P2002376 | Srm |
| A_52_P127682 | Dagla |
| A_51_P261164 | F2rl2 |
| A_51_P410451 | Tube1 |
| A_51_P117865 | Fam20c |
| A_51_P513992 | Spag4 |
| A_52_P452689 | Atf3 |
| A_52_P93910 | Nrp2 |
| A_51_P180974 | Prkcdbp |
| A_55_P1956847 | Nolc1 |
| A_51_P332676 | Top1mt |
| A_51_P346641 | Armcx4 |
| A_52_P245766 |  |
| A_52_P503663 | Ffar4 |
| A_52_P554703 | Gprin3 |
| A_55_P2163098 | Akr1c18 |
| A_52_P344290 | F2r |
| A_52_P596755 | Dnph1 |
| A_55_P1985840 | Mettl16 |
| A_51_P164420 | Eef1e1 |
| A_55_P2063096 | Txnrd3 |
| A_55_P1978441 | Kif3a |
| A_51_P205779 | Cd5l |
| A_51_P431734 | Fam185a |
| A_52_P543040 | Utp14a |
| A_55_P2143041 | Phgdh |
| A_55_P1992834 | Socs2 |
| A_52_P673499 | Shmt1 |
| A_55_P2186929 |  |
| A_51_P479818 | Lonrf3 |
| A_51_P335758 | Chn1 |
| A_55_P2079669 | Bcat1 |
| A_52_P387009 | Egln3 |
| A_55_P2422248 | 5730420D15Rik |
| A_52_P366047 | Rpp40 |
| A_51_P349213 | Fcrl1 |
| A_52_P459048 | 2900011O08Rik |
| A_52_P636050 | Gpatch4 |
| A_55_P2024669 | Myo6 |
| A_52_P58949 | Calcrl |
| A_52_P97699 | D430019H16Rik |
| A_51_P164939 | Tmem150a |
| A_55_P2016249 | Ppid |
| A_55_P1995497 | Atad3a |
| A_51_P134812 | Chac1 |
| A_55_P2201822 | Naf1 |
| A_52_P191633 | Fam71b |
| A_51_P164203 | Nme4 |
| A_51_P296487 | Lss |
| A_55_P1997390 | Cpd |
| A_55_P1952638 | Fgd6 |
| A_55_P2009213 | Pde7a |
| A_55_P1990261 | Chchd6 |
| A_55_P1959748 | Asns |
| A_30_P01031178 |  |
| A_51_P401987 | Tmem37 |
| A_51_P104392 | Rpp25 |
| A_52_P574214 | Rrp1b |
| A_55_P2085574 | Nfix |
| A_55_P2367803 | Il2 |
| A_51_P432199 | Sap30 |
| A_51_P449935 | Ftsj3 |
| A_51_P378789 | Cxcl13 |
| A_55_P2099790 | Nefh |
| A_55_P2010788 | Slc6a9 |
| A_51_P125567 | Mettl13 |
| A_52_P481279 | Drc1 |
| A_51_P301636 | Kazn |
| A_55_P2193512 | Cd226 |
| A_55_P1952744 | Timm8a1 |
| A_51_P430630 | Gpr33 |
| A_55_P2115225 | Fap |
| A_30_P01021389 |  |
| A_51_P487690 | Ifi44 |
| A_52_P152631 | Tmem17 |
| A_55_P2005783 | Ifih1 |
| A_52_P527800 | Emilin2 |
| A_30_P01030879 |  |
| A_51_P156438 | Slc25a33 |
| A_55_P1978052 | Pet112 |
| A_52_P248403 |  |
| A_55_P2033680 |  |
| A_55_P2168823 |  |
| A_55_P2083449 | Spryd7 |
| A_55_P2042486 | Dpysl3 |
| A_55_P2080168 | Dgkk |
| A_51_P205326 | Fam198a |
| A_55_P1989102 | Hmgn1 |
| A_55_P2137527 | Fam183b |
| A_55_P2116744 | Xirp1 |
| A_55_P2184370 | LOC102641088 |
| A_51_P455647 | Car2 |
| A_55_P1964093 | Rangrf |
| A_55_P1973995 | Gm6756 |
| A_55_P2128085 | LOC102641654 |
| A_52_P853177 | Angptl2 |
| A_52_P625640 | Trim9 |
| A_55_P1958857 | Nek6 |
| A_52_P231075 | Fcrls |
| A_51_P401907 | Gm5483 |
| A_55_P1992490 | Scg2 |
| A_55_P1958464 |  |
| A_51_P150302 | Crtam |
| A_55_P2134800 | Cinp |
