# Supplementary material for: T cell–intrinsic prostaglandin E2-EP2/EP4 signaling is critical in pathogenic TH17 cell–driven inflammation
Source: J Allergy Clin Immunol. 2019 Feb;143(2):631–43. doi: 10.1016/j.jaci.2018.05.036 (PMC6354914; doi:10.1016/j.jaci.2018.05.036)
Supplement: Table E8 [file mmc10.docx]

| ProbeName | GeneSymbol |
| --- | --- |
| A_51_P385099 | Tnf |
| A_55_P1967514 | Dnah7a |
| A_55_P2103698 | Isg15 |
| A_55_P2122075 | Pdcd1lg2 |
| A_55_P2165234 | 2300005B03Rik |
| A_55_P2187141 | Pdcd1lg2 |
| A_51_P160344 | Cenpv |
| A_55_P2012989 | Slamf7 |
| A_55_P2163138 | Tm4sf5 |
| A_52_P157880 |  |
| A_55_P2138386 | Il5 |
| A_51_P242166 | Lap3 |
| A_55_P2205858 | Col6a5 |
| A_55_P2079020 | Snhg7os |
| A_66_P101942 | Gm9706 |
| A_55_P2179463 | Tnfsf8 |
| A_51_P496432 | Acsl1 |
| A_51_P188281 | Myf5 |
| A_51_P113178 | Fam212b |
| A_55_P2125972 | Gorasp1 |
| A_51_P272563 | Naa25 |
| A_66_P117543 |  |
| A_55_P1964559 | Smarca5-ps |
| A_51_P187018 | Magohb |
| A_52_P649561 | Heg1 |
| A_51_P270426 | Egr4 |
| A_55_P2085974 | Igf1 |
| A_52_P344978 |  |
| A_51_P184728 | Cnksr3 |
| A_66_P105460 | Ccdc14 |
| A_51_P273609 | Itpka |
| A_30_P01028030 |  |
| A_66_P128631 | Cinp |
| A_55_P1962214 | Kpna3 |
| A_55_P2070825 | Nudt5 |
| A_55_P1993019 |  |
| A_55_P1992814 | Shq1 |
| A_55_P2130965 |  |
| A_51_P308844 | Nrn1 |
| A_52_P13897 | Hook1 |
| A_52_P639774 | Gart |
| A_66_P128537 | Isg15 |
| A_51_P228768 | Slfn3 |
| A_51_P516085 | Dntt |
| A_55_P2134804 | Cinp |
| A_51_P169624 | Taf3 |
| A_55_P2180839 | Il13 |
| A_30_P01030169 |  |
| A_30_P01018645 |  |
| A_55_P2084703 | Acaca |
| A_51_P234627 | Nubpl |
| A_55_P2172182 | Olfr1138 |
| A_55_P2004452 | Tceal8 |
| A_55_P2128144 | Il19 |
| A_51_P200561 | 4930506M07Rik |
| A_51_P165098 | Gga2 |
| A_52_P987201 | Pdzrn4 |
| A_55_P1964960 | Il33 |
| A_55_P2123037 | Olfr553 |
| A_51_P301215 | Knop1 |
| A_30_P01025391 |  |
| A_51_P232399 | Acy3 |
| A_55_P2039684 | Gpr34 |
| A_51_P347452 | Htatsf1 |
| A_51_P215530 | Rnf180 |
| A_52_P131836 | Bysl |
| A_55_P2082806 | Trib1 |
| A_55_P2095880 | Nfix |
