# Supplementary material for: T cell–intrinsic prostaglandin E2-EP2/EP4 signaling is critical in pathogenic TH17 cell–driven inflammation
Source: J Allergy Clin Immunol. 2019 Feb;143(2):631–43. doi: 10.1016/j.jaci.2018.05.036 (PMC6354914; doi:10.1016/j.jaci.2018.05.036)
Supplement: Table E9 [file mmc11.docx]

| GO ACCESSION | GO Term | p-value | corrected p-value | -logP | gene |
| --- | --- | --- | --- | --- | --- |
| GO:0031347 | regulation of defense response | 2.87E-08 | 1.64E-04 | 3.78E+00 | Il1r1 Il17f Foxf1 Calca Il17a Il23r |
| GO:0032101 | regulation of response to external stimulus | 1.03E-07 | 1.97E-04 | 3.71E+00 | Il1r1 Il17f Foxf1 Calca Il17a Il23r |
| GO:0050727 | regulation of inflammatory response | 7.57E-08 | 1.97E-04 | 3.71E+00 | Il1r1 Il17f Foxf1 Calca Il17a |
| GO:0080134 | regulation of response to stress | 1.28E-06 | 0.00183381 | 2.74E+00 | Il1r1 Il17f Foxf1 Calca Il17a Il23r |
| GO:1900017 | positive regulation of cytokine production involved in inflammatory response | 7.20E-06 | 0.008231741 | 2.08E+00 | Il17f Il17a |
| GO:1900015 | regulation of cytokine production involved in inflammatory response | 1.54E-05 | 0.014684372 | 1.83E+00 | Il17f Il17a |
| GO:0006954 | inflammatory response | 2.83E-05 | 0.020216491 | 1.69E+00 | Il17f Calca Il17a Il23r |
| GO:0071345 | cellular response to cytokine stimulus | 2.55E-05 | 0.020216491 | 1.69E+00 | Il1r1 Foxf1 Il17a Il23r |
| GO:0031328 | positive regulation of cellular biosynthetic process | 6.72E-05 | 0.029578676 | 1.53E+00 | Nr2e3 Il17f Mycn Foxf1 Calca Il17a |
| GO:0034097 | response to cytokine | 5.94E-05 | 0.029578676 | 1.53E+00 | Il1r1 Foxf1 Il17a Il23r |
| GO:0045935 | positive regulation of nucleobase-containing compound metabolic process | 4.74E-05 | 0.029578676 | 1.53E+00 | Nr2e3 Il17f Mycn Foxf1 Calca Il17a |
| GO:0045944\|GO:0010552\|GO:0045817 | positive regulation of transcription from RNA polymerase II promoter | 6.37E-05 | 0.029578676 | 1.53E+00 | Nr2e3 Il17f Mycn Foxf1 Il17a |
| GO:0051173 | positive regulation of nitrogen compound metabolic process | 6.47E-05 | 0.029578676 | 1.53E+00 | Nr2e3 Il17f Mycn Foxf1 Calca Il17a |
| GO:0009891 | positive regulation of biosynthetic process | 7.62E-05 | 0.03114245 | 1.51E+00 | Nr2e3 Il17f Mycn Foxf1 Calca Il17a |
| GO:0033993 | response to lipid | 8.48E-05 | 0.032340873 | 1.49E+00 | Nr2e3 Rbp1 Il17a Il23r |
