# Supplementary material for: T cell–intrinsic prostaglandin E2-EP2/EP4 signaling is critical in pathogenic TH17 cell–driven inflammation
Source: J Allergy Clin Immunol. 2019 Feb;143(2):631–43. doi: 10.1016/j.jaci.2018.05.036 (PMC6354914; doi:10.1016/j.jaci.2018.05.036)
Supplement: Table E10 [file mmc12.docx]

| GO ACCESSION | GO Term | p-value | corrected p-value | -logP | gene |
| --- | --- | --- | --- | --- | --- |
| GO:0005615 | extracellular space | 8.63E-06 | 0.0250513 | 1.6011702 | Lum Tgfbi Wnt6 Timp1 Crispld2 |
| GO:0031012 | extracellular matrix | 8.10E-06 | 0.0250513 | 1.6011702 | Lum Tgfbi Wnt6 Il1rn Timp1 Enpp2 Il22 |
