# Supplementary material for: T cell–intrinsic prostaglandin E2-EP2/EP4 signaling is critical in pathogenic TH17 cell–driven inflammation
Source: J Allergy Clin Immunol. 2019 Feb;143(2):631–43. doi: 10.1016/j.jaci.2018.05.036 (PMC6354914; doi:10.1016/j.jaci.2018.05.036)
Supplement: Table E11 [file mmc13.docx]

| GO ACCESSION | GO Term | p-value | corrected p-value | -logP |  |
| --- | --- | --- | --- | --- | --- |
| GO:0044459 | plasma membrane part | 3.77E-10 | 1.73E-05 | 4.76E+00 | Cacna1s Synpo Tgfb3 Ms4a4b Cd59a Hc Il7r Selp Cd27 Ccr2 Lag3 Cd160 Sytl2 Gpnmb Gabbr1 Vmn1r24 Gpc3 Sema4f Cd24a Klrc1 Pde4b Ms4a1 Ntrk3 S1pr1 Itgb3 Slc22a22 Nt5e Cxcr4 Pla2g4f Ifnlr1 Camk2n1 Abca1 Plaur Atp6v0d2 Dcc Tdgf1 Shc4 Rasa3 Catsper3 Atp2b2 Sgcg Il12rb2 Abcb4 Grm1 Slc8a3 Shank2 Cacna1h Gria4 Ms4a4b Scn3b Klra4 Klra1 Klrc1 Ntrk3 Cx3cr1 Slc17a3 Sell Pde4d Fasl Lzts3 Slco6b1 Trpm6 Kcng1 Ntrk3 Sgip1 Vmn1r203 Tmprss11e Kcnmb4 Adra1b P2rx4 Adora2a Sipa1l1 Gabra3 Klrd1 Klri2 Smo Slc52a3 Sdcbp Wwc1 Cyth3 Klrc3 Dmd Akap7 Clstn3 Cacnb2 Catsperd Kcnma1 Igf1r Cd33 Kctd12 Atp6v0d2 Tlr4 |
| GO:0071944 | cell periphery | 1.27E-08 | 2.92E-04 | 3.53E+00 | Cacna1s Gpr65 Arrdc4 Synpo Tgfb3 Cysltr2 Ms4a4b Cd59a Hc Amigo2 Slc46a2 Il7r Abcb9 Ptprz1 Selp Cd27 Lat2 Ccr2 Lag3 Cd160 Sytl2 Tmod1 Cpm Olfr1414 Grb7 Tspan2 Rab19 Olfr983 Paqr8 Gpnmb Khdc3 Cldn10 Gabbr1 Olfr959 Il1r2 Vmn1r24 Gpr171 Prnp Sptb Slc16a9 Bst1 Gabbr1 Ramp1 Vcl Tbc1d30 Fzd10 Gpc3 Sema4f Cd24a Magi1 Klrc1 Svil Pde4b Fam110c Ms4a1 Ntrk3 Klra15 S1pr1 Itgb3 Sla2 Slc22a22 Nt5e Cxcr4 Pla2g4f Ifnlr1 Camk2n1 Abca1 Plaur Atp6v0d2 Txk Ramp3 Igflr1 Lsp1 Klra16 P2ry14 Pigr Dcc Cxcr6 Tdgf1 Shc4 Rasa3 Catsper3 Atp2b2 Olfr668 Sgcg Il12rb2 Abcb4 Grm1 Slc8a3 Shank2 Cacna1h Gria4 Ms4a4b Vmn2r48 Rasa3 Ptprz1 Scn3b Cd300lf Klra4 Klra1 Klrc1 Olfr1446 Ntrk3 Dgkb Cx3cr1 Vmn2r91 Slc17a3 Sell Pde4d Atp2b3 Olfr54 Wwox Fasl Cap2 Samhd1 Lzts3 Slc16a5 Slco6b1 Gpr146 Tspan2 Cdcp1 Trpm6 Piezo2 Gpr82 Klra23 Efna2 Cx3cr1 Kcng1 Arl4d Fgfr1 Sgip1 Pde6a Inadl Vipr1 Vmn1r203 Ptpn13 Vmn2r60 Tmprss11e Olfr1320 Nid1 Ifitm1 Kcnmb4 Olfr701 Adra1b Ramp3 P2rx4 Vmn2r96 Pde4b Art4 Adora2a Sipa1l1 Gabra3 Klrd1 Klri2 Smo Prrt1 Slc52a3 Slc16a2 Sdcbp Wwc1 Ermn Rasgrp2 Ifitm1 Cyth3 Zan Cat Klrc3 Sema6c Gpr114 Thsd7a Ntn1 Cxcr6 Dmd Cacna1s Ptpn13 Akap7 Olfr566 Clstn3 Mrgprg Cfh Cacnb2 Dgkg Snap23 Caln1 Catsperd Kcnma1 Pde4b Pde4d Olfr726 Igf1r Cd33 Cdh18 Kctd12 Atp6v0d2 Tlr4 Itgb3 |
| GO:0005886\|GO:0005904 | plasma membrane | 7.45E-08 | 0.0011393 | 2.94E+00 | Cacna1s Gpr65 Arrdc4 Synpo Tgfb3 Cysltr2 Ms4a4b Cd59a Hc Amigo2 Slc46a2 Il7r Abcb9 Ptprz1 Selp Cd27 Lat2 Ccr2 Lag3 Cd160 Sytl2 Cpm Olfr1414 Grb7 Tspan2 Rab19 Olfr983 Paqr8 Gpnmb Cldn10 Gabbr1 Olfr959 Il1r2 Vmn1r24 Gpr171 Prnp Sptb Slc16a9 Bst1 Gabbr1 Ramp1 Vcl Tbc1d30 Fzd10 Gpc3 Sema4f Cd24a Magi1 Klrc1 Svil Pde4b Ms4a1 Ntrk3 Klra15 S1pr1 Itgb3 Sla2 Slc22a22 Nt5e Cxcr4 Pla2g4f Ifnlr1 Camk2n1 Abca1 Plaur Atp6v0d2 Txk Ramp3 Igflr1 Lsp1 Klra16 P2ry14 Pigr Dcc Cxcr6 Tdgf1 Shc4 Rasa3 Catsper3 Atp2b2 Olfr668 Sgcg Il12rb2 Abcb4 Grm1 Slc8a3 Shank2 Cacna1h Gria4 Ms4a4b Vmn2r48 Rasa3 Ptprz1 Scn3b Cd300lf Klra4 Klra1 Klrc1 Olfr1446 Ntrk3 Dgkb Cx3cr1 Vmn2r91 Slc17a3 Sell Pde4d Atp2b3 Olfr54 Wwox Fasl Cap2 Samhd1 Lzts3 Slc16a5 Slco6b1 Gpr146 Tspan2 Cdcp1 Trpm6 Piezo2 Gpr82 Klra23 Efna2 Cx3cr1 Kcng1 Ntrk3 Arl4d Fgfr1 Sgip1 Pde6a Inadl Vipr1 Vmn1r203 Ptpn13 Vmn2r60 Tmprss11e Olfr1320 Ifitm1 Kcnmb4 Olfr701 Adra1b Ramp3 P2rx4 Vmn2r96 Pde4b Art4 Adora2a Sipa1l1 Gabra3 Klrd1 Klri2 Smo Prrt1 Slc52a3 Slc16a2 Sdcbp Wwc1 Rasgrp2 Ifitm1 Cyth3 Zan Cat Klrc3 Sema6c Gpr114 Thsd7a Cxcr6 Dmd Cacna1s Ptpn13 Akap7 Olfr566 Clstn3 Mrgprg Cfh Cacnb2 Dgkg Snap23 Caln1 Catsperd Kcnma1 Pde4b Pde4d Olfr726 Igf1r Cd33 Cdh18 Kctd12 Atp6v0d2 Tlr4 Itgb3 |
| GO:0009897 | external side of plasma membrane | 2.16E-07 | 0.0024761 | 2.61E+00 | Cd59a Il7r Selp Cd27 Ccr2 Lag3 Cd24a Klrc1 Ms4a1 S1pr1 Itgb3 Cxcr4 Abca1 Il12rb2 Klra4 Klra1 Klrc1 Sell Fasl Klrd1 Klrc3 Kcnma1 Cd33 Tlr4 Itgb3 |
| GO:1902531\|GO:0010627 | regulation of intracellular signal transduction | 1.46E-06 | 0.0134296 | 1.87E+00 | Dusp10 Rapgef3 Tgfb3 Cysltr2 Phlpp1 Tnip3 Selp Cd27 Sesn3 Xdh Tgfb2 Il20ra Ecm1 Sfrp4 Prnp Hgf Il18r1 Bcl6 Fzd10 Cd24a Gcnt2 Fam110c Ntrk3 Itgb3 Sla2 Hes5 Abca1 Dcc Tdgf1 Agpat9 Rasa3 Grm1 Rasa3 Dusp7 Ntrk3 Pde4d Tnfaip3 Ntrk3 Fgfr1 C1qtnf3 Dusp5 Rora Il18r1 Adra1b P2rx4 Sipa1l1 Klf4 Sipa1l2 Wwc1 Sipa1l2 Rasgrp2 Cyth3 Cat Cmya5 Dmd Akap7 Tgfb2 Pde4d Ecm1 Igf1r Tlr4 Itgb3 |
| GO:0002376 | immune system process | 3.03E-06 | 0.0213073 | 1.67E+00 | Klf2 Hc Slc46a2 Il7r Tnip3 Ptprz1 Selp Cd27 Lat2 Ccr2 Epas1 Tgfb2 Procr Tspan2 Foxj1 Sptb Il18r1 Bcl6 Gcnt1 Gpc3 Cxcl3 Cd24a Tcf7 Pde4b Ms4a1 S1pr1 Sla2 Tlx1 Cxcr4 Ifnlr1 Txk P2ry14 Pigr Tdgf1 Slc8a3 Ctse Ptprz1 Cd300lf Cx3cr1 Pde4d Fasl Myb Samhd1 Tspan2 Gab3 Efna2 Tnfaip3 Cx3cr1 Rora Pglyrp1 Gcnt1 Ifitm1 Il18r1 Foxp1 Pde4b Tcf7 Eml1 Spib Klf4 Serpinb9 Eml1 Ifitm1 Aicda Eomes Cfh Ifnar1 Ifnar1 Pde4b Tgfb2 Pde4d Cblb Runx2 Igf1r Tlr4 |
| GO:0004896\|GO:0004907 | cytokine receptor activity | 3.25E-06 | 0.0213073 | 1.67E+00 | Il7r Ccr2 Il1r2 Il18r1 Il18rap Cxcr4 Ifnlr1 Cxcr6 Il12rb2 Cx3cr1 Cxcr6 Ifnar1 |
| GO:0009653 | anatomical structure morphogenesis | 5.97E-06 | 0.0339258 | 1.47E+00 | Cacna1s Rapgef3 Tgfb3 Klf2 Serpinb5 Il7r Ptprz1 Selp Ptprb Ccr2 Epas1 Nyap2 mod1 Tgfb2 Ecm1 Foxj1 Sfrp4 Spaca1 Matn2 Hgf Col4a2 Bcl6 Ramp1 Vcl Gcnt1 Map2 Gpc3 Sema4f Tcf7 Ablim1 Nr4a2 S1pr1 Itgb3 Tlx1 Cxcr4 Murc Dcc Tdgf1 Atp2b2 Spaca1 Sall3 Gcm1 Ptprz1 Nr2e1 Cryaa Tead1 Wwox Fasl Cap2 Efna2 Fgfr1 Tbx6 Dusp5 Rora Gcnt1 Ifitm1 Hmx2 Foxp1 Adora2a Tcf7 Ctnnd2 Tmem106b Smo Klf4 Slitrk4 Ermn Flrt3 Ifitm1 Gcm1 Sema6c Thsd7a Ntn1 Dmd Eomes Runx2 Igf1r Crispld1 |
| GO:0005891 | voltage-gated calcium channel complex | 6.66E-06 | 0.0339258 | 1.47E+00 | Cacna1s Pde4b Catsper3 Cacna1h Pde4d Cacnb2 Catsperd |
| GO:0051239 | regulation of multicellular organismal process | 8.09E-06 | 0.0371204 | 1.43E+00 | Dusp10 Rapgef3 Tgfb3 Cysltr2 Cd59a Klf2 Hc Slc46a2 Il7r Ptprz1 Selp Lama4 Cd27 Ccr2 Epas1 Aspa Lag3 Sytl2 Proc Xdh Tgfb2 Il20ra Procr Ecm1 Foxj1 Sfrp4 Prnp Hgf Col4a2 Il18r1 Tg Bcl6 Gpc3 Sema4f Cd24a Gcnt2 Pde4b Nr4a2 Ntrk3 S1pr1 Itgb3 Cxcr4 Hes5 Txk Dcc Tdgf1 Il12rb2 Grm1 Ptprz1 Scn3b Nr2e1 Mfap4 Ntrk3 Cx3cr1 Pde4d Fasl Myb Tnfaip3 Fgfr1 Sgip1 Tbx6 C1qtnf3 Rora Pglyrp1 Tnnt3 Adra1b P2rx4 Foxp1 Pde4b Adora2a Sipa1l1 Smo Klf4 Cmya5 Ntn1 Dmd Btg1 Eomes Kcnma1 Ifnar1 Cblb Runx2 Ccnd1 Tlr4 |
| GO:0007166 | cell surface receptor signaling pathway | 8.93E-06 | 0.0372139 | 1.43E+00 | Tgfb3 Il7r Wisp1 Cd27 Lat2 Ccr2 Lag3 Clnk Tgfb2 Il20ra Sfrp4 Il1r2 Hgf Il18r1 Fzd10 Cd24a Tcf7 Gcnt2 Pde4b Ntrk3 Il18rap Itgb3 Cxcr4 Ifnlr1 Hes5 Abca1 Plaur Txk P2ry14 Pigr Dcc Cxcr6 Tdgf1 Adamts14 Il12rb2 Grm1 Gria4 Adam34 Ntrk3 Cx3cr1 Wwox Fasl Kremen1 Efna2 Cx3cr1 Ntrk3 Fgfr1 Vipr1 Tle2 P2rx4 Pde4b Adora2a Tcf7 Sipa1l1 Smo Wisp1 Eya2 Gpr114 Cxcr6 Akap7 Ifnar1 Cblb Runx2 Igf1r Ccnd1 Tlr4 |
| GO:0048583 | regulation of response to stimulus | 1.02E-05 | 0.0389147 | 1.41E+00 | Dusp10 Rapgef3 Tgfb3 Cysltr2 Cd59a Hc Phlpp1 Il7r Tnip3 Abcb9 Selp Cd27 Lat2 Ccr2 Sesn3 Lag3 Proc Xdh Tgfb2 Il20ra Grb7 Foxj1 Sfrp4 Prnp Hgf Il18r1 Bcl6 Ramp1 Fzd10 Gpc3 Cd24a Gcnt2 Pde4b Fam110c Ntrk3 S1pr1 Itgb3 Sla2 Nt5e Cxcr4 Ifnlr1 Hes5 Abca1 Txk Ramp3 Pigr Dcc Tdgf1 Agpat9 Rasa3 Bicc1 Sall3 Grm1 Dusp7 Cx3cr1 Sell Pde4d Wwox Fasl Myb Samhd1 Tnfaip3 Fgfr1 Zfyve28 C1qtnf3 Dusp5 Padi2 Rora Pglyrp1 Tle2 Adra1b Ramp3 P2rx4 Adora2a Sipa1l1 Klf4 Sipa1l2 Wwc1 Rasgrp2 Cyth3 Cat Cmya5 Dmd Akap7 Cfh Pde4d Cblb Runx2 Ecm1 Igf1r Ccnd1 Kctd12 Tlr4 |
| GO:0098552 | side of membrane | 1.38E-05 | 0.0486405 | 1.31E+00 | Cd59a Il7r Selp Cd27 Ccr2 Lag3 Cd24a Klrc1 Ms4a1 S1pr1 Itgb3 Cxcr4 Abca1 Rasa3 Il12rb2 Klra4 Klra1 Klrc1 Sell Fasl Klrd1 Cyth3 Klrc3 Kcnma1 Cd33 Tlr4 |
