# Supplementary figures and images for: T cell–intrinsic prostaglandin E2-EP2/EP4 signaling is critical in pathogenic TH17 cell–driven inflammation
Source: J Allergy Clin Immunol. 2019 Feb;143(2):631–43. doi: 10.1016/j.jaci.2018.05.036 (PMC6354914; doi:10.1016/j.jaci.2018.05.036)

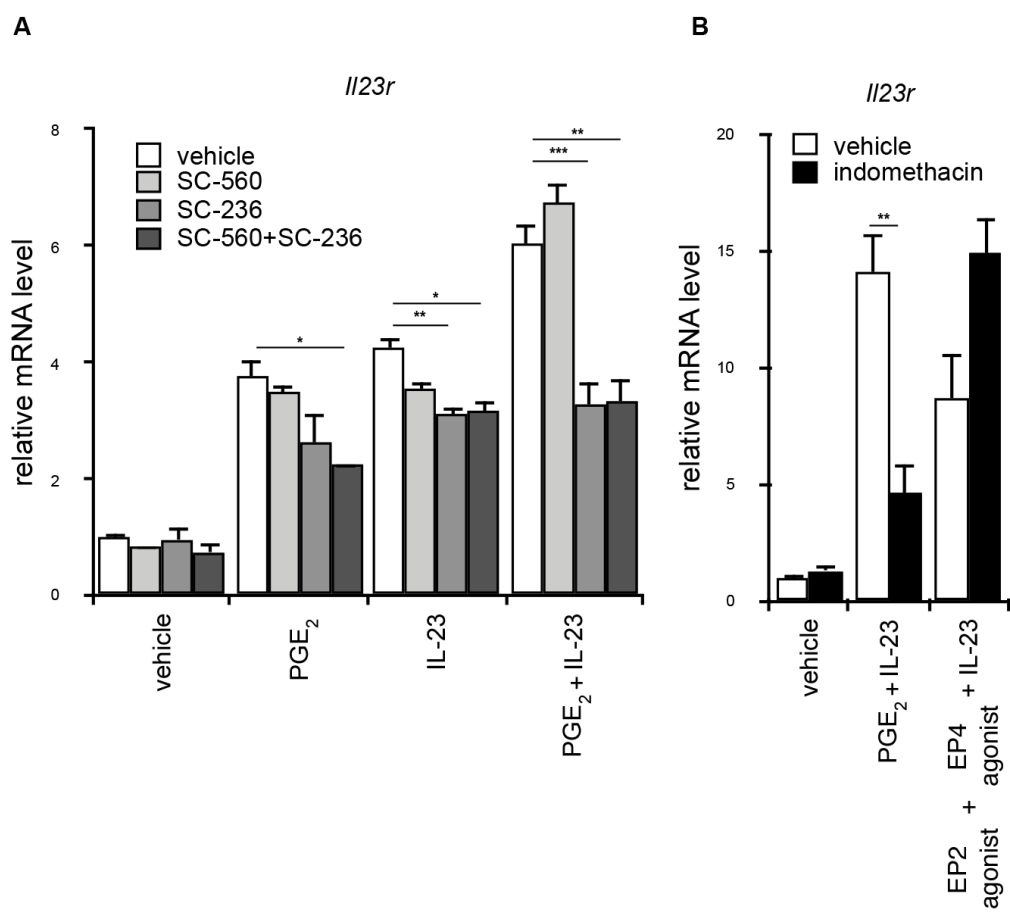

**Figure E1**

Supplement: Fig E1 [file mmc14.pdf]

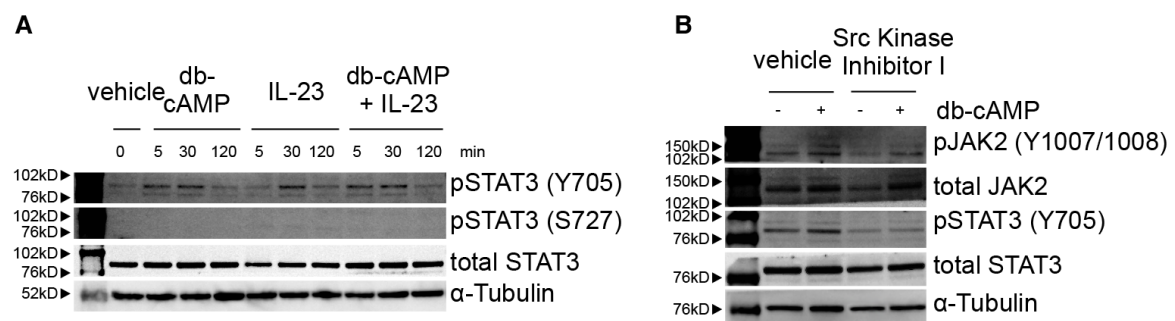

**Figure E2**

Supplement: Fig E2 [file mmc15.pdf]

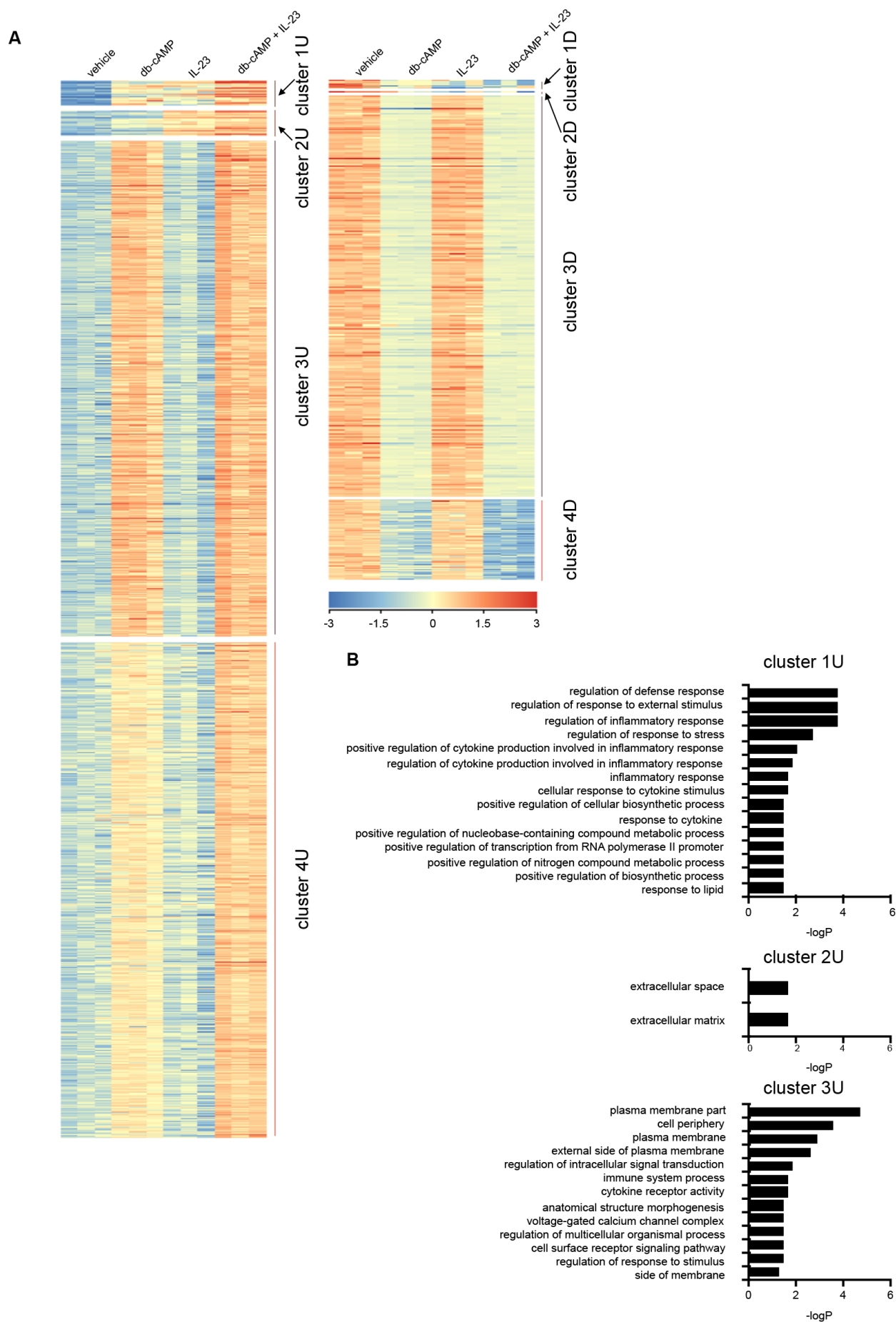

**Figure E3**

Supplement: Fig E3 [file mmc16.pdf]

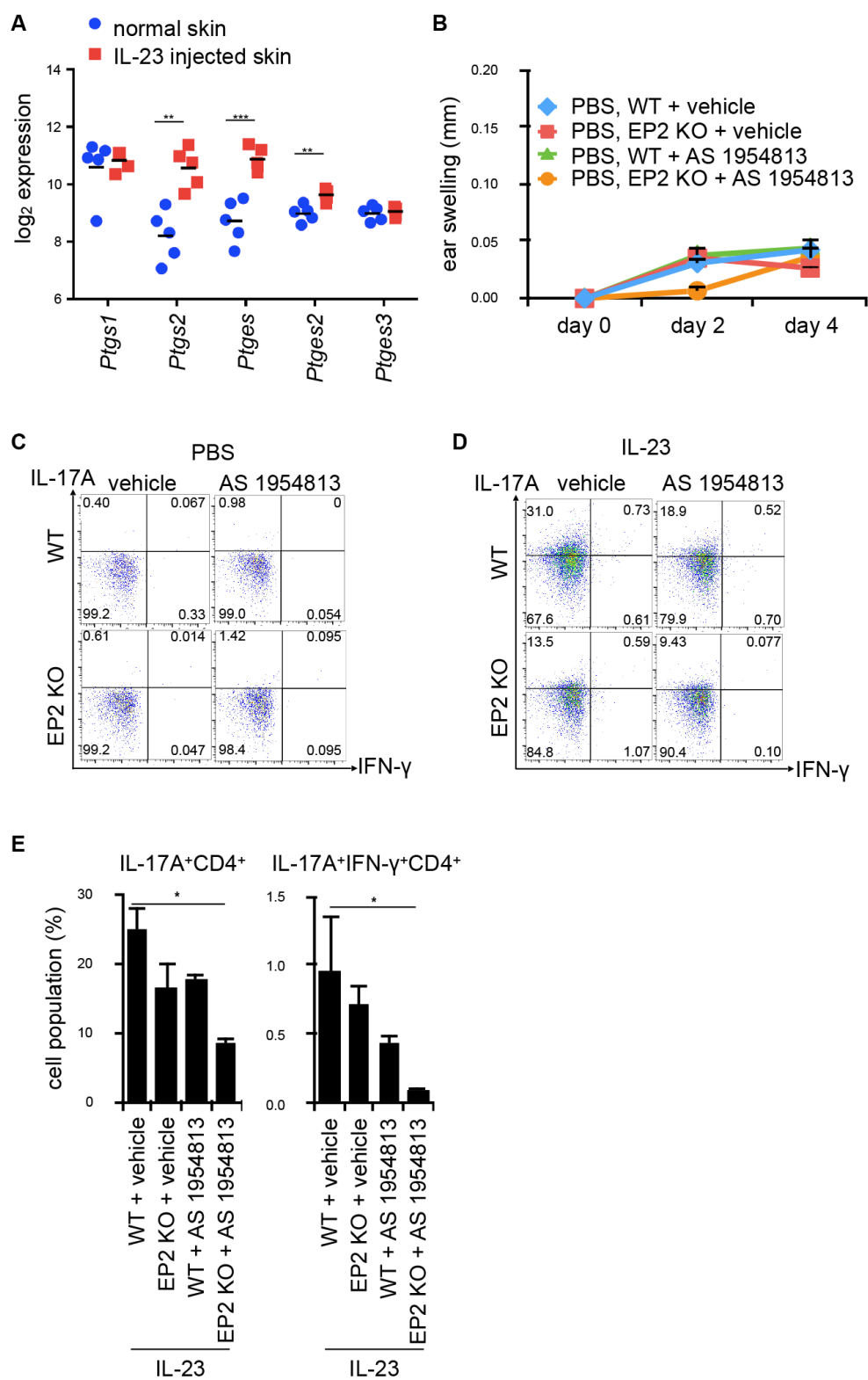

Figure E4

Supplement: Fig E4 [file mmc17.pdf]

A

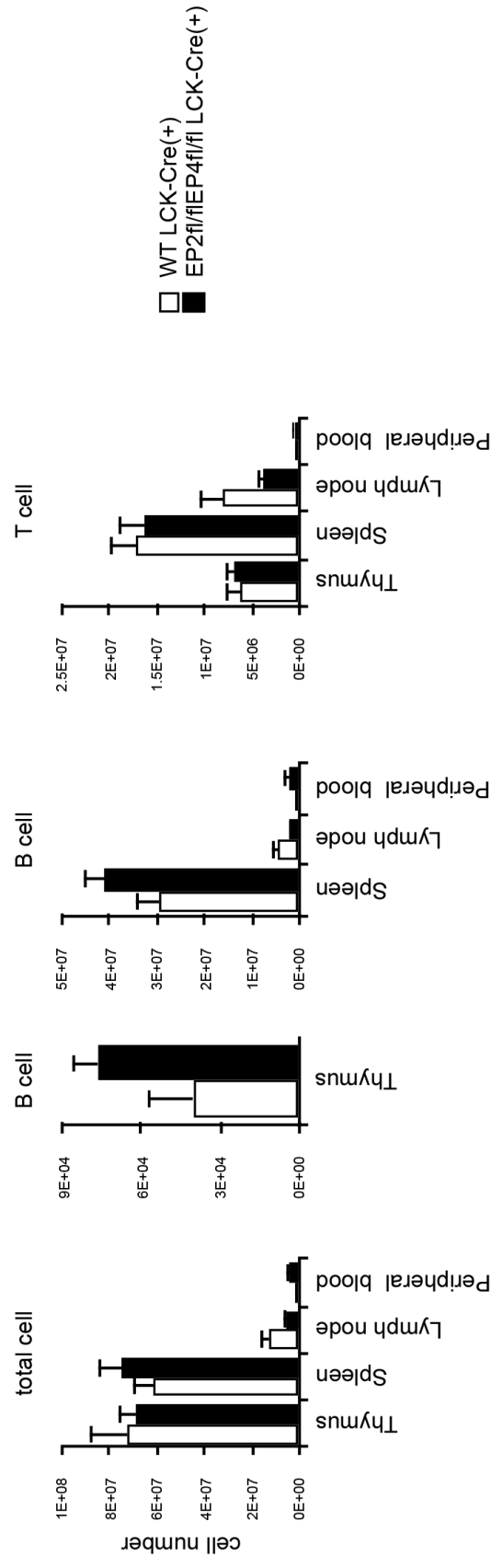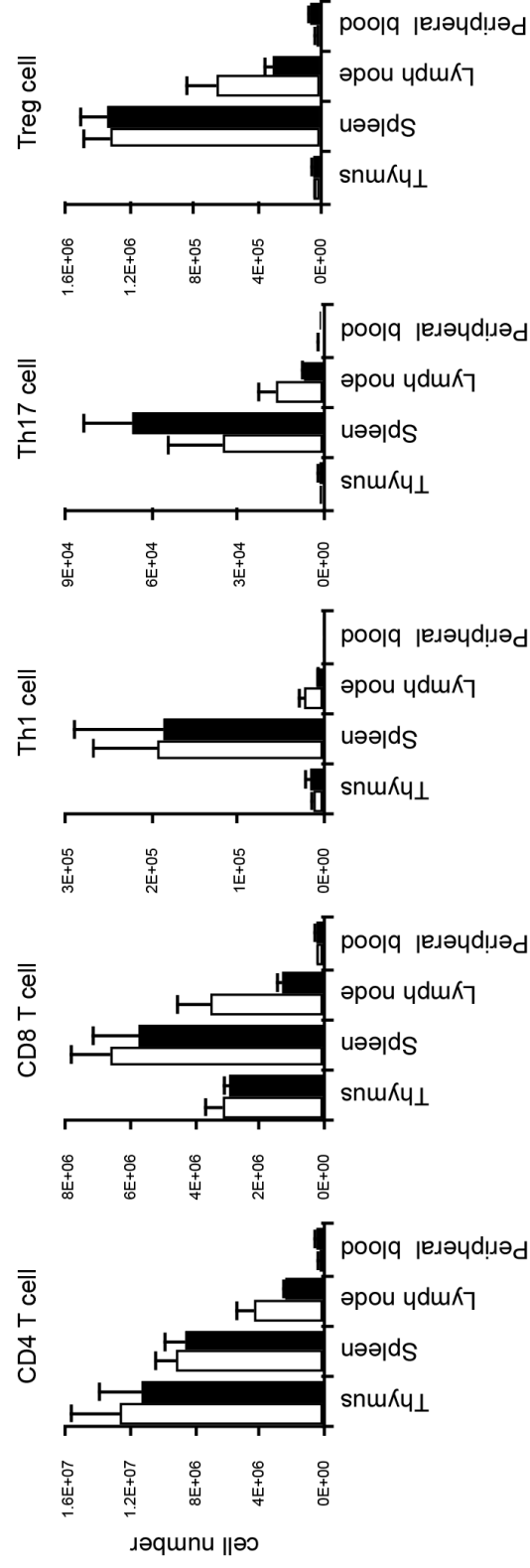

Figure E5

Supplement: Fig E5 [file mmc18.pdf]

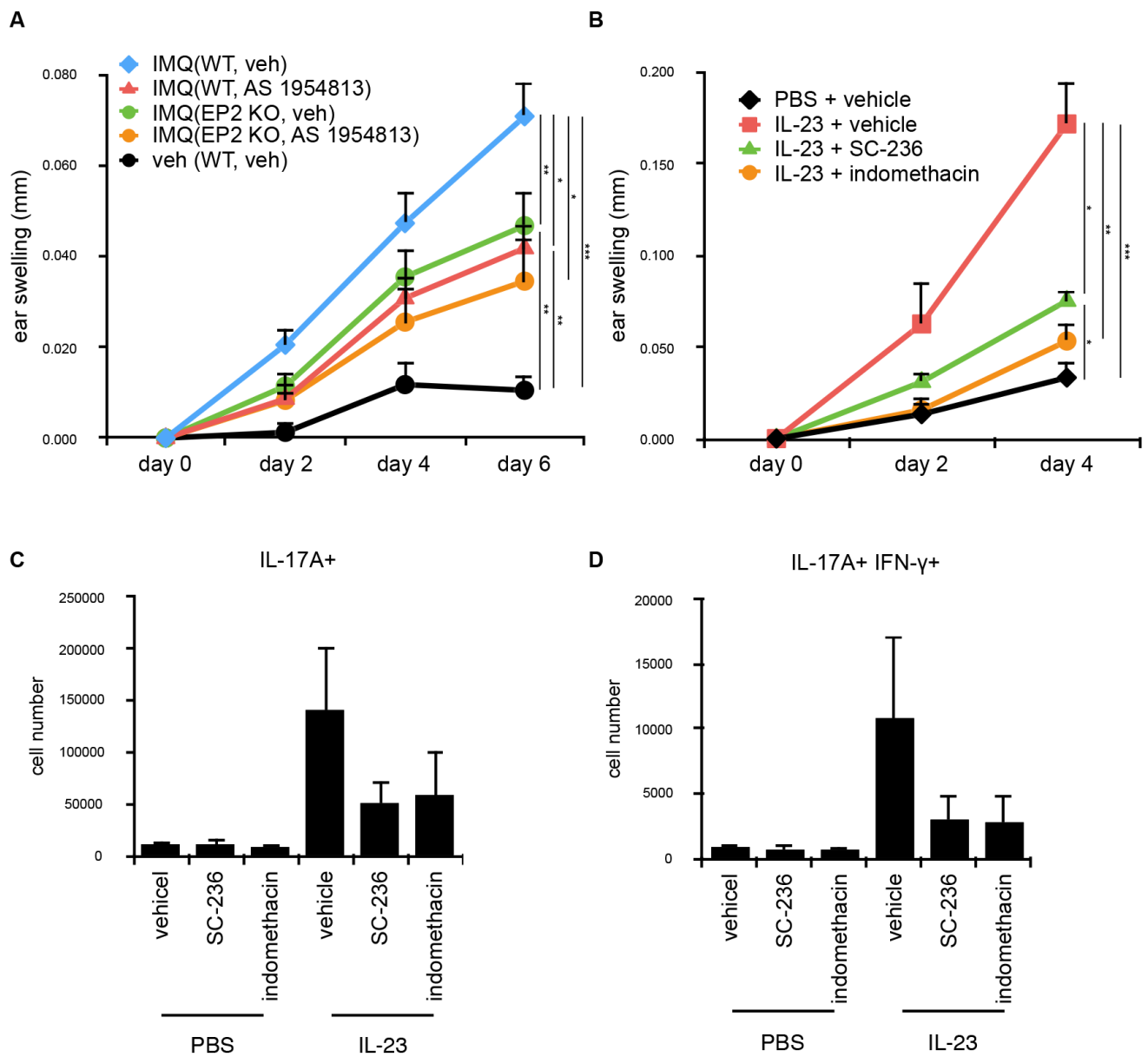

Figure E6

Supplement: Fig E6 [file mmc19.pdf]

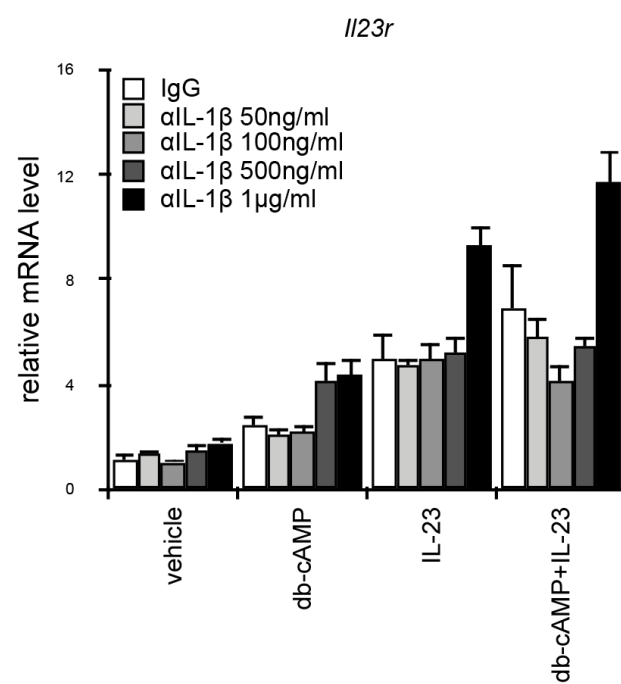

Figure E7

Supplement: Fig E7 [file mmc20.pdf]
